# Potential effectiveness of prophylactic HPV immunization for men who have sex with men in the Netherlands: A multi-model approach

**DOI:** 10.1371/journal.pmed.1002756

**Published:** 2019-03-04

**Authors:** Johannes A. Bogaards, Sofie H. Mooij, Maria Xiridou, Maarten F. Schim van der Loeff

**Affiliations:** 1 Centre for Infectious Disease Control, National Institute for Public Health and the Environment (RIVM), Bilthoven, The Netherlands; 2 Department of Epidemiology & Biostatistics, Amsterdam UMC, location VUmc, Vrije Universiteit Amsterdam, Amsterdam, The Netherlands; 3 Cluster of Infectious Diseases, Public Health Service of Amsterdam (GGD), Amsterdam, The Netherlands; 4 Amsterdam Infection & Immunity Institute, Amsterdam UMC, University of Amsterdam, Amsterdam, The Netherlands; The Catalan Institute of Oncology, SPAIN

## Abstract

**Background:**

Men who have sex with men (MSM) are at high risk for anal cancer, primarily related to human papillomavirus genotype 16 (HPV16) infections. At 8.5 per 100,000 per year, the incidence rate of anal cancer among MSM is similar to that of cervical cancer among adult women in the Netherlands. However, MSM are not included in most HPV vaccination programs. We explored the potential effectiveness of prophylactic immunization in reducing anogenital HPV16 transmission among MSM in the Netherlands.

**Methods and findings:**

We developed a range of mathematical models for penile–anal HPV16 transmission, varying in sexual contact structure and natural history of infection, to provide robust and plausible predictions about the effectiveness of targeted vaccination. Models were informed by an observational cohort study among MSM in Amsterdam, 2010–2013. Parameters on sexual behavior and HPV16 infections were obtained by fitting the models to data from 461 HIV-negative study participants, considered representative of the local MSM population. We assumed 85% efficacy of vaccination against future HPV16 infections as reported for HIV-negative MSM, and age-specific uptake rates similar to those for hepatitis B vaccination among MSM in the Netherlands. Targeted vaccination was contrasted with vaccination of 12-year-old boys at 40% uptake in base-case scenarios, and we also considered the effectiveness of a combined strategy. Offering vaccine to MSM without age restrictions resulted in a model-averaged 27.3% reduction (90% prediction interval [PI] 11.9%–37.5%) in prevalence of anal HPV16 infections, assuming similar uptake among MSM as achieved for hepatitis B vaccination. The predicted reduction improved to 46.1% (90% PI 21.8%–62.4%) if uptake rates among MSM were doubled. The reductions in HPV16 infection prevalence were mostly achieved within 30 years of a targeted immunization campaign, during which they exceeded those induced by vaccinating 40% of preadolescent boys, if started simultaneously. The reduction in anal HPV16 prevalence amounted to 74.8% (90% PI 59.8%–93.0%) under a combined vaccination strategy. HPV16 prevalence reductions mostly exceeded vaccine coverage projections among MSM, illustrating the efficiency of prophylactic immunization even when the HPV vaccine is given after sexual debut. Mode of protection was identified as the key limitation to potential effectiveness of targeted vaccination, as the projected reductions were strongly reduced if we assumed no protection against future infections in recipients with prevalent infection or infection-derived immunity at the time of immunization. Unverified limitations of our study include the sparsity of data to inform the models, the omission of oral sex in transmission to the penile or anal site, and the restriction that our modeling results apply primarily to HIV-negative MSM.

**Conclusions:**

Our findings suggest that targeted vaccination may generate considerable reductions in anogenital HPV16 infections among MSM, and has the potential to accelerate anal cancer prevention, especially when combined with sex-neutral vaccination in preadolescence.

## Abstract 2

Johannes Bogaards and colleagues reveal the benefits of including men who have sex with men in a HPV vaccination program to reduce incidence of anal cancer.

## Author summary

**Why was this study done?:**

- Anal and genital human papillomavirus (HPV) infections are sexually transmitted and may cause cancer in the anogenital area.
- HPV vaccines protect against cancer by lowering the risk of getting infected with HPV, and are especially effective when given before becoming sexually active.
- Men who have sex with men (MSM) are at high risk for anal cancer, but are not included in most HPV vaccination programs.
- Decisions about their inclusion need to be informed by transmission models, but this is a challenge due to uncertainties regarding vaccine efficacy in those already exposed to HPV, and regarding HPV infection dynamics among MSM.

**What did the researchers do and find?:**

- To give robust and plausible predictions about the effectiveness of targeted vaccination, we developed various models for HPV transmission among MSM that were parameterized using data from a Dutch cohort study.
- We assessed the effectiveness of various vaccination strategies targeting MSM or 12-year-old boys or a combination thereof, and assuming vaccine uptake in targeted campaigns comparable to that of hepatitis B vaccine among MSM in the Netherlands.
- In the models, targeted vaccination reduced the occurrence of anogenital HPV infections by around 30% after 40 years, with a range from 10% to 50%, depending on the recruitment of MSM into targeted campaigns and on the assumed mode of vaccine protection.
- This figure increased to 75% after 60 years when targeted vaccination was combined with sex-neutral vaccination in preadolescence, assuming 40% uptake among 12-year-old boys and 85% efficacy against future HPV16 infections in MSM.

**What do these findings mean?:**

- Our results are helpful for prioritizing male HPV vaccination, especially when deciding on the implementation of a selective campaign among MSM.
- Offering HPV vaccine to sexually experienced MSM need not impede the efficiency of targeted vaccination, if vaccination protects against future HPV16 infections.
- Targeted vaccination deserves consideration, at least temporarily, to protect adult MSM at high risk for anal cancer.

## Introduction

Sexually transmitted oncogenic types of human papillomavirus (HPV) are known as the causative agents of cervical cancer [1–3]. They may also cause cancers in males, notably penile cancer, anal cancer, and a subset of head and neck cancers [2]. Relative to heterosexual males, men who have sex with men (MSM) are at increased risk for HPV-related cancers, especially for anal cancer [3]. With an estimated incidence of 8.5 per 100,000 per year, the incidence rate of anal cancer among MSM is similar to that of cervical cancer among adult women in the Netherlands [4].

In many countries, including the Netherlands, HPV-related disease prevention efforts are still entirely directed at females, through vaccination of preadolescent girls and screening for cervical cancer [2,4,5]. Over time, heterosexual males may receive indirect benefit from female vaccination through herd immunity, but MSM will not [6,7]. Vaccination of preadolescent boys along with girls can ultimately lead to control of HPV-related diseases in men and women alike, but might not constitute the most efficient use of resources [8–10]. Moreover, preadolescent vaccination will not protect currently active MSM, who might benefit from a targeted immunization campaign [11–14]. However, the effectiveness of selective vaccination targeting MSM past sexual debut could be hampered by prior exposure to HPV vaccine types [15,16].

Extrapolating the population-level effectiveness of selective vaccination from vaccine trials is difficult, for several reasons. First, it is not clear which estimates of vaccine efficacy to use; intention-to-treat estimates are difficult to apply outside the specific study settings (e.g., to age groups other than those included in a trial), whereas per-protocol estimates require a correct interpretation outside the protocol conditions. Additionally, the population-level effectiveness of a selective vaccination program targeting high-risk individuals strongly depends on the infrastructure by which vaccines can be delivered to this group, and the implications in terms of vaccine coverage by age. Finally, herd effects are expected to play a major role in determining the ultimate impact of targeted prevention efforts, and their assessment typically relies on mathematical modeling.

The purpose of this paper is to explore, by means of mathematical modeling, the potential effectiveness of a targeted immunization campaign among MSM in the Netherlands. We focus on reductions in anogenital HPV genotype 16 (HPV16) infections, as HPV16 causes the majority of anogenital cancers in males—i.e., around 85% of anal HPV-related cancers [17] and over 60% of penile HPV-related cancers [18]—and is included in all registered HPV vaccines [2]. To assess the temporal benefit of targeted vaccination, post-vaccination dynamics in HPV16 prevalence among MSM are contrasted to those induced by sex-neutral vaccination (i.e., vaccination of boys in addition to girls) in preadolescence.

## Methods

We developed a range of mathematical models for penile–anal HPV16 transmission to assess the potential effectiveness of selective vaccination of MSM. Throughout we assumed that MSM acquire penile infections via insertive anal intercourse, whereas anal infections are acquired through receptive anal intercourse. The models differed in terms of sexual contact structure of the MSM population and assumptions regarding the natural history of HPV16 infection. Sexual contact parameters were estimated, whenever possible, from self-administered questionnaire data regarding sociodemographic characteristics and recent sexual behavior among 778 MSM (median age: 40 years, 5th–95th percentile 28–61 years) participating in the H2M study [19]. This is an observational cohort study on HIV and HPV infections in MSM recruited in Amsterdam in 2010–2011 and followed every 3–6 months for at least 2 years.

### Dynamic population model

We constructed a deterministic dynamic population model of MSM by estimating age-specific rates of entering and exiting the population of individuals forming same-sex male partnerships (S1–S4 Figs). To account for penile-to-anal and anal-to-penile transmission, we incorporated sexual behavior by distinguishing insertive anal intercourse from receptive anal intercourse. The probabilities of engaging in either insertive, receptive, or both insertive and receptive anal sex within a partnership were obtained by fitting a mixture model to self-reported activities with anal sex partners in the last 6 months (S5 Fig). We modeled partner acquisition on the basis of self-reported numbers of anal sex partners in the last 6 months by HIV-negative H2M study participants, considered representative of the MSM population as validated by comparison to HIV incidence rates in the local community and an internet survey on sexual behavior among MSM throughout the Netherlands (S1 Text). To account for heterogeneity in partner acquisition rates in the model population, we considered 18 distinct settings of sexual contact structure for penile–anal HPV16 transmission, namely 3 distributions according to level of sexual activity (conditional on the age-specific mean and variance in age-specific contact rates) times 3 degrees of assortative mixing with respect to sexual activity times 2 degrees of assortative mixing with respect to preference for insertive/receptive anal sex (Table 1).

**Table 1:** Dynamic population model parameters and vaccine efficacy assumptions.

Notation^±^	Description	Units	Value or distribution	Justification
ϑ(*a*)	Rate of entering MSM population (by age)	Number/year	Skew-lognormal density function with location = 0.30, scale = 2.85, and skewness = 1.42	Fitted to self-reported age of first anal sex with a male partner by H2M study participants
μ(*a*)	Rate of exiting at-risk population (by age)^¶^	Per year	Weibull hazard function with shape = 3, location = 30, and scale = 25	Calibrated to obtain MSM population distribution akin to H2M study population*
$\bar{c}(a)$	Average rate of partner acquisition (by age)	Number/year	Parabolic function with maximum = 9.3 at 40 years	Fitted to estimated number of new anal sex partners in last 6 months (H2M study; Schorer Monitor^†^)
$p_{k}$	Fraction with sexual activity indexed by *k*	Percent	Variable according to model: {80%; 20%}, {90%; 10%}, or {60%; 30%; 10%}	Constrained by age-specific mean and coefficient of variation (cv = 1.86) of partner acquisition rate
$q_{i}$	Fraction with sexual preference indexed by *i*	Percent	Preference for either insertive, receptive, or both insertive and receptive anal sex: {13%; 13%; 74%}	Mixture model fitted to self-reported activities with anal sex partners by H2M study participants^††^
*p*	Conditional probability of having both insertive and receptive anal sex with the same partner	—	0.60 (applicable to MSM with preference for both insertive and receptive anal sex)	Mixture model fitted to self-reported activities with anal sex partners by H2M study participants^††^
ϕ	Assortativity with regard to preference for insertive/receptive anal sex	—	Variable according to model: 0.15 or 0.67	Chosen values denote random (0.15) and moderately strong assortative (0.67) mixing
ϵ	Assortativity with regard to sexual activity	—	Variable according to model: 0, 0.33, or 0.67	Chosen values denote random (0), weakly assortative (0.33), and moderately strong assortative (0.67) mixing
σ(*a*,*t*)	Rate of vaccine uptake (by age, after start of vaccination campaign)	Per year	Gamma function (from age ≥ 15 years) with shape = 2, scale = 2, and maximum = 0.02 in base-case scenarios	Fitted to hepatitis B vaccine uptake rates among 15- to 70-year-old MSM up to 2010 in the Netherlands
π	Probability of deriving vaccine-induced protection against future infections	—	0.85 in base-case scenarios; 1.0 in sensitivity analyses if fully susceptible at immunization, otherwise 0.0	Based on 85% incidence rate reduction of ≥6-month infection in male HPV vaccine trials (per-protocol)^§^
ϖ	Infection rate reduction induced by successful vaccination	—	1.0 in base-case scenarios; 0.85 in sensitivity analyses	Based on 85% incidence rate reduction of ≥6-month infection in male HPV vaccine trials (per-protocol)^§^

^±^See mathematical descriptions for further explanation (S1 Text) and use in HPV16 transmission models (S2 Text).

^¶^The at-risk population consists of MSM forming new sexual partnerships; those in steady monogamous relationships are no longer at risk.

*Approximated to the empirical density from age 45 years onward; density at younger age is distorted due to under-recruitment in the H2M study of MSM with recent sexual debut (see S3 Fig).

^†^The Schorer Monitor is a large-scale internet survey investigating health, well-being, and sexuality among MSM throughout the Netherlands (see S1 Text).

^††^The mixture model assumes identical proportions for MSM having strict preference for either insertive or receptive anal sex (see S1 Text).

^§^As reported for vaccine-type infections detectable for at least 6 months in quadrivalent HPV vaccine trials among 16- to 26-year-old HIV-negative MSM (see [15,16]).

HPV, human papillomavirus; HPV16, human papillomavirus genotype 16; MSM, men who have sex with men.

### Natural history of HPV16 infection

We stratified the model population into separate compartments by penile and anal HPV16 infection status. Individuals could be susceptible or infected at either or both anatomic sites separately, yielding a minimum of 4 compartments: {*SS*,*SI*,*IS*,*II*}, with *SS* denoting the proportion of the population susceptible for both penile and anal infection, *SI* denoting the proportion susceptible for penile infection while infected at the anal site, and so forth. We defined separate (age- and time-dependent) infection hazards for acquiring HPV16 infection at the penile site only, at the anal site only, and at both sites from the same partner. We distinguished between penile-to-anal transmissibility, β_01_, defined as the per-partnership probability of HPV16 transmission from the penis to the anus when engaging in insertive anal sex, and anal-to-penile transmissibility, β_10_, defined as the per-partnership probability of HPV16 transmission from the anus to the penis when engaging in receptive anal sex (S2 Text).

In order to provide robust and plausible predictions about the effectiveness of targeted vaccination in light of structural model uncertainties, we constructed several models for the natural history of penile and anal HPV16 infections. The simplest model included only the minimum of 4 compartments {*SS*,*SI*,*IS*,*II*}, with independent clearance of penile and anal infections at a constant rate γ_10_ and γ_01_, respectively. In a modified version, we considered separate compartments with persistent infections developing at a rate ζ_10_ and ζ_01_ and clearing at a rate ξ_10_ < γ_10_ and ξ_01_ < γ_01_ for penile and anal infections, respectively.

Further modifications were obtained by considering natural immunity or latency. For natural immunity, we considered both the possibility of systemic and local immunity, and in the latter case we also considered the options that immunity would only be induced at the penile or anal site. In addition, we considered separate scenarios for immunity following clearance in all instances, in 1:3 instances, or in 1:10 instances. In all scenarios, natural immunity could be lost at a constant rate κ, assumed similar for the penile and anal site in case of local immunity. Latency was incorporated in a similar fashion; either all, 1:3, or 1:10 incident infections would turn into latent infections, with the remainder becoming either susceptible again or systemically immune. Reactivation of latent infections was modeled at a rate ϱ, assumed to be similar for both anatomic sites.

Parameters related to HPV16 infection and transmission were obtained by fitting the models to HPV16 prevalence and clearance among the 461 H2M study participants who provided penile and anal samples and were HIV-negative for the entire follow-up [20]. An overview of the models used in prediction is given in Table 2, with mathematical descriptions in S2 Text. For each model, parameter estimates were obtained by an approximate maximum-likelihood procedure (S3 Text), consisting of separate optimization of progression and clearance parameters from longitudinal data, and conditional optimization of other parameters from site-specific HPV16 infection prevalence at study baseline.

**Table 2:** Descriptions of the HPV16 natural history models included in predictions.

Model acronym*	Clearance^±^	Natural immunity	Latency	Fractions that develop immunity or latency	Penile-to-anal transmissibility^§^	Anal-to-penile transmissibility^§^	Waning of natural immunity^¶^	Reactivation of latent infections^¶^
SIS	Exponential	No	No	Not applicable	0.149 (0.116–0.191)	0.015 (0.011–0.016)	Not applicable	Not applicable
SISPS	Biphasic	No	No	Not applicable	0.115 (0.089–0.155)	0.010 (0.007–0.011)	Not applicable	Not applicable
SIRS	Exponential	Systemic	No	100%	0.428 (0.225–0.999)	0.040 (0.036–0.062)	0.966/year	Not applicable
SIS33RS	Exponential	Systemic	No	33%	0.372 (0.200–0.999)	0.032 (0.030–0.043)	0.33/year^¤^	Not applicable
SIS10RS	Exponential	Systemic	No	10%	0.330 (0.176–0.842)	0.027 (0.023–0.036)	0.10/year^¤^	Not applicable
SISPminRS	Biphasic	Systemic	No	Only those that clear a persistent infection^†^	0.179 (0.110–0.340)	0.013 (0.009–0.035)	0.819/year	Not applicable
SISPmaxRS	Biphasic	Systemic	No	Only those that clear a persistent infection^††^	0.191 (0.118–0.321)	0.015 (0.011–0.035)	0.886/year	Not applicable
SIR[local]S	Exponential	Local; both penile and anal	No	100%	0.379 (0.201–0.999)	0.026 (0.022–0.033)	0.989/year	Not applicable
SIS33R[local]S	Exponential	Local; both penile and anal	No	33%	0.367 (0.195–0.825)	0.025 (0.020–0.032)	0.33/year^¤^	Not applicable
SIS10R[local]S	Exponential	Local; both penile and anal	No	10%	0.313 (0.175–0.628)	0.022 (0.017–0.028)	0.10/year^¤^	Not applicable
SIR[penile]S	Exponential	Local; only penile	No	100% upon clearing a penile infection	0.156 (0.130–0.209)	0.029 (0.021–0.228)	0.710/year	Not applicable
SIS33R[penile]S	Exponential	Local; only penile	No	33% upon clearing a penile infection	0.150 (0.129–0.203)	0.022 (0.018–0.032)	0.319/year	Not applicable
SIS10R[penile]S	Exponential	Local; only penile	No	10% upon clearing a penile infection	0.152 (0.127–0.199)	0.020 (0.016–0.025)	0.10/year^¤^	Not applicable
SIR[anal]S	Exponential	Local; only anal	No	100% upon clearing an anal infection	0.388 (0.180–0.994)	0.018 (0.014–0.020)	0.990/year	Not applicable
SIS33R[anal]S	Exponential	Local; only anal	No	33% upon clearing an anal infection	0.359 (0.176–0.839)	0.018 (0.013–0.020)	0.33/year^¤^	Not applicable
SIS10R[anal]S	Exponential	Local; only anal	No	10% upon clearing an anal infection	0.351 (0.162–0.637)	0.017 (0.013–0.019)	0.10/year^¤^	Not applicable
SIL	Exponential	No	Yes	100%	0.033 (0.020–0.070)	0.002 (0.002–0.003)	Not applicable	1.0/year^¤^
SIS33L	Latent mixture	No	Yes	33%	0.115 (0.045–0.446)	0.005 (0.003–0.006)	Not applicable	0.546/year
SIS10L	Latent mixture	No	Yes	10%	0.446 (0.194–0.999)	0.011 (0.004–0.018)	Not applicable	0.341/year
SIR33L	Latent mixture	Systemic	Yes	Immunity: 67% Latency: 33%	0.919 (0.262–0.999)	0.031 (0.025–0.100)	0 (no waning)	0.845/year

*Capital letters in model acronyms have the following meaning: S = susceptible; I = infected; P = persistently infected; R = resistant to infection; L = latently infected.

^±^Estimates of site-specific progression and clearance parameters are given in S3 Text.

^§^Median (minimum–maximum) per-partnership transmission probability across 18 settings of sexual contact structure.

^¶^Mean rate across 18 settings of sexual contact structure.

^¤^Upper bound of predefined estimation interval.

^†^In case of dual persistent infections, development of systemic immunity is determined by the anatomic site with slowest clearance.

^††^In case of dual persistent infections, development of systemic immunity is determined by the anatomic site with fastest clearance.

HPV16, human papillomavirus genotype 16.

### Vaccination scenarios

In evaluating the potential effectiveness of targeted vaccination, we considered offering vaccine to MSM in the following age groups: ≤26 years (based on evidence from vaccine trials) [15,16], ≤40 years (recommended for selective vaccination of MSM in the UK) [13], and all ages (without an upper age for eligibility). In base-case analysis, we assumed age-specific uptake rates similar to those for hepatitis B (HepB) vaccine among MSM throughout the Netherlands [21]. In 2002, the Netherlands initiated a selective vaccination program targeting groups at high risk for HepB infection, including MSM. Because HepB vaccination was added to the childhood vaccination program in 2011, we restricted estimates of age-specific annual vaccination rate to estimated HepB vaccine uptake rates among 15- to 70-year-old MSM over the period up to 2010 (S4 Text). We also considered a scenario where HPV vaccine acceptance among MSM was double that of HepB vaccine, by using 2-fold increased age-specific uptake rates (S9 Fig). Effectiveness of targeted vaccination was contrasted with sex-neutral preadolescent vaccination by assuming 40% of MSM were vaccinated against HPV16 upon entrance into the sexually active population. The value 40% was based on the estimated uptake among boys in countries with sex-neutral HPV immunization programs [22]. In sensitivity analyses, we also examined a combined strategy of preadolescent and targeted vaccination under base-case assumptions—i.e., 40% uptake among boys and uptake among MSM similar to that for the HepB vaccine—and a scenario of 80% uptake among 12-year-old boys (S4 Text).

Prophylactic efficacy was taken from a quadrivalent HPV vaccine trial conducted among 16- to 26-year-old males [15]. We based our analysis on the 85.6% (97.5% CI 73.4–92.9) incidence rate reduction of infection detected for ≥6 months with vaccine-type HPV in the per-protocol population, consisting of participants who were seronegative on day 1 and PCR-negative from day 1 through month 7 for the relevant vaccine types. Prophylactic efficacy was incorporated in the transmission models by assuming that 85% of vaccinees became fully protected against future HPV16 infections and 15% were unaffected by vaccination. In the latter category, vaccine recipients remained fully susceptible if so at the time of immunization, or reverted back to susceptibility upon loss of natural immunity. This interpretation of vaccine efficacy by “take” rather than “degree” has also been used in assessing the population-level impact of sex-neutral vaccination by heterosexual HPV transmission models [10,23]. In sensitivity analysis, we considered the conservative scenario of restricted efficacy, where HPV16 infection hazards were reduced by 85%, i.e., “leaky” protection by degree, and only if vaccinees were fully susceptible at the time of immunization (Table 1). Following previous models, we assumed 98% efficacy in the scenario of preadolescent boys’ vaccination [9,10]. Note that vaccine efficacy against reactivation of latent infections was not assumed in any scenario.

### Model-averaged predictions

For each vaccination scenario, we formed a model-averaged prediction of the reduction in anogenital HPV16 prevalence among MSM that may be achieved via prophylactic immunization. This started by calculating Akaike weights for each model under consideration [24], based on the relative quality of all candidate models with respect to H2M study data (S4 Text). As some natural history models can be viewed as a subset of more generic models (e.g., models with natural immunity converge to those without in case of short-lasting immunity), we employed upper bounds on parameter estimates for loss of immunity κ and reactivation rate ϱ in order to avoid duplicates in the set of candidate models. Models with negligible weight, i.e., without empirical support, were omitted from further consideration.

Eventually, we included 360 models (20 natural history models combined with 18 settings of sexual contact structure) per vaccination scenario in assessing the effectiveness of vaccination. We calculated the site-specific HPV16 prevalence prior to vaccination and its (relative) reduction, specifically after 25 years and at the post-vaccination equilibrium, and summarized results using the Akaike-weighted predictions with 90% prediction intervals (PIs), defined as the 5th–95th percentile range of the 360 models.

## Results

**Fig 1:**
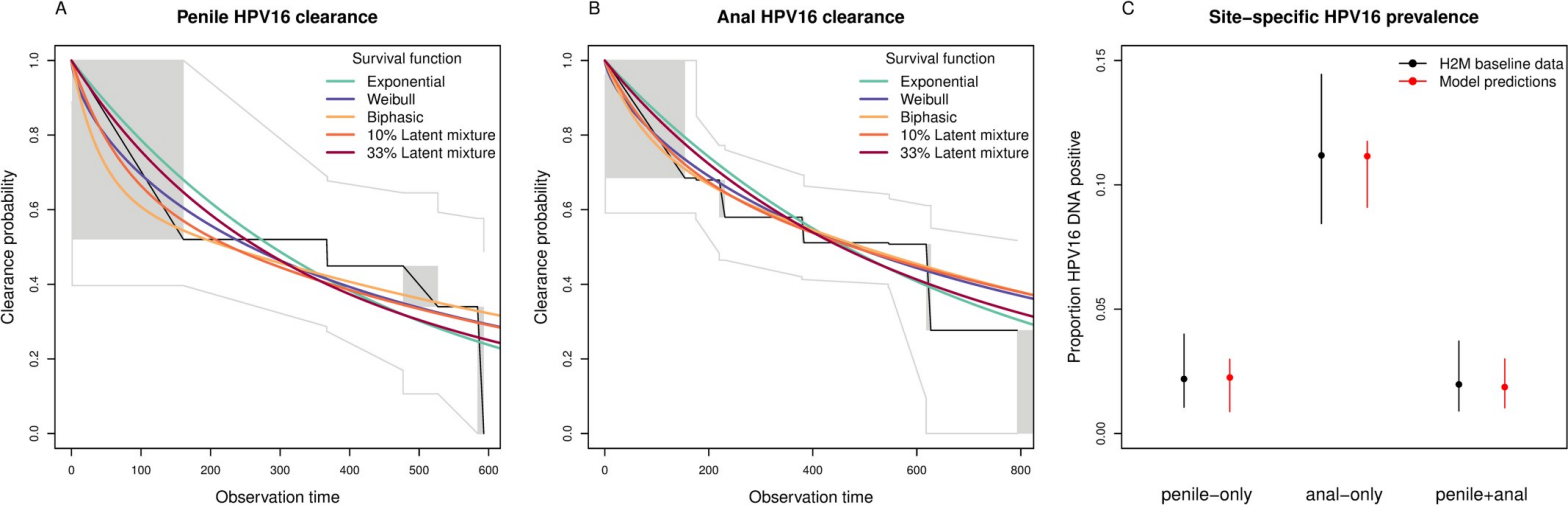
Outcomes of approximate maximum-likelihood fitting procedure. This procedure consisted of separate likelihood optimization of progression and clearance parameters from event times of (A) penile and (B) anal infection clearance (time in days), and conditional estimation of remaining model parameters from multinomial likelihood optimization of (C) site-specific infection prevalence. Non-parametric survival functions (black lines; grey boxes) with 95% confidence limits (grey lines) in (A) and (B) are from a generalization of Kaplan–Meier estimates to interval-censored data [25]. Colored lines refer to various model-based survival functions fitted to interval-censored data (S3 Text). H2M baseline data in (C) are summarized as means with 95% binomial confidence intervals from 461 HIV-negative MSM. Model predictions (age-matched to H2M study participants) are given as Akaike-weighted averages with the minimum–maximum range across 360 models. HPV16, human papillomavirus genotype 16.

Patterns of site-specific HPV16 infection prevalence and clearance among HIV-negative H2M study participants were compatible with a range of mathematical models for penile–anal HPV16 transmission (Fig 1). Site-specific transmission probabilities varied widely across the models (Table 2), but penile-to-anal transmissibility almost invariably exceeded anal-to-penile transmissibility. Models that assumed a higher degree of natural immunity generally required increased transmissibility to reproduce the observed prevalence of penile and anal HPV16 infections. In addition, penile-to-anal transmissibility was higher when presuming a stronger degree of assortative mixing with respect to sexual activity (S7 Fig).

**Fig 2:**
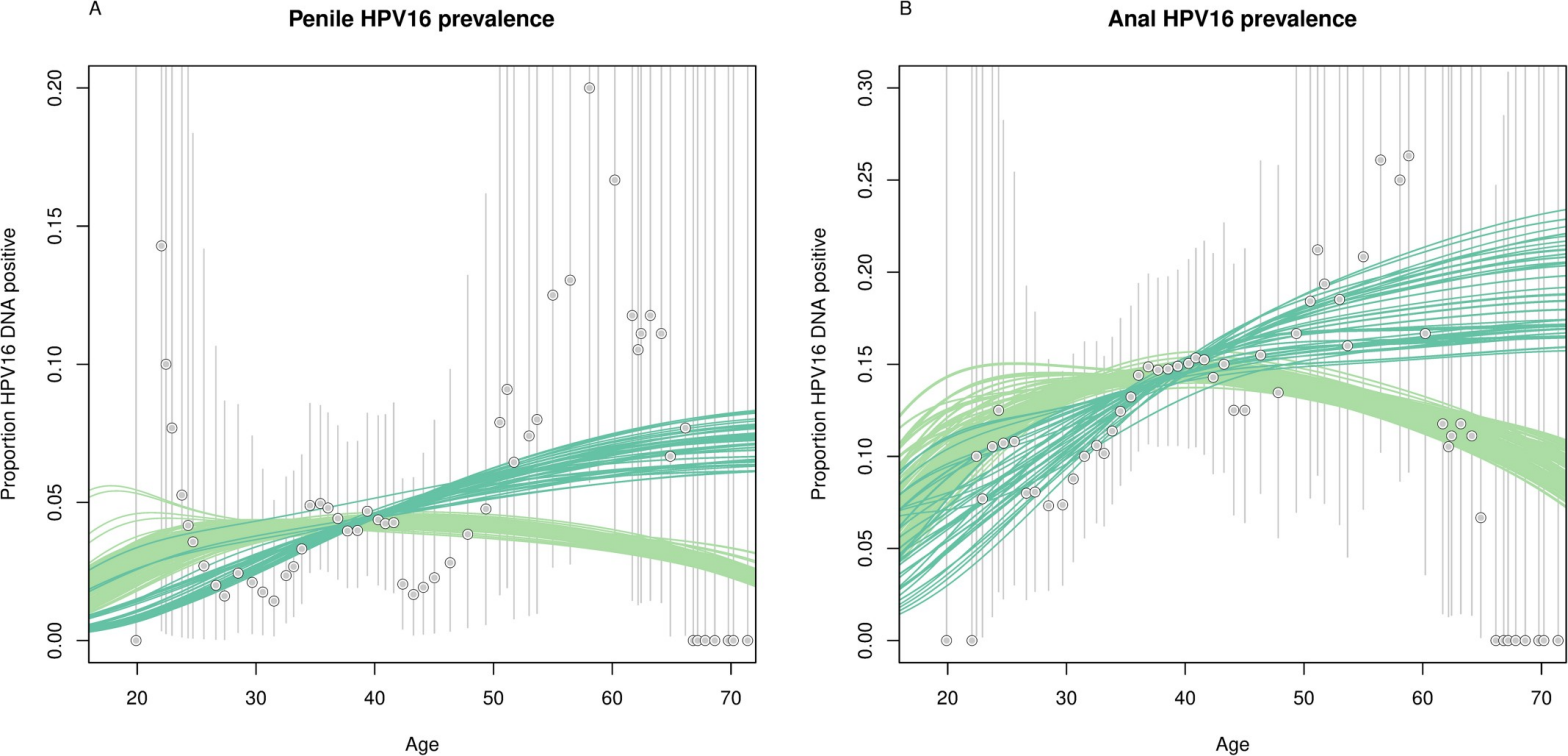
Prevalence of HPV16 infections among men who have sex with men. Age-specific predictions of (A) penile and (B) anal HPV16 infection are derived from 20 natural history models combined with 18 settings of sexual contact structure; predictions from models with latency (see Table 2) are shown in dark green, models without in light green. Observed age-specific proportions among HIV-negative H2M study participants (circles) are given as moving averages for 10-year age groups (11,21), (12,22), etc. Vertical lines reflect 95% binomial confidence intervals for the observed proportions. HPV16, human papillomavirus genotype 16.

The relative quality of each model with respect to H2M study data was more dependent on the assumed natural history of HPV16 infection than on the sexual contact structure of the model (S8 Fig). In models that allowed for reactivation of latent infections, HPV16 prevalence mostly increased with increasing age, whereas HPV16 prevalence peaked around 40 years in models without latency (Fig 2). Likewise, models without latency predicted most sexually active HPV16-positive MSM to be in their 30s, whereas models with latency predicted this group to be somewhat older (S10 Fig). The model-averaged prevalence in the total MSM population prior to vaccination was 3.9% (90% PI 3.8%–4.1%) for penile HPV16 infection and 12.6% (90% PI 12.1%–13.1%) for anal HPV16 infection.

**Fig 3:**
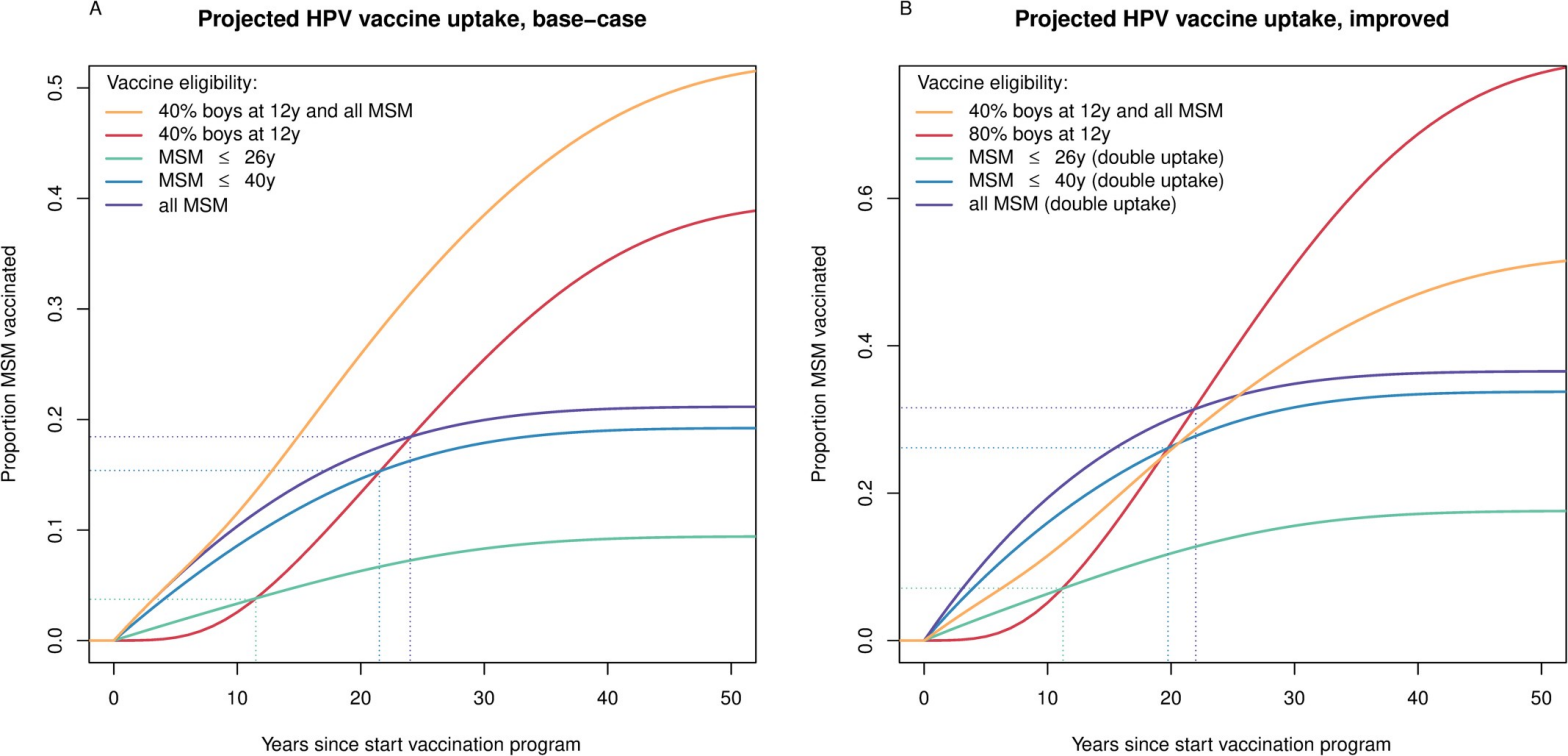
Projected coverage of HPV vaccination in MSM. The proportion of MSM vaccinated when assuming (A) similar uptake (base-case) or (B) 2-fold increased uptake (improved) as realized for hepatitis B vaccine among MSM, contrasted to the proportion of MSM vaccinated given sex-neutral vaccination in preadolescence, assuming (A) 40% or (B) 80% uptake among 12-year-old boys. In both panels, the combined strategy of vaccination at the age of 12 years with 40% uptake together with a targeted campaign among all MSM with base-case uptake is shown for comparison. HPV, human papillomavirus; MSM, men who have sex with men.

Offering vaccine to MSM aged ≤26 years achieved 9.4% vaccine coverage among MSM at the post-vaccination equilibrium in the base-case analysis (Table 3). This figure improved to 19.2% by extending vaccine eligibility to 40 years, and to 21.2% if the upper age for vaccine eligibility was discarded. Overall, the vaccine coverage among MSM achieved by targeted vaccination surpassed that of preadolescent boys’ vaccination in the first 12, 22, and 24 years of vaccination when offered to ≤26-year-old, ≤40-year-old, and all MSM, respectively, assuming similar HPV vaccine acceptance to that of HepB vaccine among MSM (Fig 3A). The combined strategy was projected to achieve 52.3% vaccine coverage among MSM. Adopting 2-fold increased uptake rates led to 17.6% vaccine coverage when vaccination was offered to MSM aged ≤26 years, 33.8% when offered until 40 years of age, and 36.5% if there was no age restriction. With doubled uptake, the vaccine coverage among MSM achieved by targeted vaccination surpassed that of preadolescent boys’ vaccination in the first 11, 20, and 22 years after initiating the vaccination strategy, respectively (Fig 3B).

**Table 3:** Projected reductions in anogenital HPV16 infection prevalence among MSM from prophylactic vaccination.

Strategy	Uptake^±^	Efficacy^¶^	Vaccine coverage among MSM		Penile HPV16 prevalence reduction*		Anal HPV16 prevalence reduction*	
			Ultimate	After 25 years	Ultimate^†^	After 25 years^††^	Ultimate^†^	After 25 years^††^
MSM ≤26 years	Base-case	85% all-or-nothing	9.4%	7.5%	14.3% (9.4%–18.8%)	10.7% (5.6%–14.5%)	13.4% (7.5%–17.8%)	9.9% (4.5%–13.7%)
	Improved	85% all-or-nothing	17.6%	14.0%	26.2% (17.4%–34.4%)	19.6% (10.4%–26.5%)	24.6% (14.1%–32.3%)	18.2% (8.5%–25.1%)
	Base-case	85% leaky if susceptible^§^	9.4%	7.5%	5.9% (2.5%–9.9%)	4.3% (1.7%–6.7%)	6.1% (2.6%–9.5%)	4.4% (2.0%–7.0%)
MSM ≤40 years	Base-case	85% all-or-nothing	19.2%	16.6%	27.2% (15.3%–37.2%)	22.6% (10.2%–31.5%)	25.5% (11.6%–34.8%)	20.9% (7.6%–29.7%)
	Improved	85% all-or-nothing	33.8%	29.6%	46.2% (27.4%–63.0%)	38.8% (18.2%–53.1%)	43.6% (21.4%–58.7%)	36.2% (13.8%–50.7%)
	Base-case	85% leaky if susceptible^§^	19.2%	16.6%	10.4% (3.9%–16.9%)	8.2% (3.0%–13.5%)	11.0% (4.8%–17.4%)	8.7% (4.0%–14.0%)
All MSM	Base-case	85% all-or-nothing	21.2%	18.8%	29.2% (15.9%–40.2%)	24.8% (10.7%–34.7%)	27.3% (11.9%–37.5%)	23.0% (7.9%–32.8%)
	Improved	85% all-or-nothing	36.5%	33.1%	48.8% (28.1%–66.9%)	42.2% (19.0%–57.8%)	46.1% (21.8%–62.4%)	39.4% (14.3%–55.4%)
	Base-case	85% leaky if susceptible^§^	21.2%	18.8%	11.1% (4.2%–18.1%)	8.9% (3.2%–15.0%)	11.7% (5.1%–18.6%)	9.5% (4.3%–15.4%)
Preadolescent boys	40% at 12 y	95% all-or-nothing	39.7%	19.6%	64.1% (53.2%–79.7%)	20.0% (9.6%–25.9%)	61.6% (48.4%–75.7%)	19.0% (10.8%–24.4%)
	80% at 12 y	95% all-or-nothing	79.3%	39.5%	97.9% (92.3%–99.9%)	37.7% (18.0%–48.0%)	97.4% (89.5%–99.9%)	36.0% (20.9%–45.4%)
	40% at 12 y	95% leaky	39.7%	19.6%	60.9% (45.4%–78.9%)	18.8% (9.4%–25.0%)	57.9% (38.2%–74.7%)	17.4% (10.0%–23.5%)
Preadolescent boys + all MSM	40% at 12 y + base-case	95% (at 12 y) + 85% all-or-nothing	52.3%	32.8%	77.1% (64.9%–95.2%)	46.5% (23.7%–59.6%)	74.8% (59.8%–93.0%)	44.2% (23.6%–57.2%)

^±^“Base-case” assumes similar age-specific uptake for selective MSM vaccination as realized for HepB vaccine; “improved” assumes doubled uptake as compared to HepB vaccine among MSM.

^¶^”All-or-nothing” assumes *x*% become fully protected and (100 − *x*)% remain fully susceptible; “leaky” assumes uniform *x*% infection hazard reductions.

*Percentage reduction in prevalence of infection at time *t* with vaccination, compared to prevalence of infection prior to introduction of vaccination.

^†^Model-averaged maximum (90% prediction interval) achieved in the post-vaccine epidemiologic equilibrium.

^††^Model-average (90% prediction interval) achieved after 25 years of vaccination strategy implementation.

^§^Susceptible for both penile and anal HPV16 infection.

HepB, hepatitis B; HPV16, human papillomavirus genotype 16; MSM, men who have sex with men.

**Fig 4:**
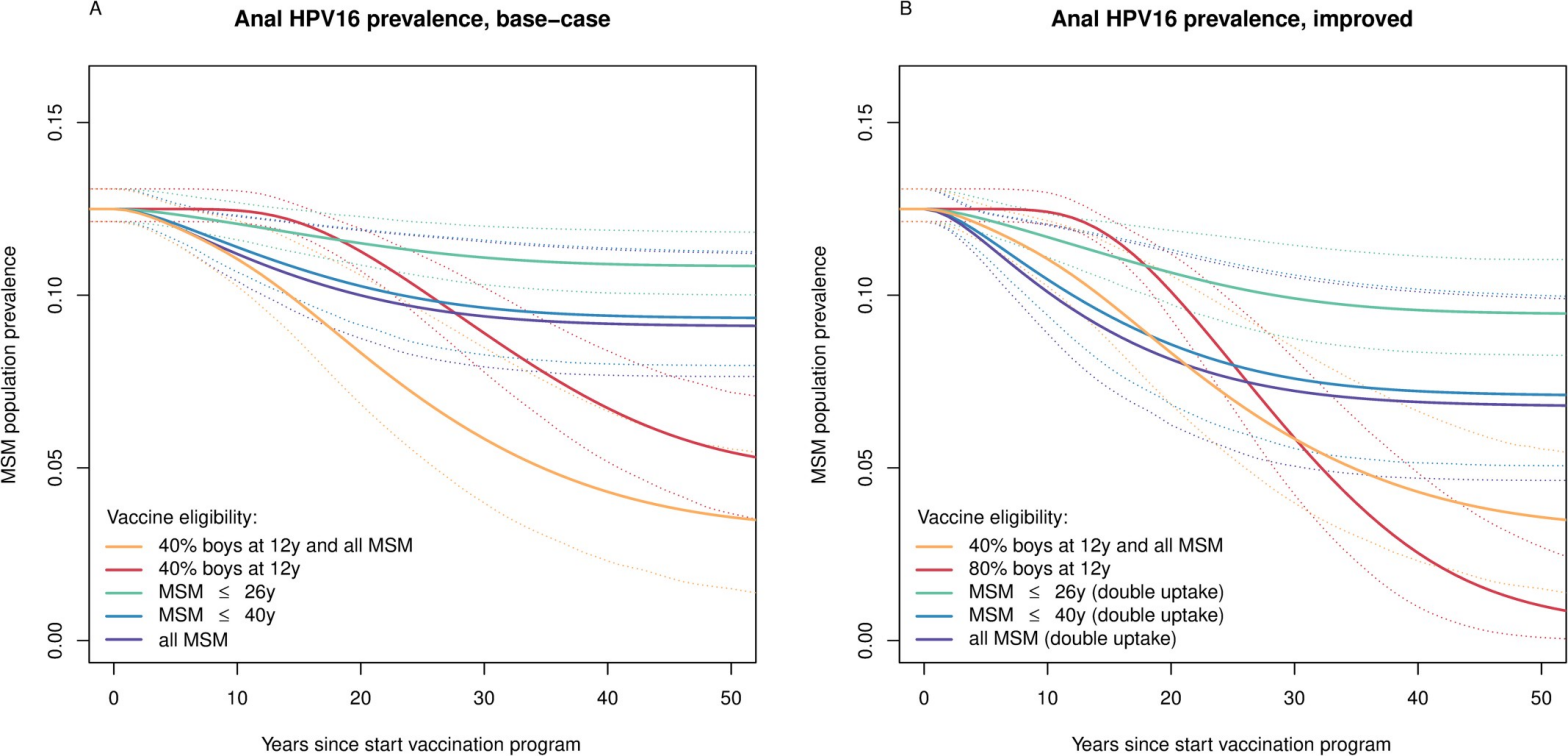
Projected impact of HPV vaccination in MSM. The model-averaged population prevalence of anal HPV16 infection among MSM by vaccination scenario with 90% prediction intervals (dotted lines), under (A) base-case and (B) improved HPV vaccine uptake in targeted vaccination. In both panels, the combined strategy of vaccination at the age of 12 years with 40% uptake together with a targeted campaign among all MSM with base-case uptake is shown for comparison. HPV, human papillomavirus; HPV16, human papillomavirus genotype 16; MSM, men who have sex with men.

With base-case uptake, offering vaccine to MSM aged ≤26 years resulted in a model-averaged reduction of 14.3% (90% PI 9.4%–18.8%) in equilibrium penile HPV16 infection, and a 13.4% reduction (90% PI 7.5%–17.8%) in equilibrium anal HPV16 infection, compared to the pre-vaccine prevalence of anogenital HPV16 infections among MSM. The predicted reductions improved to 27.2% (90% PI 15.3%–37.2%) and 25.5% (90% PI 11.6%–34.8%) in penile and anal HPV16 infections, respectively, if vaccine eligibility was extended to 40 years, and to 29.2% (90% PI 15.9%–40.2%) and 27.3% (90% PI 11.9%–37.5%), respectively, without an upper age for eligibility. HPV16 prevalence reductions in the post-vaccination equilibrium exceeded vaccine coverage projections (S11 Fig), and most of these reductions were realized within the first 30 years of a targeted immunization campaign, during which they exceeded those induced by vaccinating 40% of 12-year-old boys (Fig 4A). Moreover, the reductions in anogenital HPV16 prevalence from preadolescent boys’ vaccination were largely confined to younger MSM as compared to the reductions achieved by targeted vaccination (S12 Fig). However, the declines induced by preadolescent boys’ vaccination were sustained for much longer, ultimately leading to a 64.1% (90% PI 53.2%–79.7%) reduction in penile HPV16 infection among MSM, and a 61.6% (90% PI 48.4%–75.7%) reduction in anal HPV16 infection (S13 Fig). The combined strategy resulted in a 77.1% (90% PI 64.9%–95.2%) reduction in penile HPV16 infection and a 74.8% (90% PI 59.8%–93.0%) reduction in anal HPV16 infection among MSM (Table 3). Post-vaccination equilibria were achieved after 40 years in the case of targeted vaccination, but only after 60 years in scenarios involving preadolescent boys’ vaccination (S13–S15 Figs).

With doubled uptake, offering vaccine to MSM aged ≤26 years resulted in a model-averaged reduction of 26.2% (90% PI 17.4%–34.4%) in penile HPV16 infection and a 24.6% reduction (90% PI 14.1%–32.3%) in anal HPV16 infection. The corresponding reductions amounted to 46.2% (90% PI 27.4%–63.0%) and 43.6% (90% PI 21.4%–58.7%) in penile and anal HPV16 infections, respectively, if vaccine eligibility was extended to 40 years, and to 48.8% (90% PI 28.1%–66.9%) and 46.1% (90% PI 21.8%–62.4%), respectively, if no upper age for eligibility was considered. The reductions in anogenital HPV16 prevalence were sustained for over 40 years (S14 Fig), again exceeding vaccine coverage projections in the post-vaccination equilibrium (S11 Fig). Assuming 80% vaccine uptake among preadolescent boys resulted in the near elimination of anogenital HPV16 infections among MSM, with ≥90% reductions in anal HPV16 infection in 95% of model projections (Table 3). However, for the first 30 years of the vaccination strategies, the reductions induced by 80% preadolescent boys’ vaccination were smaller than those induced by vaccinating 40% of preadolescent boys in combination with offering selective vaccination to MSM without age restrictions, with base-case uptake (Fig 4B).

Targeted vaccination was only marginally effective if prophylactic efficacy was restricted to those fully susceptible at the time of immunization, while assuming similar uptake as realized for HepB vaccine. In this conservative scenario, offering vaccine to ≤26-year-old, ≤40-year-old, or all MSM resulted in penile HPV16 prevalence reductions of only 5.9% (90% PI 2.5%–9.9%), 10.4% (90% PI 3.9%–16.9%), and 11.1% (90% PI 4.2%–18.1%), respectively (Table 3). The corresponding reductions in anal HPV16 prevalence were 6.1% (90% PI 2.6%–9.5%), 11.0% (90% PI 4.8%–17.4%), and 11.7% (90% PI 5.1%–18.6%), respectively (S15 Fig). In these scenarios, HPV16 prevalence reductions remained below vaccine coverage projections (S11 Fig). The reductions induced by preadolescent boys’ vaccination were more robust, being only marginally reduced when vaccination provided “leaky” protection against infection.

**Fig 5:**
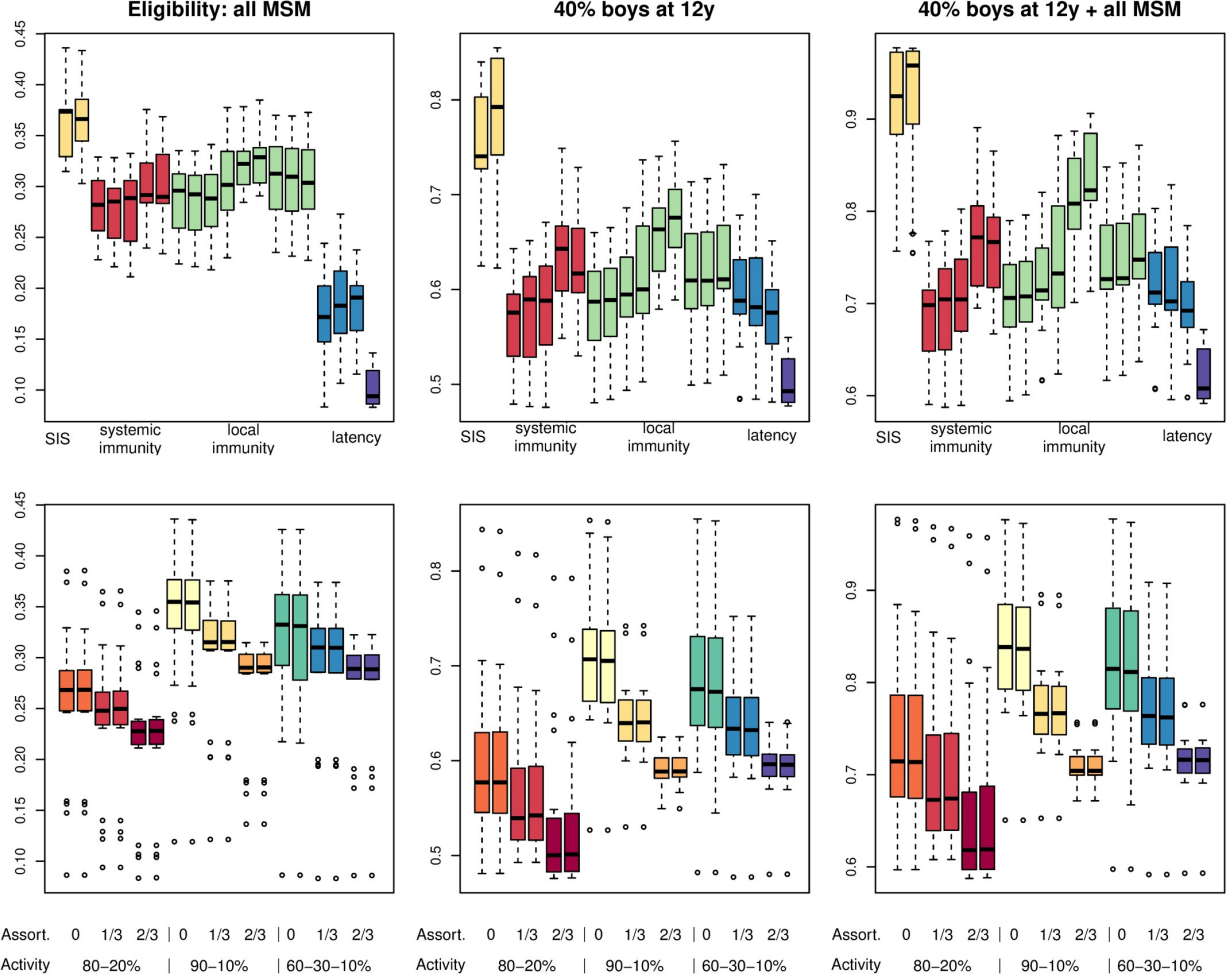
Effect of modeling assumptions on projected impact of vaccination. The base-case estimates of ultimate reductions in anal HPV16 infection prevalence among MSM by vaccination scenario. Data are summarized as Tukey boxplots (i.e., interquartile range [IQR], with whiskers extending to at most 1.5 times IQR from the box, and outliers shown separately) according to presumed natural history (upper panels; models ordered as in Table 2) and sexual contact structure (lower panels) of the models included in predictions. The various sexual contact structures are grouped according to sexual activity distribution, assortative mixing with respect to sexual activity, and assortative mixing with respect to preference for insertive/receptive anal sex. Note the differences in scale for the vaccination scenarios. HPV16, human papillomavirus genotype 16; MSM, men who have sex with men; SIS, susceptible-infected-susceptible model.

Models with neither natural immunity nor latency predicted the highest reductions in anogenital HPV16 infections among MSM, whereas models with latency predicted the lowest reductions from a targeted immunization campaign (Figs 5 and S16). Likewise, the effectiveness of preadolescent boys’ vaccination in reducing HPV16 prevalence among MSM was weakest in models that assumed latency in combination with natural immunity. These differences were maintained under improved vaccine uptake, but reductions from targeted immunization became less dependent on latency assumptions when prophylactic efficacy was restricted to those fully susceptible (S17 Fig). Estimated reductions in HPV16 prevalence among MSM increased with sexual contact heterogeneity and decreased with assortative mixing, irrespective of vaccination scenario (Fig 5). The findings were similar with improved vaccine uptake and with restricted efficacy (S17 Fig).

## Discussion

This study explored the potential effectiveness of HPV vaccination for MSM in the Netherlands. Based on predictions from a range of penile–anal HPV16 transmission models, we estimated that around 30% of anogenital infections might be prevented after 40 years if uptake similar to that of HepB vaccine among MSM throughout the Netherlands were realized. This figure increased to 75% after 60 years when targeted vaccination was combined with sex-neutral vaccination in preadolescence, assuming 40% uptake among 12-year-old boys. HPV16 prevalence reductions among MSM mostly exceeded vaccine coverage projections, illustrating the efficiency of prophylactic immunization even when HPV vaccine is given after sexual debut.

Our work suggests that HPV vaccination could be effective when delivered to MSM utilizing the infrastructure available for targeted HepB vaccination. Our analysis also shows that, while vaccinating young MSM is important, inclusion of older MSM is likely needed to achieve substantial vaccine coverage and impact. The predicted reductions improved to around 50% with doubled vaccination uptake rates and no upper age for eligibility. This scenario represents a vaccine coverage projection that resembles the estimated HepB vaccination coverage in Amsterdam [26], where most MSM were actively recruited from specialist sexual health services and outreach locations such as saunas and gay bars. The scope for improved prevention is thus considerable, offering key opportunities for raising awareness about HPV-related cancer and promoting HPV vaccine acceptance among MSM [27,28].

Several caveats should be taken into consideration when assessing the potential impact of HPV vaccination of MSM. First, our work indicates that it may take several decades before reductions in HPV infection level are fully achieved, and another 15–30 years before the full impact on cancer incidence is reached [29]. The models may have overestimated the time scale at which the effects of vaccination become apparent if assortative mixing with respect to age is strong. However, data suggest that for MSM partnerships, age-assortative mixing is much less present than for heterosexual partnerships [30–34]. The modest and slow reductions in HPV16 prevalence are partly due to the assumption that uptake of HPV vaccine among MSM would resemble that of HepB vaccine, where it took more than a decade before the effects on transmission and incidence could be demonstrated [26]. In addition, HPV16 is characterized by a relatively high reproduction potential as compared to other vaccine-protected HPV types [35]. Consequently, one should expect modest herd effects from vaccinating against HPV16 in comparison to other HPV types, as demonstrated in a community-randomized HPV vaccine trial [36]. An Australian study also predicted a long duration for targeted vaccination effects to become fully apparent in vaccine-type HPV prevalence among MSM [14]. This long duration may influence willingness to participate in a selective vaccination program, and will also negatively affect the cost-effectiveness profile of targeted vaccination. Both issues may be alleviated by vaccine inclusion of low-risk HPV types associated with anogenital warts, as vaccination has been shown to induce rapid declines in wart incidence [6,7]. Presumably, the favorable cost-effectiveness profile of selectively vaccinating MSM in the UK was driven by the inclusion of anogenital wart prevention, as the favorable profile only applied to the use of quadrivalent HPV vaccine (including low-risk types 6 and 11), and not to HPV16/18 vaccination [13].

Second, the validity of the base-case scenarios strongly depends on the assumption of 85% efficacy against future HPV16 infections, irrespective of HPV16 infection status at the time of immunization. Thus, we assumed that prophylactic efficacy would also apply to recipients already infected with (or immune to) HPV16 at the time of immunization. While conceivable, this assumption has yet to be tested empirically. The effectiveness of targeted vaccination is profoundly reduced if prophylactic efficacy applies only when vaccine recipients are fully susceptible at the time of immunization, as in per-protocol analyses of vaccine trials [15,16]. Yet, HPV vaccine has demonstrated high efficacy and immunogenicity in adult women 24–45 years of age, regardless of previous exposure to HPV vaccine type [37], likely making this latter scenario overly conservative.

Third, the effectiveness of targeted vaccination varied considerably between the models included in the analysis, with lower reductions predicted in models that assumed naturally acquired immunity or reactivation of latent infections. As the mechanisms of immunity and latency become better understood [38,39], models will need to be revised to adequately capture the interactions of vaccine-induced protection and naturally acquired immunity or latency. Likewise, more data on the occurrence, acquisition, and duration of anogenital HPV infections in MSM would help to narrow down the range of transmission models compatible with data, and increase the precision of model-averaged prediction. In the meantime, well-calibrated dynamic models should be equipped to simulate and explore various assumptions around age specificity in HPV16 infection prevalence. A multi-modeling approach [24], as employed in this analysis, is valuable when evaluating the impact of an intervention in light of many structural model uncertainties.

Our dynamic model is the first to our knowledge to explicitly incorporate site-specific HPV infection and transmission among MSM. Previous models of HPV transmission in MSM remained ambiguous about the routes of transmission being considered, and circumvented the need to explicate site-specific transmissibility by considering general transmission probabilities in same-sex partnerships [13,14]. While such an approach greatly reduces model complexity, it goes at the expense of essential detail as the risks of penile and anal HPV infections are mediated by different behaviors (i.e., insertive versus receptive anal intercourse). Moreover, it has been suggested that probabilities of HPV transmission from the penis to the anus are likely to be significantly higher than those from the anus to the penis [40]. This supposition is borne out by our analysis. The overall reproduction number of penile–anal transmission is a composite of both site-specific reproduction numbers, analogous to transmission from men to women and back to men [41]. Therefore, leaving site-specific transmissibility unspecified could lead to biased predictions about the prevention of anogenital HPV16 infections in MSM through prophylactic immunization.

We did not consider condom use in our HPV16 transmission models. While some studies have found that consistent condom use may provide some degree of protection against HPV infection among high-risk men [42], systematic reviews provide no consistent evidence that condom use reduces the risk of becoming HPV DNA-positive [43,44]. Likewise, we found no clear relation in the H2M study between condom use during anal sex in the preceding 6 months and transition from uninfected to infected HPV16 or HPV18 states [45]. Nevertheless, our estimates of site-specific transmissibility could be biased as they are not adjusted for condom use. Moreover, our projections are sensitive to changes in sexual risk behavior, including the possibility of decreased condom use, e.g., in response to the introduction of HIV pre-exposure prophylaxis in the Netherlands [46]. Indirect effects on HPV transmission due to factors unrelated to HPV vaccination were beyond the scope of this study, but should be considered before planning selective vaccination of MSM.

We also did not incorporate transmission to the penile or anal site via oral sex. A recent study concluded that traditional heterosexual HPV transmission models (concerned with cervicovaginal and penile infections) may underestimate the population-level effectiveness of vaccination if a high proportion of genital infections originate from extragenital sites, whereas vaccination effectiveness may be overestimated if natural immunity to genital infections can occur following extragenital infections [47]. Currently, there is no strong evidence that oral infections are a reservoir for anogenital infections in MSM, or that clearance of oral infections can induce systemic immunity [48]. Moreover, the prevalence of oral HPV16 infection was below 2% in the HIV-negative H2M study participants, with a 5-fold lower incidence of oral HPV16 infection compared to anogenital HPV16 infection [49]. Hence, a substantial bias due to using only penile–anal transmission models to assess the potential effectiveness of HPV16 vaccination of MSM is not likely.

Finally, we did not incorporate the direct effect of HIV on HPV transmission dynamics. HIV is a strong and independent determinant of penile and anal HPV infections [3,20,49], but likely has a limited role in driving HPV transmission given the ubiquity of HPV infections among MSM in the Netherlands and the comparatively low prevalence of HIV. Nevertheless, HIV is one of the strongest risk factors for anal cancer, suggesting an important role of HIV in disease progression [50]. The policy in the UK of vaccinating HIV-positive MSM up to age 45 years against HPV [51], informed by a health economic study [13], is challenged by recent empirical data on the lack of efficacy in HIV-positive MSM aged 27 years or older [52]. Our modeling results apply primarily to HIV-negative MSM, for whom the HPV vaccine has shown prophylactic efficacy in preventing type-specific infections [16], genital warts [53], and lesion recurrence [54]. More research is needed on the role of HIV in HPV-induced neoplasia before more detailed predictions can be made with regard to anal cancer prevention. In any case, inclusion of MSM at high risk for HIV is paramount to achieve meaningful impact in selective vaccination programs.

The comparison of targeted vaccination with sex-neutral vaccination in preadolescence serves to illustrate the temporal benefit derived from a targeted immunization campaign among MSM. In addition, the comparison also serves as a benchmark to judge the potential effectiveness of targeted vaccination. The result, that vaccinating preadolescent boys would ultimately be more effective than offering HPV vaccine to MSM, partly depends on the supposedly moderate uptake of HPV vaccine among MSM in the Netherlands. However, even a targeted campaign that could reach a similar proportion of MSM as preadolescent vaccination would not be as effective, given the reduced efficacy of HPV vaccine when given after sexual debut. Therefore, the benefit of targeting interventions to MSM lies in a faster effectiveness of HPV vaccination regarding cancer prevention in males, and—possibly—in a lower number needed to vaccinate to prevent disease in males [9]. Besides, a sizeable proportion of the Dutch MSM population is not born in the Netherlands and may be missed by preadolescent vaccination.

Our work suggests that selective vaccination of MSM would be especially effective when combined with sex-neutral vaccination in preadolescence. Even a moderate uptake among preadolescent boys would already generate a substantially increased yield when combined with targeted vaccination, as it would render MSM immune against HPV16 upon entrance into the sexually active population. Conversely, a combined strategy could also safeguard against disappointing or unstable uptake in preadolescent vaccination programs, in the sense that it may be more feasible and sustainable to reach 40% of 12-year-old boys and MSM through a targeted campaign than it is to achieve 80% uptake among 12-year-old boys. While vaccinating 80% of preadolescent boys would be needed to achieve near elimination of HPV16 among MSM, our findings suggest stronger effectiveness from a combined strategy of vaccinating 40% of boys in preadolescence together with a targeted campaign among MSM for the first 30 years of vaccination.

In conclusion, this study suggests that a targeted immunization campaign among MSM in the Netherlands may generate considerable reductions in anogenital HPV16 infections in a high-risk population. Sex-neutral vaccination in preadolescence is likely needed to eliminate HPV-related diseases as a public health problem in men and women alike, but targeted vaccination deserves consideration, at least temporarily, to protect currently active MSM at high risk for anal cancer.

## Supporting information

S1 Fig Entrance into the population at risk for anogenital HPV16 infection. (EPS)

S2 Fig Age distribution of population at risk for anogenital HPV16 infection. (EPS)

S3 Fig Under-recruitment of MSM with recent sexual debut in the H2M study population. (EPS)

S4 Fig Acquisition of anal sex partners. (EPS)

S5 Fig Distribution according to preference for insertive/receptive anal sex. (EPS)

S6 Fig Sensitivity analyses on HPV16 infection clearance. (EPS)

S7 Fig Site-specific HPV16 transmissibility estimates. (EPS)

S8 Fig Relative quality of models used in prediction. (EPS)

S9 Fig Age-specific vaccine uptake among MSM. (EPS)

S10 Fig Age distribution of anogenital HPV16 infection prevalence. The age distribution of HPV16 infections among MSM is obtained as the age-specific proportion of (A) penile or (B) anal HPV16 infection (Fig 2) from the stable age distribution of the modeled at-risk population (S2 Fig). Predictions from models with latency are shown in dark green, and those from models without latency in light green.
(EPS)

S11 Fig Projected prevalence reductions against vaccine coverage among MSM. Percentage reductions in the prevalence of anal HPV16 infection in the post-vaccination equilibrium compared to pre-vaccination prevalence, plotted against HPV vaccine coverage among MSM. Colors relate to various vaccination strategies, as explained in the legend. Closed circles denote base-case scenarios regarding uptake and efficacy. Squares denote scenarios of improved uptake (80% in preadolescence, doubled uptake among MSM), and open circles denote scenarios of “leaky” efficacy restricted to those fully susceptible at immunization.
(EPS)

S12 Fig Projected impact on the age distribution of HPV16 infections. The age distribution of penile (upper) and anal (lower) HPV16 infections among MSM after 25 years of vaccination strategy implementation (blue lines). Age distributions prior to vaccination (green lines) are as in S10 Fig. Left panels show results of targeted vaccination with similar uptake as realized for HepB vaccine among MSM. Right panels show results of preadolescent boys’ vaccination with 40% uptake at age 12 years.
(EPS)

S13 Fig Projected impact of HPV vaccination in base-case analysis. The population prevalence of penile (upper) and anal (lower) HPV16 infections. Results are shown for targeted vaccination with different age-specific eligibilities (assuming similar uptake as realized for HepB vaccine among MSM and “all-or-nothing” efficacy irrespective of infection status at immunization), for vaccination of 12-year-old boys at 40% uptake annually, and for a combination thereof. Individual model projections are shown in grey; red lines denote model-averaged predictions with 90% PIs.
(TIFF)

S14 Fig Projected impact of HPV vaccination with improved uptake. The population prevalence of penile (upper) and anal (lower) HPV16 infections. Results are shown for targeted vaccination with different age-specific eligibilities—assuming doubled uptake as compared to HepB vaccine among MSM and “all-or-nothing” efficacy irrespective of infection status at immunization—and for vaccination of 12-year-old boys at 80% uptake annually. Individual model projections are shown in grey; red lines denote model-averaged predictions with 90% PIs. The combined strategy still assumed base base-case uptake and is given for comparison.
(TIFF)

S15 Fig Projected impact of HPV vaccination with restricted efficacy. The population prevalence of penile (upper) and anal (lower) HPV16 infections. Results are shown for targeted vaccination with different age-specific eligibilities—assuming similar uptake as realized for HepB vaccine among MSM and “leaky” efficacy restricted to those fully susceptible at immunization—and for vaccination of 12-year-old boys at 40% uptake. Individual model projections are shown in grey; red lines denote model-averaged predictions with 90% PIs.
(TIFF)

S16 Fig Effect of modeling assumptions on projected impact of vaccination. The base-case estimates of ultimate reductions in penile HPV16 infection prevalence among MSM by vaccination scenario. Data are summarized as Tukey boxplots (i.e., interquartile range [IQR], with whiskers extending to at most 1.5 times IQR from the box, and outliers shown separately) according to the presumed natural history (upper panel; models ordered as in Table 2) and sexual contact structure (lower panel) of the models included in predictions. The various sexual contact structures are grouped according to sexual activity distribution, assortative mixing with respect to sexual activity, and assortative mixing with respect to preference for insertive/receptive anal sex.
(EPS)

S17 Fig Effect of modeling assumptions on projected impact of vaccination in sensitivity analyses. The ultimate reductions in anal HPV16 infection prevalence among MSM by vaccination scenario, assuming doubled uptake as compared to HepB vaccine among MSM (left panels), 80% uptake among 12-year-old boys and “all-or-nothing” efficacy irrespective of infection status at immunization (middle panels), or “leaky” efficacy restricted to those fully susceptible at immunization and base-case uptake (right panels). Data are summarized (with similar colors) as in Fig 5. Note the differences in scale for the vaccination scenarios.
(EPS)

S1 Text Dynamic population model. (PDF)

S2 Text HPV16 transmission models. (PDF)

S3 Text Fit to H2M study data. (PDF)

S4 Text Model-averaged predictions. (PDF)

## Abbreviations

HepB: hepatitis B
HPV: human papillomavirus
HPV16: human papillomavirus genotype 16
MSM: men who have sex with men
PI: prediction interval
