# Supplementary figures and images for: Potential effectiveness of prophylactic HPV immunization for men who have sex with men in the Netherlands: A multi-model approach
Source: PLoS Med. 2019 Mar 4;16(3):e1002756. doi: 10.1371/journal.pmed.1002756 (PMC6398832; doi:10.1371/journal.pmed.1002756)

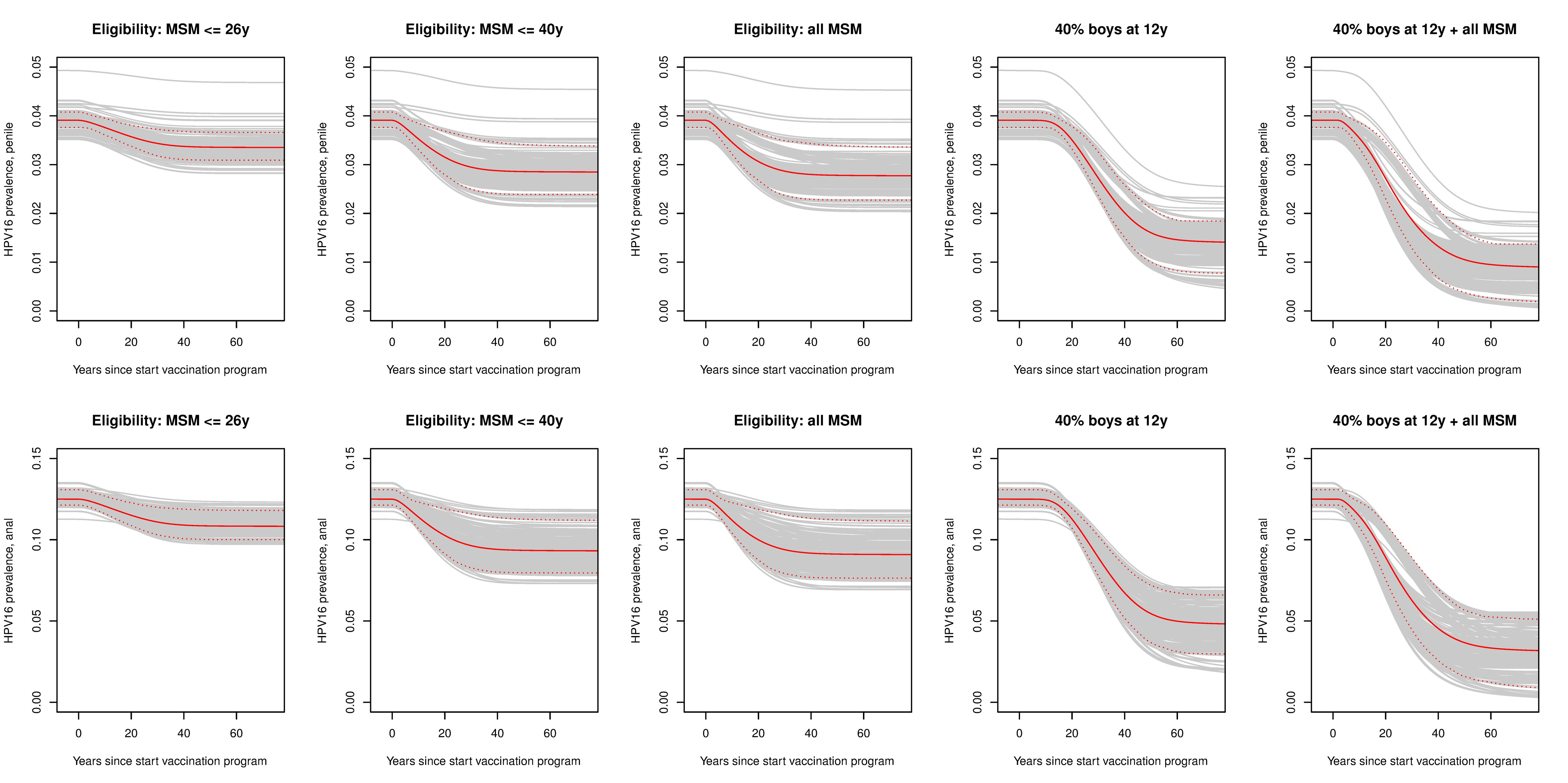

Supplement: S13 Fig — The population prevalence of penile (upper) and anal (lower) HPV16 infections. Results are shown for targeted vaccination with different age-specific eligibilities (assuming similar uptake as realized for HepB vaccine among MSM and “all-or-nothing” efficacy irrespective of infection status at immunization), for vaccination of 12-year-old boys at 40% uptake annually, and for a combination thereof. Individual model projections are shown in grey; red lines denote model-averaged predictions with 90% PIs. (TIFF) [file pmed.1002756.s013.tiff]

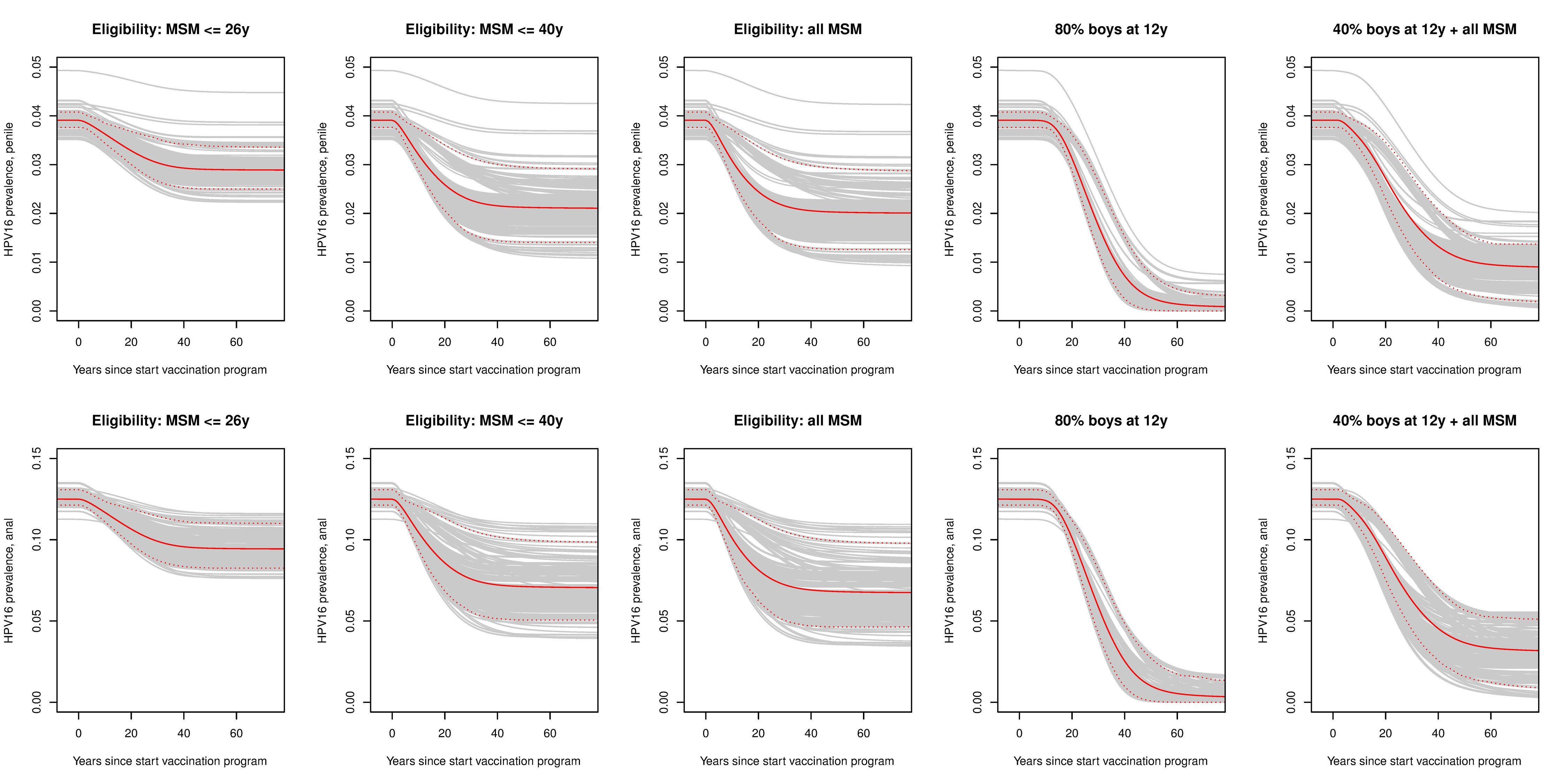

Supplement: S14 Fig — The population prevalence of penile (upper) and anal (lower) HPV16 infections. Results are shown for targeted vaccination with different age-specific eligibilities—assuming doubled uptake as compared to HepB vaccine among MSM and “all-or-nothing” efficacy irrespective of infection status at immunization—and for vaccination of 12-year-old boys at 80% uptake annually. Individual model projections are shown in grey; red lines denote model-averaged predictions with 90% PIs. The combined strategy still assumed base base-case uptake and is given for comparison. (TIFF) [file pmed.1002756.s014.tiff]

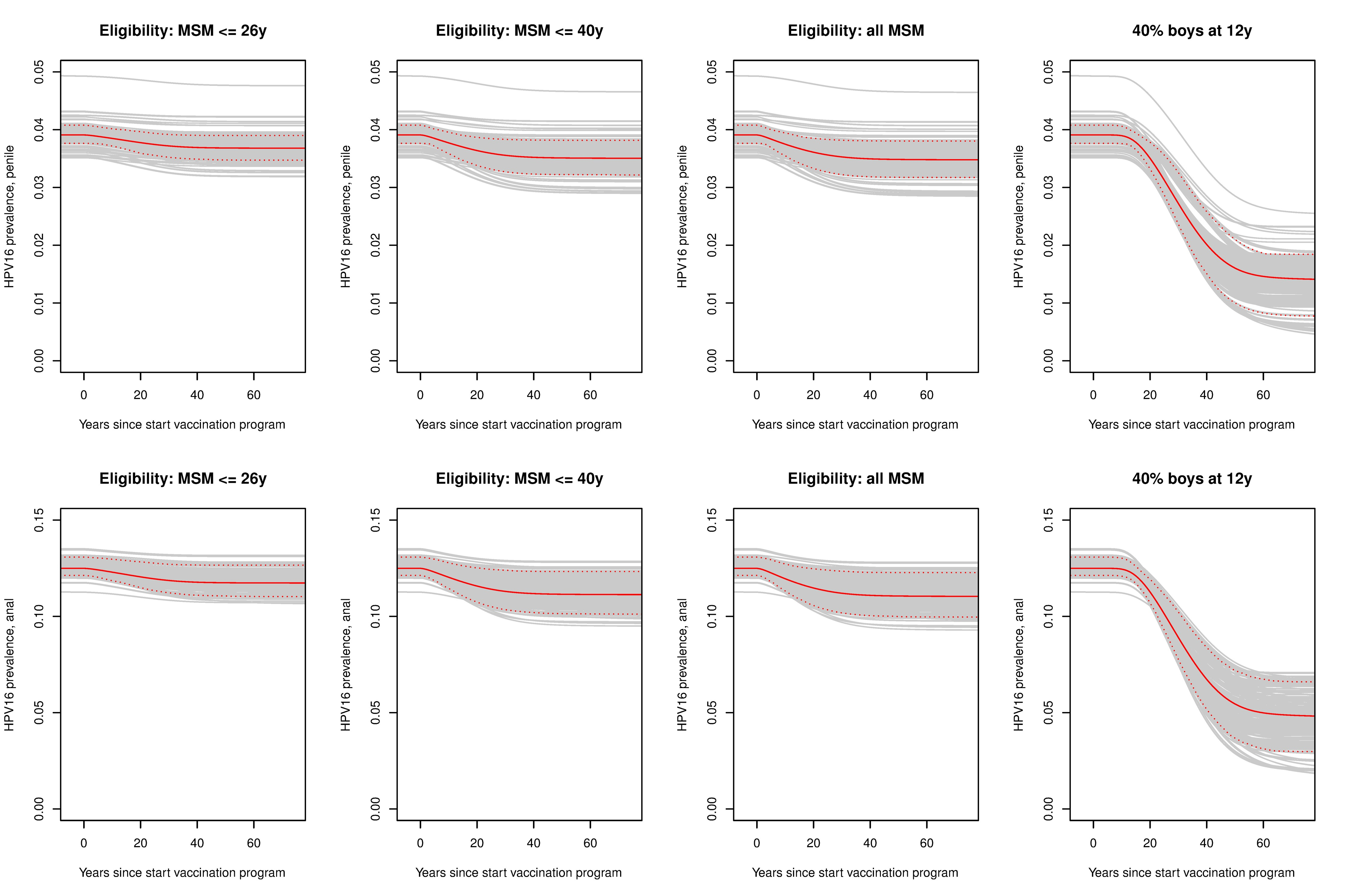

Supplement: S15 Fig — The population prevalence of penile (upper) and anal (lower) HPV16 infections. Results are shown for targeted vaccination with different age-specific eligibilities—assuming similar uptake as realized for HepB vaccine among MSM and “leaky” efficacy restricted to those fully susceptible at immunization—and for vaccination of 12-year-old boys at 40% uptake. Individual model projections are shown in grey; red lines denote model-averaged predictions with 90% PIs. (TIFF) [file pmed.1002756.s015.tiff]
