# Supplementary material for: Potential effectiveness of prophylactic HPV immunization for men who have sex with men in the Netherlands: A multi-model approach
Source: PLoS Med. 2019 Mar 4;16(3):e1002756. doi: 10.1371/journal.pmed.1002756 (PMC6398832; doi:10.1371/journal.pmed.1002756)
Supplement: S1 Text — (PDF) [file pmed.1002756.s018.pdf]

S1 TEXT: DYNAMIC POPULATION MODEL

PART I OF IV OF THE SUPPLEMENTARY ANNEX TO

POTENTIAL EFFECTIVENESS OF PROPHYLACTIC HPV IMMUNIZATION FOR MEN WHO HAVE SEX WITH MEN IN THE NETHERLANDS: A MULTI-MODEL APPROACH.

Johannes A Bogaards*, Sofie H Mooij, Maria Xiridou, Maarten F Schim van der Loeff

* Author to whom correspondence should be addressed: hans.bogaards@rivm.nl

TABLE OF CONTENTS

Supplement I: Dynamic population model *page 2*

S1.1 Partner acquisition *page 3*

S1.2 Sexual preference *page 6*

Supplementary References *page 12*

Legends to Supplementary Figures *page 13*

Supplement I: Dynamic population model

We constructed a dynamic population model of men who have sex with men (MSM) by estimating age-specific rates of entering and exiting the sexually active group of MSM at risk for new HPV16 infections. Entrance into the at-risk population was based on the self-reported age of first anal sex with a male partner, as disclosed by 705 out of 778 (>90%) of H2M study participants. All respondents reported first anal sex at an age above 10 years (median age: 21 years, 5th;95th percentile: 15;30 years), except one who reported first anal sex with a male partner at age 4 years, henceforth considered to be an outlier. Because the age distribution was skewed heavily to older age, we applied a logarithmic transformation before fitting a normal, lognormal and skew-normal distribution to the self-reported age of first anal sex. Distributions were compared by means of goodness-of-fit to the proportions with first anal sex at age ≤16, ≤20, ≤25, ≤30, ≤35, ≤40 years or thereafter. The skew-lognormal distribution provided comparatively best fit (p>0·05) and was selected for constructing the dynamic population model (S1 Fig). Because the selected probability density function (pdf) was defined on a logarithmic scale, with location, scale, and skewness parameters 2·85, 0·30, and 1·42, respectively, we applied the age-specific correction factor $(1/a)$ to ensure that the pdf summed to unity on the original age scale, i.e. $\int f(\log a)\mathrm{d}\log a = \int f(\log a)(1/a)\mathrm{d}a = 1$.

The rate of exiting the at-risk population, i.e. no longer acquiring new anal sex partners, was modelled by means of a Weibull hazard function, with shape, location, and scale parameters 3, 30, and 25, respectively. These values were chosen heuristically, to approximate the empirical density of MSM in the H2M study population from age 45 years onward. We note that the density of H2M study participants aged 30

to 45 years is markedly higher than predicted by the modelled rates of entering and exiting the at-risk population (S2 Fig), which is explained by under-recruitment of recently active MSM in the H2M study population (S3 Fig). With the selected parameterization, the median age in the modelled population was 37 years (5th;95th percentile: 20;58 years) and the expected duration of forming new same-sex male partnerships was 30·4 years. The latter can be calculated directly from the pdf's of entering and exiting the at-risk population with age-specific rates $\vartheta(a)$ and $\mu(a)$, because $\int_0^{a_\dagger} \vartheta(t_0) S(t_0)^{-1} \int_{t_0}^{a_\dagger} S(t) \mathrm{d}t \, \mathrm{d}t_0 = \int_0^{a_\dagger} \vartheta(t_0) \int_{t_0}^{a_\dagger} \exp\{-\int_{t_0}^{t} \mu(\tau) \mathrm{d}\tau\} \mathrm{d}t \, \mathrm{d}t_0$ [1], with $S(t) = \exp\{-\int_0^t \mu(\tau) \mathrm{d}\tau\}$.

S1.1 Partner acquisition

We modelled partner acquisition on the basis of self-reported numbers of anal sex partners in the last six months by HIV-negative H2M study participants, considered representative of the MSM population. HIV incidence rates in the Amsterdam cohort studies are comparable to HIV incidence rates among Amsterdam MSM as a whole, suggesting that HIV-negative study participants are at least representative of the local MSM community. To elaborate: the incidence of HIV infection in the cohort studies has stabilized at between 0.5 and 1 per 100 person-years [2]. With approximately 20,000 to 30,000 MSM living in Amsterdam, one would thus expect between 100 and 300 new HIV infections among MSM in Amsterdam. This compares well to the estimated annual HIV incidence of 700 new infections throughout the Netherlands, of which two-thirds occur among MSM with a concentration in Amsterdam [3].

Further corroboration of representativeness was obtained by comparing self-reported sexual behaviour of H2M study participants to the Schorer Monitor, a large-scale

internet survey investigating health, well-being, and sexuality among MSM throughout the Netherlands [4]. In 2010, this survey was completed by 3,602 MSM. Overall, HIV-negative H2M study participants reported comparable numbers of sex partners, except for MSM in their fifties who reported higher than expected numbers of recent anal sex partners as compared to the Schorer Monitor. This deviation possibly constitutes a local effect that we did not wish to capture in our model. Therefore, we conditioned the age-specificity in partner acquisition rate on the functional form observed in the Schorer Monitor, a parabolic function with roots at 0;80 years and maximum at 40 years. Parameters were obtained by fitting this function to the estimated number of newly acquired anal sex partners in the last six months in the H2M study population. To translate the self-reported number of anal sex partners into the number of newly acquired anal sex partners we again made use of the Schorer Monitor. In this monitor, 54% of respondents with one sex partner in the last six months reported sex with a steady partner, 37% with a casual partner, and 9% with an ex-partner. As 91% of steady partnerships reportedly lasted longer than six months, and 55% of ex-partnerships lasted shorter than six months, we inferred that for 54% x 91% + 9% x 45% = 53% of those reporting one sex partner in the last six months, it did not concern a new sex partner. Likewise, for 54% x 9% + 9% x 55% + 37% = 47% it concerned a new sex partner.

Of those reporting multiple sex partners in the last six months, 44% had sex with steady as well as casual partners; the remainder only reported casual sex. Only 2% reported multiple steady partners in the last six months. We projected these figures onto the H2M study population by assuming (i) that they were stable across different levels of sexual activity, (ii) that steady partners among those reporting multiple sex partners did not concern new partners, and (iii) that the proportion with multiple steady partners in the last six months could be neglected. The number of newly

acquired sex partners was subsequently obtained by subtracting 1 from the self-reported number of anal sex partners for a randomly selected subsample of 44% among those reporting multiple sex partners in the last six months. Results of this procedure are tabulated below:

Table A1. Projected numbers of newly acquired sex partners among HIV-negative H2M study participants

No. anal sex partners	Self-reported in last six months	Newly acquired (bootstrap estimate)
0	16% (n=73)	32% (n=147)
1	31% (n=140)	20% (n=90.2)
2–5	35% (n=160)	30% (n=139.3)
6–10	9% (n=43)	9% (n=40.1)
11–25	6% (n=29)	6% (n=28.4)
>25	3% (n=14)	3% (n=14)
Mean (std. dev.)	4.42 (8.31)	4.02 (8.29)

Finally, we fitted the presumed function of partner acquisition rate to the estimated number of newly acquired sex partners in the last six months by respondent age, assuming a log-normal distribution with age-specific mean $\bar{c}$ and variance $\bar{\bar{c}}$. Thus, partner acquisition peaked at a mean of 4·64 partners per half-year, with considerable variation (std. dev. 8·63). The resulting fit closely resembles the HIV-negative H2M study participants with 1% of extremes left out (S4 Fig).

To account for heterogeneous partner acquisition in the model population, we considered three configurations according to sexual activity. Neither of those is regarded as realistic, and their inclusion acts mainly to verify the robustness of our findings to alternative parameterizations. In the first configuration, 80% of MSM were set to have below-average activity $c(a) < \bar{c}(a)$ and 20% to have above-average activity $c(a) > \bar{c}(a)$. These figures were set to 90% and 10% in the second configuration. In the third configuration, 60% of MSM were set to have below-average activity, 30% to have average activity, and 10% to have above-average activity. All configurations were

conditioned on the age-specific mean and coefficient of variation (cv) in contact rates, motivated by the notion that the basic reproduction number of infection scales with both, i.e. $\mathcal{R}_0 \propto \bar{c} + \bar{\bar{c}}/\bar{c}$, in the absence of assortative (like-with-like) mixing [5]. Thus, regarding the settings for heterogeneous activity, we used the following configurations:

1. 80%—20% with maximum 0·65—43·8 sex partners per year
2. 90%—10% with maximum 3·53—61·1 sex partners per year
3. 60%—30%—10% with maximum 0·86—9·28—59·8 sex partners per year

All configurations yield a mean peak acquisition rate of 4·64 per half-year at age 40 years and cv of 1·68, assumed constant across the age range. The Gini indices of the three distributions are 0·74, 0·56 and 0·71, respectively, all in the range of numbers reported. Note, however, that both cv and Gini indices tended to increase with age, indicating increased heterogeneity in sexual behaviour at older age, an aspect that we did not capture in our dynamic model population:

Table A2. Number of anal sex partners in last six months with 1% of extremes left out

Age-group	<25 yrs	25-<35 yrs	35-<45 yrs	45-<55 yrs	55-<65 yrs	65+ yrs
n	13	141	235	41	18	8
mean	2·99	3·83	4·00	5·32	2·50	1·75
cv	1·22	1·53	1·58	1·77	1·54	1·95
Gini index	0·56	0·68	0·69	0·69	0·78	0·88

S1.2 Sexual preference

To account for penile-to-anal and anal-to-penile transmission, we incorporated sexual behaviour by distinguishing insertive anal intercourse from receptive anal intercourse [6,7]. The probabilities of engaging in only insertive, only receptive, or both insertive and receptive anal sex within a partnership were obtained from self-reported

activities with anal sex partners in the last six months. We assumed that each person reporting “only insertive anal sex” should be matched with a person reporting “only receptive anal sex” on a per-partnership basis. Thus, the per-partnership probability to engage in only insertive anal sex should be similar to that of only receptive anal sex. This assumption was borne out by the data: among the 198 MSM reporting only 1 anal sex partner during the last six months, 56 reported only insertive anal sex and 54 only receptive anal sex. Overall, 131 out of 644 MSM (20%) reported only insertive anal sex during the last six months versus 104 (16%) reporting only receptive anal sex ($p>0\cdot05$). Questionnaires did however not record the position during anal sex on a per-partnership basis, but only on an aggregate basis in the last six months. Therefore, we had to infer the probabilities of engaging in either insertive, either receptive, or both insertive and receptive anal sex within a partnership from aggregate observations, as follows.

Let p be the probability of having only insertive or receptive anal sex in a partnership. The likelihood for p given $x_1(n)$ MSM reporting only insertive anal sex with n partners, $x_2(n)$ reporting only receptive anal sex with n partners, and $x_{12}(n)$ reporting both insertive and receptive anal sex with n partners, is then defined as:

$$\mathcal{L}(p|x(n)) = \prod_{n=1}^{N}(p^n)^{x_1(n)}(p^n)^{x_2(n)}(1-2p^n)^{x_{12}(n)} \qquad \text{(Eq.1)}$$

However, the resulting maximum likelihood estimate does not provide a good fit to the data. This is likely due to the stringent assumption that every MSM would have equal probability to engage in insertive and/or receptive anal sex. Next, we assumed that probabilities of having insertive or receptive anal sex may differ between MSM. In other words, we assumed a mixture of individuals, some with high and others with low

tendency to engage in insertive or receptive anal sex. For simplicity, we assumed equal proportions q of individuals without tendency to engage in insertive anal sex or in receptive anal sex. Hence, the likelihood function becomes:

$$\mathcal{L}(p,q|x(n)) = \prod_{n=1}^{N}\{q+(1-2q)p^n\}^{x_1(n)+x_2(n)}\{1-2q-2(1-2q)p^n\}^{x_{12}(n)} \quad \text{(Eq.2)}$$

The maximum likelihood estimates of p and q were 0·20 and 0·13, respectively. The mixture model provided a remarkably good fit to the data (S5 Fig). Goodness-of-fit was assessed by computing the deviance relative to a saturated probability model, i.e. one that represents the data with 86 degrees of freedom. The deviance of the mixture model with only two parameters was 69·7 (p=0·87), so one might conclude that the mixture model accurately describes the data. However, the saturated model likely suffers from over-parametrization, so we also compared to a restricted model with 43 degrees of freedom, that assumes similar numbers of MSM reporting only insertive versus only receptive anal sex. The deviance of the mixture model relative to this restricted model was 40·4 (p=0·50). Again, the mixture model provides a satisfactory description of the data, so we adopted this for further constructing the dynamic population model.

With the selected parameterization, 13% of MSM have no tendency to engage in insertive anal sex; 13% have no tendency to engage in receptive anal sex; and the rest of MSM engage in insertive as well as receptive anal sex. Of the latter MSM, 20% ends up having only insertive anal sex, 20% only receptive anal sex, and 60% both insertive and receptive anal sex in one particular partnership. Mixing between these subgroups was modelled by a matrix, which defines the preference from “insertive” MSM, i.e. those with tendency to engage in insertive anal sex only (first row); “receptive” MSM, i.e. those with tendency to engage in receptive anal sex only (second row); and the

remainder, i.e. “versatile” MSM (third row), to the corresponding subgroups (columns), as follows:

$$\Phi = \begin{bmatrix} 0 & \phi_{12} & 1-\phi_{12} \\ \phi_{21} & 0 & 1-\phi_{21} \\ \phi_{31} & \phi_{32} & 1-\phi_{31}-\phi_{32} \end{bmatrix} \qquad \text{(Eq.3)}$$

Note that the demand for “insertive” MSM will be the number of “receptive” MSM with a preference for “insertive” MSM and the number “versatile” MSM with a preference for “insertive” MSM, times the contact rate. We assume throughout that “versatile” MSM adapt towards the preference of their partner, i.e. a partnership between a “versatile” and a “receptive” MSM will result in the first one having only insertive anal sex in this partnership. The demand should match the availability, which is q times the contact rate. Assuming contact rates are independent of preference for insertive/receptive anal sex, we can derive

$$\phi_{31} = \frac{q(1-\phi_{21})}{(1-2q)};\ \phi_{32} = \frac{q(1-\phi_{12})}{(1-2q)}.$$

Furthermore, to safeguard a balance in partnership formation, the demand from “insertive” MSM towards “receptive” MSM should be matched by the demand from “receptive” MSM towards “insertive” MSM. Hence, we have to assume $\phi_{21} = \phi_{12}$ and likewise $\phi_{31} = \phi_{32}$. The mixing matrix is thus determined by one free parameter, henceforth termed $\phi \equiv \phi_{21}$ and denoting the preference from “insertive” MSM towards “receptive” MSM and vice versa.

The overall proportion reporting only insertive or only receptive anal sex within a partnership was set at $q + (1-2q)p$, i.e. those with a strict preference plus a fraction of those with versatile preference for insertive or receptive anal sex. From this, we

obtained the conditional probability ρ of “versatile” MSM to have only insertive or receptive anal sex within a like-minded partnership as

$$\rho = \frac{p(1-2q)-q(1-\phi)}{(1-2q)-2q(1-\phi)}.$$

To obey the overall proportion reporting only insertive anal sex within a partnership, it is required that $p \geq q(1-\phi)(1-2q)^{-1}$, i.e. the fraction of “versatile” MSM with preference for “insertive” MSM must not exceed the overall proportion reporting only insertive or only receptive anal sex within a partnership.

In the extreme case that $\phi = 1$, the fraction of “versatile” MSM that will have only insertive or receptive anal sex within a like-minded partnership becomes similar to p. In this situation, the mixing matrix looks like

$$\Phi = \begin{bmatrix} 0 & 1 & 0 \\ 1 & 0 & 0 \\ 0 & 0 & 1 \end{bmatrix}.$$

This kind of mixing constitutes “assortative” mixing from the point of view of “versatile” MSM. The opposite situation that $\phi = 0$ arises when MSM with versatile preference mingle as much as possible with the other subgroups, in which case the mixing matrix looks like

$$\Phi = \begin{bmatrix} 0 & 0 & 1 \\ 0 & 0 & 1 \\ q(1-2q)^{-1} & q(1-2q)^{-1} & 1-2q(1-2q)^{-1} \end{bmatrix}.$$

Note that even in this situation of “disassortative” mixing, some “versatile” MSM may assume either an insertive or a receptive role within a like-minded partnership, i.e.

$\rho > 0$ if $p > q(1-2q)^{-1}$. To conclude, if "insertive" MSM have a random preference for "receptive" MSM and vice versa, the mixing matrix looks like

$$\Phi = \begin{bmatrix} 0 & q(1-q)^{-1} & 1-q(1-q)^{-1} \\ q(1-q)^{-1} & 0 & 1-q(1-q)^{-1} \\ q(1-q)^{-1} & q(1-q)^{-1} & 1-2q(1-q)^{-1} \end{bmatrix}.$$

The corresponding value for ρ is in between the values defined by assortative and disassortative mixing, but not necessarily in the middle of those extreme values. Given estimates of p and q of 0·20 and 0·13, respectively, the values for ϕ and ρ that correspond with "random" mixing are 0·15 and 0·072, respectively, whereas the minimum and maximum values for ρ are 0·0375 and 0·20, respectively.

SUPPLEMENTARY REFERENCES

1. Muldowney P, Ostaszewski K, Wojdowski W. The Darth Vader rule. Tatra Mt Math Publ. 2012;52:53–63.

2. Jansen IA, Geskus RB, Davidovich U, Jurriaans S, Coutinho RA, Prins M, et al. Ongoing HIV-1 transmission among men who have sex with men in Amsterdam: a 25-year prospective cohort study. AIDS. 2011;25(4):493-501.

3. van Sighem AI, Boender TS, Wit FWNM, Smit C, Matser A, Reiss P. Monitoring Report 2017. Human Immunodeficiency Virus (HIV) infection in the Netherlands. Amsterdam, the Netherlands: HIV Monitoring Foundation; 2017.

4. den Daas C, Goenee M, Bakker BH, de Graaf H, Op de Coul EL. Comparing databases: determinants of sexually transmitted infections, HIV diagnoses, and lack of HIV testing among men who have sex with men. BMC Public Health. 2015;15:1114.

5. Diekmann O, Heesterbeek JAP. Mathematical Epidemiology of Infectious Diseases: Model Building, Analysis and Interpretation (1st ed). Chichester: John Wiley; 2000.

6. Wiley JA, Herschkorn SJ. Homosexual role separation and AIDS epidemics: insights from elementary models. J Sex Res. 1989;26(4):434-49.

7. Coxon AP, Coxon NH, Weatherburn P, Hunt AJ, Hickson F, Davies PM, et al. Sex role separation in sexual diaries of homosexual men. AIDS. 1993;7(6):877-82.

LEGENDS TO SUPPLEMENTARY FIGURES

S1 Figure. Entrance into the population at risk for anogenital HPV16 infection. The empirical density (histogram in pink) was obtained from 705 H2M study participants. These data were aptly described (striped bars) by a skew-lognormal distribution (χ^2=12·3 on df=7; p=0·06).

S2 Figure. Age distribution of population at risk for anogenital HPV16 infection. The stable age distribution of the modelled at-risk population (red line) versus the empirical density of H2M study participants.

S3 Figure. Under-recruitment of MSM with recent sexual debut in the H2M study population. The under-recruitment of young and recently active MSM is apparent from the low density of participants with a young 'sexual age', i.e. calendar age at baseline minus age of first anal sex.

S4 Figure. Acquisition of anal sex partners. Numbers of anal sex partners reported in the last 6 months by H2M study participants at study baseline. Closed circles denote mean number of partners reported by HIV-negative study participants, with 95% confidence intervals (vertical dashed lines). The modelled contact rate (red line) closely resembles the mean number of partners reported by HIV-negative study participants in the past 6 months, with 1% of extremes left out.

S5 Figure. Distribution according to preference for insertive/receptive anal sex. The A) H2M questionnaire recorded whether study participants engaged in either active (i.e. insertive) and/or passive (i.e. receptive) anal sex during the past 6 months. The numbers observed agreed well with B) predictions by a mixture model for the tendency to engage in either insertive and/or receptive anal sex.
