# Supplementary material for: Potential effectiveness of prophylactic HPV immunization for men who have sex with men in the Netherlands: A multi-model approach
Source: PLoS Med. 2019 Mar 4;16(3):e1002756. doi: 10.1371/journal.pmed.1002756 (PMC6398832; doi:10.1371/journal.pmed.1002756)
Supplement: S2 Text — (PDF) [file pmed.1002756.s019.pdf]

S2 TEXT: HPV16 TRANSMISSION MODELS

PART II OF IV OF THE SUPPLEMENTARY ANNEX TO

POTENTIAL EFFECTIVENESS OF PROPHYLACTIC HPV IMMUNIZATION FOR MEN WHO HAVE SEX WITH MEN IN THE NETHERLANDS: A MULTI-MODEL APPROACH.

Johannes A Bogaards*, Sofie H Mooij, Maria Xiridou, Maarten F Schim van der Loeff

* Author to whom correspondence should be addressed: hans.bogaards@rivm.nl

TABLE OF CONTENTS

Supplement II: HPV16 transmission models *page 2*

S2.1 Systemic SI(S)RS models *page 5*

S2.2 Local SI(S)RS models *page 5*

S2.3 SISP(R)S models *page 7*

S2.4 SI(S)L models *page 8*

S2.5 SI(R)L models *page 10*

S2.6 SIS model upon vaccination *page 11*

Supplement II: HPV16 transmission models

The simplest model for penile-anal HPV16 transmission among MSM is of the susceptible-infectious-susceptible (SIS) form for both the penile and anal site, and includes the minimum of four compartments $\{SS, SI, IS, II\}$. In steady state prior to vaccination, the SIS model may be described by the following system of differential equations, with ∂_a denoting (partial) derivative by age:

$$\partial_a[SS]_i^k = \Theta_i^k(a) - \left([\lambda_{10}]_i^k + [\lambda_{01}]_i^k + [\lambda_{11}]_i^k\right)[SS]_i^k + \gamma_{10}[IS]_i^k + \gamma_{01}[SI]_i^k - \mu(a)[SS]_i^k$$
$$\partial_a[IS]_i^k = [\lambda_{10}]_i^k[SS]_i^k - [\lambda_{*1}]_i^k[IS]_i^k - \gamma_{10}[IS]_i^k + \gamma_{01}[II]_i^k - \mu(a)[IS]_i^k$$
$$\partial_a[SI]_i^k = [\lambda_{01}]_i^k[SS]_i^k - [\lambda_{1*}]_i^k[SI]_i^k - \gamma_{01}[SI]_i^k + \gamma_{10}[II]_i^k - \mu(a)[SI]_i^k$$
$$\partial_a[II]_i^k = [\lambda_{11}]_i^k[SS]_i^k + [\lambda_{*1}]_i^k[IS]_i^k + [\lambda_{1*}]_i^k[SI]_i^k - (\gamma_{10} + \gamma_{01})[II]_i^k - \mu(a)[II]_i^k \qquad \text{(Sys.1)}$$

For brevity, we drop dependence on age and time for the proportions in each of the four compartments. Individuals enter the sexually active population according to $\Theta_i^k(a) = \vartheta(a)\mathrm{A}^{-1}\mathbb{p}_k\mathbb{q}_i$ with $\vartheta(a)$ denoting the age-specific rates of entering the at-risk population, A the "life expectancy" within the at-risk population, $\mathbb{p}_k$ the fraction of individuals with sexual activity indexed by k, and $\mathbb{q}_i$ that of individuals with preference for insertive/receptive anal sex indexed by i. As such, it holds that $\int_0^{a_\dagger} N(a,t)\mathrm{d}a = 1 \;\forall\; t$ with $N(a,t) = \sum_k \sum_i \left([SS(a,t)]_i^k + [IS(a,t)]_i^k + [SI(a,t)]_i^k + [II(a,t)]_i^k\right)$. Subscripts i refer to preference for insertive/receptive anal sex, whereas the superscripts k refer to sexual activity.

Likewise, dependence on age and time is also dropped for the infection hazards $[\lambda_{10}(a,t)]_i^k$, $[\lambda_{01}(a,t)]_i^k$ and $[\lambda_{11}(a,t)]_i^k$, describing transmission to the penile site only,

to the anal site only, and to both sites from the same partner, respectively. The marginal infection hazards $[\lambda_{1*}]_i^k = [\lambda_{10}]_i^k + [\lambda_{11}]_i^k$ and $[\lambda_{*1}]_i^k = [\lambda_{01}]_i^k + [\lambda_{11}]_i^k$, denote transmission to either the penile or the anal site irrespective of the other. Penile and anal infections clear at a constant rates γ_{10} and γ_{01}, respectively, and individuals exit the at-risk population at an age-specific rate $\mu(a)$.

The hazards of infection are constructed as follows:

$$[\lambda_{..}(a,t)]_i^k = \int_0^{a_\dagger} [c(a)]^k \varrho(a,a',t) \sum_l \varphi_{kl}(a') \sum_j \phi_{ij} \, [\mathrm{P}_{..}(a',t)]_j^l \mathrm{d}a' \qquad \text{(Eq.4)}$$

Here, $[c(a)]^k$ denotes the age-specific partner acquisition rate for someone with sexual activity k, and $\varrho(a,a')$ the probability of someone aged a to form a sexual partnership with someone aged a'. Age dependence only applies to the potential infector if this probability is determined by age-specificity in contact rates:

$$\varrho(a,a',t) = \frac{\sum_m [c(a')]^m \sum_i [N(a',t)]_i^m}{\int_0^{a_\dagger} \sum_m [c(a'')]^m \sum_i [N(a'',t)]_i^m \mathrm{d}a''} \qquad \text{(Eq.5)}$$

The values ϕ_{ij} are taken from the mixing matrix between subgroups with particular preference for insertive/receptive anal sex, as described before. Mixing between subgroups with different levels of sexual activity is included by weighing for age-specific contact rates, and by allowing for a degree of assortativeness ϵ between partners with similar sexual activity, as:

$$\varphi_{kl}(a') = \epsilon \delta_{kl} + (1-\epsilon) \frac{[c(a')]^l \sum_i [N(a')]_i^l}{\sum_m [c(a')]^m \sum_i [N(a')]_i^m} \qquad \text{(Eq.6)}$$

Here, δ_{kl} is the Kronecker delta, i.e. $\delta_{kl} = 1$ if $k = l$ and $\delta_{kl} = 0$ otherwise, and $\epsilon = 1$ describes fully assortative mixing whereas $\epsilon = 0$ describes random mixing.

$[\mathrm{P}_{..}(a',t)]_j^l$ denotes the probability of transmission from a potential infector with sexual activity l and preference for insertive/receptive anal sex j, in relation to the infection hazard under consideration. Transmission to the penile site only is then given by

$$[\mathrm{P}_{10}(a',t)]_j^l = [\rho_{10}]_{ij}\frac{\beta_{1*}\left([SI(a',t)]_j^l+[II(a',t)]_j^l\right)}{\mathbb{p}_l\mathbb{q}_j N(a',t)} + [\rho_{11}]_{ij}\frac{\beta_{1*}\left([SI(a',t)]_j^l+(1-\beta_{*1})[II(a',t)]_j^l\right)}{\mathbb{p}_l\mathbb{q}_j N(a',t)} \; .$$

The values $[\rho_{10}]_{ij}$ and $[\rho_{10}]_{ij}$ denote the probability of engaging in only insertive anal sex or in insertive as well as receptive anal sex, when someone with preference for insertive/receptive anal sex i has a partner with preference j. Following previous definitions, $\rho_{10} = 1$ and $\rho_{11} = 0$ when either partner has tendency to engage in insertive anal sex only, whereas $\rho_{10} = \rho$ and $\rho_{11} = 1 - 2\rho$ when both partners have versatile preference. The probabilities β_{1*} and β_{*1} denote the per-partnership probabilities of anal-to-penile and penile-to-anal transmission, respectively. Similarly, transmission to the anal site only is given by

$$[\mathrm{P}_{01}(a',t)]_j^l = [\rho_{01}]_{ij}\frac{\beta_{*1}\left([IS(a',t)]_j^l+[II(a',t)]_j^l\right)}{\mathbb{p}_l\mathbb{q}_j N(a',t)} + [\rho_{11}]_{ij}\frac{\beta_{*1}\left([IS(a',t)]_j^l+(1-\beta_{1*})[II(a',t)]_j^l\right)}{\mathbb{p}_l\mathbb{q}_j N(a',t)} \; .$$

The values $[\rho_{01}]_{ij}$ denote the probability of engaging in only receptive anal sex when someone with preference for insertive/receptive anal sex i has a partner with preference j. Again, $\rho_{01} = \rho$ and $\rho_{11} = 1 - 2\rho$ when both partners have versatile preference, while $\rho_{01} = 1$ and $\rho_{11} = 0$ when either partner has tendency to engage in receptive anal sex only. The hazard of acquiring infection at both sites from a partner is only defined when both partners have versatile preference, and is given by

$$[\mathrm{P}_{11}(a',t)]_j^l = [\rho_{11}]_{ij}\frac{\beta_{1*}\beta_{*1}[II(a',t)]_j^l}{\mathbb{p}_l\mathbb{q}_j N(a',t)} \; .$$

S2.1 Systemic SI(S)RS models

If clearance of infections may lead to systemic immunity, that may subsequently be lost at a constant rate κ, the model becomes:

$$\partial_a [SS]_i^k = \Theta_i^k(a) - \left([\lambda_{10}]_i^k + [\lambda_{01}]_i^k + [\lambda_{11}]_i^k\right)[SS]_i^k + (1-\mathrm{frac})\gamma_{10}[IS]_i^k + (1-\mathrm{frac})\gamma_{01}[SI]_i^k + \kappa[RR]_i^k - \mu(a)[SS]_i^k$$

$$\partial_a [IS]_i^k = [\lambda_{10}]_i^k[SS]_i^k - [\lambda_{*1}]_i^k[IS]_i^k - \gamma_{10}[IS]_i^k + (1-\mathrm{frac})\gamma_{01}[II]_i^k - \mu(a)[IS]_i^k$$

$$\partial_a [SI]_i^k = [\lambda_{01}]_i^k[SS]_i^k - [\lambda_{1*}]_i^k[SI]_i^k - \gamma_{01}[SI]_i^k + (1-\mathrm{frac})\gamma_{10}[II]_i^k - \mu(a)[SI]_i^k$$

$$\partial_a [II]_i^k = [\lambda_{11}]_i^k[SS]_i^k + [\lambda_{*1}]_i^k[IS]_i^k + [\lambda_{1*}]_i^k[SI]_i^k - (\gamma_{10}+\gamma_{01})[II]_i^k - \mu(a)[II]_i^k$$

$$\partial_a [RR]_i^k = \mathrm{frac}\,\gamma_{10}[IS]_i^k + \mathrm{frac}\,\gamma_{01}[SI]_i^k + \mathrm{frac}\,(\gamma_{10}+\gamma_{01})[II]_i^k - \kappa[RR]_i^k - \mu(a)[RR]_i^k$$

(Sys.2)

Here, frac denotes the fraction developing immunity upon clearance of infection, which is taken to be the same for clearance of penile and anal infections. The SIRS, SIS33RS and SIS10RS models are obtained by setting this fraction to 1, 0·33 and 0·10, respectively. Infection hazards are modified by adjusting the probability that a sexual contact is infectious: $N(a,t) = \sum_k \sum_i \left([SS(a,t)]_i^k + [IS(a,t)]_i^k + [SI(a,t)]_i^k + [II(a,t)]_i^k + [RR(a,t)]_i^k\right)$.

S2.2 Local SI(S)RS models

If clearance of infections may lead to local immunity, that may subsequently be lost at a constant rate κ, the model becomes:

$$\partial_a [SS]_i^k = \Theta_i^k(a) - \left([\lambda_{10}]_i^k + [\lambda_{01}]_i^k + [\lambda_{11}]_i^k\right)[SS]_i^k + (1-\mathrm{frac}_{1*})\gamma_{10}[IS]_i^k + (1-\mathrm{frac}_{*1})\gamma_{01}[SI]_i^k + \kappa\left([RS]_i^k + [SR]_i^k\right) - \mu(a)[SS]_i^k$$

$$\partial_a[IS]_i^k = [\lambda_{10}]_i^k[SS]_i^k - [\lambda_{*1}]_i^k[IS]_i^k - \gamma_{10}[IS]_i^k + (1-\mathrm{frac}_{*1})\gamma_{01}[II]_i^k + \kappa[IR]_i^k - \mu(a)[IS]_i^k$$

$$\partial_a[SI]_i^k = [\lambda_{01}]_i^k[SS]_i^k - [\lambda_{1*}]_i^k[SI]_i^k - \gamma_{01}[SI]_i^k + (1-\mathrm{frac}_{1*})\gamma_{10}[II]_i^k + \kappa[RI]_i^k - \mu(a)[SI]_i^k$$

$$\partial_a[II]_i^k = [\lambda_{11}]_i^k[SS]_i^k + [\lambda_{*1}]_i^k[IS]_i^k + [\lambda_{1*}]_i^k[SI]_i^k - (\gamma_{10}+\gamma_{01})[II]_i^k - \mu(a)[II]_i^k$$

$$\partial_a[RS]_i^k = \mathrm{frac}_{1*}\,\gamma_{10}[IS]_i^k - [\lambda_{*1}]_i^k[RS]_i^k - \kappa[RS]_i^k + (1-\mathrm{frac}_{*1})\gamma_{01}[RI]_i^k + \kappa[RR]_i^k - \mu(a)[RS]_i^k$$

$$\partial_a[SR]_i^k = \mathrm{frac}_{*1}\,\gamma_{01}[SI]_i^k - [\lambda_{1*}]_i^k[SR]_i^k - \kappa[SR]_i^k + (1-\mathrm{frac}_{1*})\gamma_{10}[IR]_i^k + \kappa[RR]_i^k - \mu(a)[SR]_i^k$$

$$\partial_a[RI]_i^k = [\lambda_{*1}]_i^k[RS]_i^k + \mathrm{frac}_{1*}\,\gamma_{10}[II]_i^k - \gamma_{01}[RI]_i^k - \kappa[RI]_i^k - \mu(a)[RI]_i^k$$

$$\partial_a[IR]_i^k = [\lambda_{1*}]_i^k[SR]_i^k + \mathrm{frac}_{*1}\,\gamma_{01}[II]_i^k - \gamma_{10}[IR]_i^k - \kappa[IR]_i^k - \mu(a)[IR]_i^k$$

$$\partial_a[RR]_i^k = \mathrm{frac}_{*1}\,\gamma_{01}[RI]_i^k + \mathrm{frac}_{1*}\,\gamma_{10}[IR]_i^k - 2\kappa[RR]_i^k - \mu(a)[RR]_i^k \qquad \text{(Sys.3)}$$

Infection hazards are modified by considering all infectious compartments in the terms $[\mathrm{P}_{..}(a',t)]_j^l$ in relation to the infection hazard under consideration. For instance, transmission to the penile site only becomes dependent on

$$[\rho_{10}]_{ij}\frac{\beta_{1*}\left([SI(a',t)]_j^l+[II(a',t)]_j^l+[RI(a',t)]_j^l\right)}{\mathbb{p}_l\mathbb{q}_jN(a',t)} + [\rho_{11}]_{ij}\frac{\beta_{1*}\left([SI(a',t)]_j^l+(1-\beta_{*1})[II(a',t)]_j^l+[RI(a',t)]_j^l\right)}{\mathbb{p}_l\mathbb{q}_jN(a',t)}\,.$$

with $N(a,t)$ now summed over all compartments, and so on. Now, frac_{1*} and frac_{*1} denote the fractions developing local immunity upon clearance of either penile or anal infection, respectively. The SIR[local]S, SIS33R[local]S and SIS10R[local]S models are obtained by setting these fractions to 1, 0·33 and 0·10, respectively for both sites. Models with local immunity at the penile site only are obtained by varying frac_{1*} accordingly, while setting $\mathrm{frac}_{*1} = 0$. Likewise, models with local immunity at the anal site only are obtained by varying frac_{*1} and setting $\mathrm{frac}_{1*} = 0$.

S2.3 SISP(R)S models

The SIS model is extended to include persistent infections developing at a rate ζ_{10} and ζ_{01} and clearing at a rate $\xi_{10} < \gamma_{10}$ and $\xi_{01} < \gamma_{01}$ for penile and anal infections, respectively. If clearance does not induce immunity, such a SISPS model looks like:

$$\partial_a[SS]_i^k = \Theta_i^k(a) - \left([\lambda_{10}]_i^k + [\lambda_{01}]_i^k + [\lambda_{11}]_i^k\right)[SS]_i^k + \gamma_{10}[IS]_i^k + \gamma_{01}[SI]_i^k + \xi_{10}[PS]_i^k + \xi_{01}[SP]_i^k - \mu(a)[SS]_i^k$$

$$\partial_a[IS]_i^k = [\lambda_{10}]_i^k[SS]_i^k - [\lambda_{*1}]_i^k[IS]_i^k - (\gamma_{10} + \zeta_{10})[IS]_i^k + \gamma_{01}[II]_i^k - \mu(a)[IS]_i^k$$

$$\partial_a[SI]_i^k = [\lambda_{01}]_i^k[SS]_i^k - [\lambda_{1*}]_i^k[SI]_i^k - (\gamma_{01} + \zeta_{01})[SI]_i^k + \gamma_{10}[II]_i^k - \mu(a)[SI]_i^k$$

$$\partial_a[II]_i^k = [\lambda_{11}]_i^k[SS]_i^k + [\lambda_{*1}]_i^k[IS]_i^k + [\lambda_{1*}]_i^k[SI]_i^k - (\gamma_{10} + \gamma_{01} + \zeta_{10} + \zeta_{01})[II]_i^k - \mu(a)[II]_i^k$$

$$\partial_a[PS]_i^k = \zeta_{10}[IS]_i^k - [\lambda_{*1}]_i^k[PS]_i^k - \xi_{10}[PS]_i^k + \gamma_{01}[PI]_i^k + \xi_{01}[PP]_i^k - \mu(a)[PS]_i^k$$

$$\partial_a[SP]_i^k = \zeta_{01}[SI]_i^k - [\lambda_{1*}]_i^k[SP]_i^k - \xi_{01}[SP]_i^k + \gamma_{10}[IP]_i^k + \xi_{10}[PP]_i^k - \mu(a)[SP]_i^k$$

$$\partial_a[PI]_i^k = [\lambda_{*1}]_i^k[PS]_i^k + \zeta_{10}[II]_i^k - (\gamma_{01} + \zeta_{01} + \xi_{10})[PI]_i^k - \mu(a)[PI]_i^k$$

$$\partial_a[IP]_i^k = [\lambda_{1*}]_i^k[SP]_i^k + \zeta_{01}[II]_i^k - (\gamma_{10} + \zeta_{10} + \xi_{01})[IP]_i^k - \mu(a)[PI]_i^k$$

$$\partial_a[PP]_i^k = \zeta_{01}[PI]_i^k + \zeta_{10}[IP]_i^k - (\xi_{10} + \xi_{01})[PP]_i^k - \mu(a)[PP]_i^k \qquad \text{(Sys.4)}$$

Again, infection hazards are modified by considering all infectious compartments in the terms $[\mathrm{P}_{..}(a', t)]_j^l$ in relation to the infection hazard under consideration, and summing $N(a, t)$ over all compartments.

If it is assumed that clearance of persistent infections leads to systemic immunity, the model becomes:

$$\partial_a[SS]_i^k = \Theta_i^k(a) - \left([\lambda_{10}]_i^k + [\lambda_{01}]_i^k + [\lambda_{11}]_i^k\right)[SS]_i^k + \gamma_{10}[IS]_i^k + \gamma_{01}[SI]_i^k + \kappa[RR]_i^k - \mu(a)[SS]_i^k$$

$$\partial_a[IS]_i^k = [\lambda_{10}]_i^k[SS]_i^k - [\lambda_{*1}]_i^k[IS]_i^k - (\gamma_{10} + \zeta_{10})[IS]_i^k + \gamma_{01}[II]_i^k - \mu(a)[IS]_i^k$$

$$\partial_a[SI]_i^k = [\lambda_{01}]_i^k[SS]_i^k - [\lambda_{1*}]_i^k[SI]_i^k - (\gamma_{01} + \zeta_{01})[SI]_i^k + \gamma_{10}[II]_i^k - \mu(a)[SI]_i^k$$

$$\partial_a[II]_i^k = [\lambda_{11}]_i^k[SS]_i^k + [\lambda_{*1}]_i^k[IS]_i^k + [\lambda_{1*}]_i^k[SI]_i^k - (\gamma_{10} + \gamma_{01} + \zeta_{10} + \zeta_{01})[II]_i^k - \mu(a)[II]_i^k$$

$$\partial_a[PS]_i^k = \zeta_{10}[IS]_i^k - [\lambda_{*1}]_i^k[PS]_i^k - \xi_{10}[PS]_i^k + \gamma_{01}[PI]_i^k - \mu(a)[PS]_i^k$$

$$\partial_a[SP]_i^k = \zeta_{01}[SI]_i^k - [\lambda_{1*}]_i^k[SP]_i^k - \xi_{01}[SP]_i^k + \gamma_{10}[IP]_i^k - \mu(a)[SP]_i^k$$

$$\partial_a[PI]_i^k = [\lambda_{*1}]_i^k[PS]_i^k + \zeta_{10}[II]_i^k - (\gamma_{01} + \zeta_{01} + \xi_{10})[PI]_i^k - \mu(a)[PI]_i^k$$

$$\partial_a[IP]_i^k = [\lambda_{1*}]_i^k[SP]_i^k + \zeta_{01}[II]_i^k - (\gamma_{10} + \zeta_{10} + \xi_{01})[IP]_i^k - \mu(a)[PI]_i^k$$

$$\partial_a[PP]_i^k = \zeta_{01}[PI]_i^k + \zeta_{10}[IP]_i^k - \mathrm{g}(\xi_{10}, \xi_{01})\,[PP]_i^k - \mu(a)[PP]_i^k$$

$$\partial_a[RR]_i^k = \xi_{10}\left([PS]_i^k + [PI]_i^k\right) + \xi_{01}\left([SP]_i^k + [IP]_i^k\right) + \mathrm{g}(\xi_{10}, \xi_{01})\,[PP]_i^k - \kappa[RR]_i^k - \mu(a)[RR]_i^k \qquad \text{(Sys.5)}$$

The function $\mathrm{g}(\cdot)$ determines the rate at which systemic immunity develops from dual persistent infection. For the SISPminRS model, we used $\mathrm{g}(\cdot) = \min(\cdot)$, i.e. clearance of dual persistent infection is determined by the anatomic site with slowest clearance. For the SISPmaxRS model, we set $\mathrm{g}(\cdot) = \max(\cdot)$, i.e. clearance is determined by the anatomic site with fastest clearance. Infection hazards are modified as described before.

S2.4 SI(S)L models

Suppose that incident infections may be cleared at rates γ_{10} and γ_{01} for penile and anal infections, respectively, or develop into a state of local latency, at corresponding

rates ψ_{10} and ψ_{01}. In addition, dual infections may be cleared concomitantly at a rate that is determined by the anatomic site with slowest clearance, or turn into a state of dual latency at a rate that is determined by the anatomic site with fastest immune evasion, i.e. $\max(\psi_{10}, \psi_{01})$. Latent infections reactivate at a constant rate ϱ, and reactivated infections return to latency at the same rate at which latency developed from incident infections. Viral clearance, i.e. elimination of the virus from the penile or the anal site, no longer occurs once latency has developed. Note that these considerations were chosen to obtain maximal contrast with the SIS model. Using Q to denote the state of reactivated infection, the model becomes:

$$\partial_a[SS]_i^k = \Theta_i^k(a) - \left([\lambda_{10}]_i^k + [\lambda_{01}]_i^k + [\lambda_{11}]_i^k\right)[SS]_i^k + \gamma_{10}[IS]_i^k + \gamma_{01}[SI]_i^k + \min(\gamma_{10}, \gamma_{01})[II]_i^k - \mu(a)[SS]_i^k$$

$$\partial_a[IS]_i^k = [\lambda_{10}]_i^k[SS]_i^k - [\lambda_{*1}]_i^k[IS]_i^k - (\gamma_{10} + \psi_{10})[IS]_i^k - \mu(a)[IS]_i^k$$

$$\partial_a[SI]_i^k = [\lambda_{01}]_i^k[SS]_i^k - [\lambda_{1*}]_i^k[SI]_i^k - (\gamma_{01} + \psi_{01})[SI]_i^k - \mu(a)[SI]_i^k$$

$$\partial_a[II]_i^k = [\lambda_{11}]_i^k[SS]_i^k + [\lambda_{*1}]_i^k[IS]_i^k + [\lambda_{1*}]_i^k[SI]_i^k - (\min(\gamma_{10}, \gamma_{01}) + \max(\psi_{10}, \psi_{01}))[II]_i^k - \mu(a)[II]_i^k$$

$$\partial_a[LS]_i^k = \zeta_{10}\left([IS]_i^k + [QS]_i^k\right) - [\lambda_{*1}]_i^k[LS]_i^k - \varrho[LS]_i^k - \mu(a)[LS]_i^k$$

$$\partial_a[SL]_i^k = \zeta_{01}\left([SI]_i^k + [SQ]_i^k\right) - [\lambda_{1*}]_i^k[SL]_i^k - \varrho[SL]_i^k - \mu(a)[SL]_i^k$$

$$\partial_a[LI]_i^k = [\lambda_{*1}]_i^k[LS]_i^k + \psi_{10}[QI]_i^k - \psi_{01}[LI]_i^k - \varrho[LI]_i^k - \mu(a)[LI]_i^k$$

$$\partial_a[IL]_i^k = [\lambda_{1*}]_i^k[SL]_i^k + \psi_{01}[IQ]_i^k - \psi_{10}[IL]_i^k - \varrho[IL]_i^k - \mu(a)[LI]_i^k$$

$$\partial_a[LL]_i^k = \max(\psi_{10}, \psi_{01})[II]_i^k + \psi_{01}[LI]_i^k + \psi_{10}[IL]_i^k - 2\varrho[LL]_i^k - \mu(a)[LL]_i^k$$

$$\partial_a[QS]_i^k = \varrho[LS]_i^k - [\lambda_{*1}]_i^k[QS]_i^k - \psi_{10}[QS]_i^k - \mu(a)[QS]_i^k$$

$$\partial_a[SQ]_i^k = \varrho[SL]_i^k - [\lambda_{1*}]_i^k[SQ]_i^k - \psi_{01}[SQ]_i^k - \mu(a)[QS]_i^k$$

$$\partial_a[QI]_i^k = [\lambda_{*1}]_i^k[QS]_i^k + \varrho[LI]_i^k - \psi_{10}[QI]_i^k - \psi_{01}[QI]_i^k - \mu(a)[QI]_i^k$$

$$\partial_a[IQ]_i^k = [\lambda_{1*}]_i^k[SQ]_i^k + \varrho[IL]_i^k - \psi_{01}[IQ]_i^k - \psi_{10}[IQ]_i^k - \mu(a)[IQ]_i^k$$

$$\partial_a[QL]_i^k = \varrho[LL]_i^k + \zeta_{01}[QI]_i^k + \psi_{01}[QQ]_i^k - \varrho[QL]_i^k - \mu(a)[QL]_i^k$$

$$\partial_a[LQ]_i^k = \varrho[LL]_i^k + \zeta_{10}[IQ]_i^k + \psi_{10}[QQ]_i^k - \varrho[LQ]_i^k - \mu(a)[LQ]_i^k$$

$$\partial_a[QQ]_i^k = \varrho[QL]_i^k + \varrho[LQ]_i^k - (\psi_{10} + \psi_{01})[QQ]_i^k - \mu(a)[QQ]_i^k \qquad \text{(Sys.6)}$$

The fraction of incident infections that develop into latency is approximated by the ratios $\psi_{10}/(\gamma_{10} + \psi_{10})$ for penile infection, and $\psi_{01}/(\gamma_{01} + \psi_{01})$ for anal infection. Therefore, the SIL, SIS33L and SIS10L models are obtained by setting these fractions to 1·0, 0·33 and 0·10, respectively. Note that in the SIL model, this implies setting $\gamma_{10} = 0$ and $\gamma_{01} = 0$, i.e. all incident infections become latent. Infection hazards are modified by considering all infectious compartments in the terms $[\mathrm{P}_{..}(a', t)]_j^l$, i.e. all compartments involving I or Q at the relevant anatomic site, and summing $N(a, t)$ over all compartments.

S2.5 SI(R)L models

Now suppose that clearance of infection leads either to latency or to systemic immunity, at the rates ψ_{10} and γ_{10} for penile-only infections, and at the rates ψ_{01} and γ_{01} for anal-only infections, respectively. With otherwise similar assumptions as in the SI(S)L models, these modifications lead to the following SI(R)L model:

$$\partial_a[SS]_i^k = \Theta_i^k(a) - \left([\lambda_{10}]_i^k + [\lambda_{01}]_i^k + [\lambda_{11}]_i^k\right)[SS]_i^k - \mu(a)[SS]_i^k$$

$$\partial_a[IS]_i^k = [\lambda_{10}]_i^k[SS]_i^k - [\lambda_{*1}]_i^k[IS]_i^k - (\gamma_{10} + \psi_{10})[IS]_i^k - \mu(a)[IS]_i^k$$

$$\partial_a[SI]_i^k = [\lambda_{01}]_i^k[SS]_i^k - [\lambda_{1*}]_i^k[SI]_i^k - (\gamma_{01} + \psi_{01})[SI]_i^k - \mu(a)[SI]_i^k$$

$$\partial_a[II]_i^k = [\lambda_{11}]_i^k[SS]_i^k + [\lambda_{*1}]_i^k[IS]_i^k + [\lambda_{1*}]_i^k[SI]_i^k - (\min(\gamma_{10}, \gamma_{01}) + \max(\psi_{10}, \psi_{01}))[II]_i^k - \mu(a)[II]_i^k$$

$$\partial_a[RR]_i^k = \gamma_{10}[IS]_i^k + \gamma_{01}[SI]_i^k + \min(\gamma_{10}, \gamma_{01})\,[II]_i^k - \mu(a)[RR]_i^k \qquad \text{(Sys.7)}$$

Equations for the remainder of model compartments are identical to the SI(S)L models. Again, the fraction of incident infections that develop into latency is approximated by the ratios $\psi_{10}/(\gamma_{10}+\psi_{10})$ for penile infection, and $\psi_{01}/(\gamma_{01}+\psi_{01})$ for anal infection. Setting $\gamma_{10}=0$ and $\gamma_{01}=0$ yields the same SIL model as before. The SIR33L and SIR10L models are obtained by setting these fractions to 0·33 and 0·10, respectively.

S2.6 SIS model upon vaccination

We illustrate how the SIS model for penile-anal HPV16 transmission can be extended to accommodate targeted vaccination of (possibly infected) MSM or preadolescent boys' vaccination. Other HPV16 transmission models can be extended analogously. To model post-vaccination dynamics, we also let go of the steady state assumption to obtain a system of partial differential equations, with d_t denoting derivative by age plus calendar time, e.g. $d_t[SS]_i^k=\frac{d[SS(a,t)]_i^k}{dt}=\frac{\partial[SS(a,t)]_i^k}{\partial a}+\frac{\partial[SS(a,t)]_i^k}{\partial t}$.

Basically, vaccination is accommodated by tripling the number of model compartments, i.e. in the SIS model the number of compartments increases from $\{SS,SI,IS,II\}$ to $\{SS,SI,IS,II,SS|V,SI|V,IS|V,II|V,SS|NV,SI|NV,IS|NV,II|NV\}$ where $|V$ denotes "conditional on being successfully vaccinated" and $|NV$ denotes "conditional on being unsuccessfully vaccinated". The rate at which individuals get successfully vaccinated is a product of the rate of vaccine uptake $\sigma(a,t)$, itself dependent on age and calendar time, and of the probability π that a vaccinated individual derives vaccine-induced protection against infection. This probability, sometimes called "vaccine take", possibly depends on infection status at the time of immunization and

was thus considered state-specific, i.e. $\sigma_{SS}(a,t) = \pi_{SS}\sigma(a,t)$, $\sigma_{IS}(a,t) = \pi_{IS}\sigma(a,t)$, etc. Successfully vaccinated individuals experience a reduced hazard of infection compared to non-vaccinated (including unsuccessfully vaccinated) individuals, with ϖ denoting the hazard reduction. The SIS model for targeted vaccination is thus given by:

$$d_t[SS]_i^k = \Theta_i^k(a) - \left([\lambda_{10}]_i^k + [\lambda_{01}]_i^k + [\lambda_{11}]_i^k\right)[SS]_i^k + \gamma_{10}[IS]_i^k + \gamma_{01}[SI]_i^k - (\mu(a) + \sigma_{SS}(a,t))[SS]_i^k$$

$$d_t[IS]_i^k = [\lambda_{10}]_i^k[SS]_i^k - [\lambda_{*1}]_i^k[IS]_i^k - \gamma_{10}[IS]_i^k + \gamma_{01}[II]_i^k - (\mu(a) + \sigma_{IS}(a,t))[IS]_i^k$$

$$d_t[SI]_i^k = [\lambda_{01}]_i^k[SS]_i^k - [\lambda_{1*}]_i^k[SI]_i^k - \gamma_{01}[SI]_i^k + \gamma_{10}[II]_i^k - (\mu(a) + \sigma_{SI}(a,t))[SI]_i^k$$

$$d_t[II]_i^k = [\lambda_{11}]_i^k[SS]_i^k + [\lambda_{*1}]_i^k[IS]_i^k + [\lambda_{1*}]_i^k[SI]_i^k - (\gamma_{10} + \gamma_{01})[II]_i^k - (\mu(a) + \sigma_{II}(a,t))[II]_i^k$$

$$d_t[SS|V]_i^k = \sigma_{SS}(a,t)[SS]_i^k - (1-\varpi)\left([\lambda_{10}]_i^k + [\lambda_{01}]_i^k + [\lambda_{11}]_i^k\right)[SS|V]_i^k + \gamma_{10}[IS|V]_i^k + \gamma_{01}[SI|V]_i^k - \mu(a)[SS|V]_i^k$$

$$d_t[IS|V]_i^k = \sigma_{IS}(a,t)[IS]_i^k + (1-\varpi)\left([\lambda_{10}]_i^k[SS|V]_i^k - [\lambda_{*1}]_i^k[IS|V]_i^k\right) - \gamma_{10}[IS|V]_i^k + \gamma_{01}[II|V]_i^k - \mu(a)[IS|V]_i^k$$

$$d_t[SI|V]_i^k = \sigma_{SI}(a,t)[SI]_i^k + (1-\varpi)\left([\lambda_{01}]_i^k[SS|V]_i^k - [\lambda_{1*}]_i^k[SI|V]_i^k\right) - \gamma_{01}[SI|V]_i^k + \gamma_{10}[II|V]_i^k - \mu(a)[SI|V]_i^k$$

$$d_t[II|V]_i^k = \sigma_{II}(a,t)[II]_i^k + (1-\varpi)\left([\lambda_{11}]_i^k[SS|V]_i^k + [\lambda_{*1}]_i^k[IS|V]_i^k + [\lambda_{1*}]_i^k[SI|V]_i^k\right) - (\gamma_{10} + \gamma_{01})[II|V]_i^k - \mu(a)[II|V]_i^k$$

$$d_t[SS|NV]_i^k = (1-\pi_{SS})\sigma(a,t)[SS]_i^k - \left([\lambda_{10}]_i^k + [\lambda_{01}]_i^k + [\lambda_{11}]_i^k\right)[SS|NV]_i^k + \gamma_{10}[IS|NV]_i^k + \gamma_{01}[SI|NV]_i^k - \mu(a)[SS|NV]_i^k$$

$$d_t[IS|NV]_i^k = (1-\pi_{IS})\sigma(a,t)[IS]_i^k + \left([\lambda_{10}]_i^k[SS|NV]_i^k - [\lambda_{*1}]_i^k[IS|NV]_i^k\right) - \gamma_{10}[IS|NV]_i^k + \gamma_{01}[II|NV]_i^k - \mu(a)[IS|NV]_i^k$$

$$d_t[SI|NV]_i^k = (1-\pi_{SI})\sigma(a,t)[SI]_i^k + \left([\lambda_{01}]_i^k[SS|NV]_i^k - [\lambda_{1*}]_i^k[SI|NV]_i^k\right) - \gamma_{01}[SI|NV]_i^k + \gamma_{10}[II|NV]_i^k - \mu(a)[SI|NV]_i^k$$

$$d_t[II|NV]_i^k = (1-\pi_{II})\sigma(a,t)[II]_i^k + \left([\lambda_{11}]_i^k[SS|NV]_i^k + [\lambda_{*1}]_i^k[IS|NV]_i^k + [\lambda_{1*}]_i^k[SI|NV]_i^k\right) - (\gamma_{10} + \gamma_{01})[II|NV]_i^k - \mu(a)[II|NV]_i^k \qquad \text{(Sys.8)}$$

Infection hazards are modified by also considering vaccinated compartments in the terms $[\mathrm{P}_{..}(a',t)]_j^l$ in relation to the infection hazard under consideration. For instance, transmission to the penile site only becomes dependent on

$$[\rho_{10}]_{ij}\frac{\beta_{1*}\left([SI(a',t)]_j^l+[SI|V(a',t)]_j^l+[SI|NV(a',t)]_j^l+[II(a',t)]_j^l+[II|V(a',t)]_j^l+[II|NV(a',t)]_j^l\right)}{\mathbb{p}_l\mathbb{q}_jN(a',t)}+$$

$$[\rho_{11}]_{ij}\frac{\beta_{1*}\left([SI(a',t)]_j^l+[SI|V(a',t)]_j^l+[SI|NV(a',t)]_j^l+(1-\beta_{*1})\left([II(a',t)]_j^l+[II|V(a',t)]_j^l+[II|NV(a',t)]_j^l\right)\right)}{\mathbb{p}_l\mathbb{q}_jN(a',t)}.$$

with $N(a,t)$ summed over all compartments, and so on. For simplicity, we assume random mixing between vaccinated and non-vaccinated individuals, and similar transmissibility of vaccinated and non-vaccinated individuals, once infected.

The SIS model for preadolescent boys' vaccination is given by:

$$d_t[SS]_i^k=(1-v(a,t))\Theta_i^k(a)-\left([\lambda_{10}]_i^k+[\lambda_{01}]_i^k+[\lambda_{11}]_i^k\right)[SS]_i^k+\gamma_{10}[IS]_i^k+\gamma_{01}[SI]_i^k-\mu(a)[SS]_i^k$$

$$d_t[IS]_i^k=[\lambda_{10}]_i^k[SS]_i^k-[\lambda_{*1}]_i^k[IS]_i^k-\gamma_{10}[IS]_i^k+\gamma_{01}[II]_i^k-\mu(a)[IS]_i^k$$

$$d_t[SI]_i^k=[\lambda_{01}]_i^k[SS]_i^k-[\lambda_{1*}]_i^k[SI]_i^k-\gamma_{01}[SI]_i^k+\gamma_{10}[II]_i^k-\mu(a)[SI]_i^k$$

$$d_t[II]_i^k=[\lambda_{11}]_i^k[SS]_i^k+[\lambda_{*1}]_i^k[IS]_i^k+[\lambda_{1*}]_i^k[SI]_i^k-(\gamma_{10}+\gamma_{01})[II]_i^k-\mu(a)[II]_i^k$$

$$d_t[SS|V]_i^k=v(a,t)\Theta_i^k(a)-(1-\varpi)\left([\lambda_{10}]_i^k+[\lambda_{01}]_i^k+[\lambda_{11}]_i^k\right)[SS|V]_i^k+\gamma_{10}[IS|V]_i^k+\gamma_{01}[SI|V]_i^k-\mu(a)[SS|V]_i^k$$

$$d_t[IS|V]_i^k=(1-\varpi)\left([\lambda_{10}]_i^k[SS|V]_i^k-[\lambda_{*1}]_i^k[IS|V]_i^k\right)-\gamma_{10}[IS|V]_i^k+\gamma_{01}[II|V]_i^k-\mu(a)[IS|V]_i^k$$

$$d_t[SI|V]_i^k=(1-\varpi)\left([\lambda_{01}]_i^k[SS|V]_i^k-[\lambda_{1*}]_i^k[SI|V]_i^k\right)-\gamma_{01}[SI|V]_i^k+\gamma_{10}[II|V]_i^k-\mu(a)[SI|V]_i^k$$

$$d_t[II|V]_i^k=(1-\varpi)\left([\lambda_{11}]_i^k[SS|V]_i^k+[\lambda_{*1}]_i^k[IS|V]_i^k+[\lambda_{1*}]_i^k[SI|V]_i^k\right)-(\gamma_{10}+\gamma_{01})[II|V]_i^k-\mu(a)[II|V]_i^k$$

$$d_t[SS|NV]_i^k=(1-v(a,t))\Theta_i^k(a)-\left([\lambda_{10}]_i^k+[\lambda_{01}]_i^k+[\lambda_{11}]_i^k\right)[SS|NV]_i^k+\gamma_{10}[IS|NV]_i^k+\gamma_{01}[SI|NV]_i^k-\mu(a)[SS|NV]_i^k$$

$d_t[IS|NV]_i^k = \left([\lambda_{10}]_i^k[SS|NV]_i^k - [\lambda_{*1}]_i^k[IS|NV]_i^k\right) - \gamma_{10}[IS|NV]_i^k + \gamma_{01}[II|NV]_i^k - \mu(a)[IS|NV]_i^k$

$d_t[SI|NV]_i^k = \left([\lambda_{01}]_i^k[SS|NV]_i^k - [\lambda_{1*}]_i^k[SI|NV]_i^k\right) - \gamma_{01}[SI|NV]_i^k + \gamma_{10}[II|NV]_i^k - \mu(a)[SI|NV]_i^k$

$d_t[II|V]_i^k = \left([\lambda_{11}]_i^k[SS|NV]_i^k + [\lambda_{*1}]_i^k[IS|NV]_i^k + [\lambda_{1*}]_i^k[SI|NV]_i^k\right) - (\gamma_{10} + \gamma_{01})[II|NV]_i^k - \mu(a)[II|NV]_i^k$ (Sys.9)

Here, $v(a,t)$ denotes the probability of being successfully vaccinated in preadolescence, i.e. prior to sexual debut. This probability is informed by vaccine coverage in eligible cohorts, and depends on age and calendar time because vaccinated individuals enter the at-risk population at various times since immunization.
