# Supplementary material for: Potential effectiveness of prophylactic HPV immunization for men who have sex with men in the Netherlands: A multi-model approach
Source: PLoS Med. 2019 Mar 4;16(3):e1002756. doi: 10.1371/journal.pmed.1002756 (PMC6398832; doi:10.1371/journal.pmed.1002756)
Supplement: S3 Text — (PDF) [file pmed.1002756.s020.pdf]

S3 TEXT: FIT TO H2M STUDY DATA

PART III OF IV OF THE SUPPLEMENTARY ANNEX TO

POTENTIAL EFFECTIVENESS OF PROPHYLACTIC HPV IMMUNIZATION FOR MEN WHO HAVE SEX WITH MEN IN THE NETHERLANDS: A MULTI-MODEL APPROACH.

Johannes A Bogaards*, Sofie H Mooij, Maria Xiridou, Maarten F Schim van der Loeff

* Author to whom correspondence should be addressed: hans.bogaards@rivm.nl

TABLE OF CONTENTS

Supplement III: Fit to H2M study data *page 2*

S3.1 Fit to HPV16 clearance during follow-up *page 2*

S3.2 Fit to HPV16 prevalence at baseline *page 5*

Supplementary References *page 8*

Legends to Supplementary Figures *page 9*

Supplementary Tables C1-C21 *page 10*

Supplement III: Fit to H2M study data

We defined the likelihood of the vector of parameter values θ_S, associated with model S, given H2M study data x, as follows:

$$\mathcal{L}(\theta_S|x) \propto p(x_0|\theta_S)p(T_{1*}, T_{*1}|\Gamma_{1*}, \Gamma_{*1}) \quad \text{(Eq.7)}$$

Here, x_0 denotes site-specific HPV16 infection prevalence at baseline, whereas T_{1*} and T_{*1} denote event times of penile and anal HPV16 infection clearance, respectively, during follow-up. The parameters associated with site-specific progression and clearance are grouped according to $\Gamma_{1*} = \{\gamma_{10}, \zeta_{10}, \xi_{10}, \psi_{10}\}$ and $\Gamma_{*1} = \{\gamma_{01}, \zeta_{01}, \xi_{01}, \psi_{01}\}$.

Parameter estimates were obtained by an approximate maximum-likelihood (ML) procedure, consisting of separate optimization of each of the following product terms:

$$\hat{\mathcal{L}}(\theta_S|x) \propto p\left(x_0|\theta_S \backslash \{\hat{\Gamma}_{1*}, \hat{\Gamma}_{*1}\}\right)p\left(T_{1*}|\hat{\Gamma}_{1*}\right)p\left(T_{*1}|\hat{\Gamma}_{*1}\right) \quad \text{(Eq.8)}$$

The basic assumption here is that $\{\hat{\Gamma}_{1*}, \hat{\Gamma}_{*1}\}$ contribute to $p(x_0|\theta_S)$ chiefly through their correlation with ML estimates related to transmissibility, waning of natural immunity and reactivation of latent infections (if applicable). In addition, we assume that the joint ML parameters of site-specific progression and clearance can be approximated by separate likelihood optimization of penile and anal clearance events during follow-up.

S3.1 Fit to HPV16 clearance during follow-up

The parameters $\{\hat{\Gamma}_{1*}, \hat{\Gamma}_{*1}\}$ were estimated by maximizing the log-likelihood for interval-censored survival data:

$$\log p(T_{..}|\Gamma_{..}) = \sum_{i=1}^{n} \delta_i \log[S(l_i) - S(r_i)] + (1 - \delta_i) \log S(l_i) \quad \text{(Eq.9)}$$

with $\delta_i = 1$ if clearance was observed within the interval $(l_i, r_i]$ for the i-th individual, $\delta_i = 0$ if it was right-censored with last relevant observation time l_i, and $S(t)$ the survival function of site-specific HPV16 infection. This function defines the probability that an infection is not cleared before a specified time t during follow-up.

In base-case analyses, clearance was defined as at least one positive test result for HPV16 followed by two consecutive negative visits, and we used matching risk and censoring definitions, i.e. individuals were censored if the penultimate visit was positive. By this definition, 31 HIV-negative individuals were at risk for clearing a penile HPV16 infection (17 prevalent and 14 incident cases; total observation time: 22·1 person-years; 19 events observed), and 90 for clearing an anal HPV16 infection (58 prevalent and 32 incident cases; 76·2 person-years; 42 events).

In sensitivity analyses (S6 Fig), clearance was defined as at least one positive test result for HPV16 followed by one negative visit. By this definition, 46 HIV-negative individuals were at risk for clearing a penile HPV16 infection (17 prevalent and 29 incident cases; 26·3 person-years; 36 events), and 96 for clearing an anal HPV16 infection (58 prevalent and 38 incident cases; 77·5 person-years; 75 events).

For models without latency, we fitted the survival function:

$$S(t) = \frac{\gamma-\xi}{\gamma+\zeta-\xi}\exp\{-(\gamma+\zeta)t\} + \frac{\zeta}{\gamma+\zeta-\xi}\exp\{-\xi t\} \qquad \text{(Eq.10)}$$

This biphasic clearance model reduces to simple exponential form for $\zeta = 0$, in which case ξ is not defined. For comparison, we also considered the model $S(t) = \exp\{-(\gamma t)^{\eta}\}$ for Weibull-distributed clearance times to check for a decreasing hazard over time, corresponding to $\eta < 1$.

For models with latency, we fitted the survival function

$$S(t) = (1 - l)\exp\{-(\psi/\mathrm{frac})t\} + l\exp\{-\psi t\} \qquad \text{(Eq.11)}$$

Here, $\mathrm{frac} = \psi/(\gamma + \psi)$ denotes the fraction of incident infections turning into latency, and l denotes the proportion of observed individuals at risk for clearance with a reactivated latent infection. Thus, observed clearance is a mixture of exponentials at rates $\{\gamma, \zeta\}$, with $\gamma = \psi/\mathrm{frac} - \psi$. Latent infections were assumed to test negative for HPV16 in the SPF10-PCR DEIA/LiPA25 system [1]. Since $\gamma = 0$ in the SIL model, l is not defined and clearance reduces to simple exponential form. In the SIR33L and SIR10L models, reinfection is not considered and we assumed that observed clearance mostly informed on return to latency, i.e. we set $l = 1$. As a result, we adopted similar values for ψ as in the SIL model. In the SIS33L and SIS10L models, reinfection is possible and we estimated l together with ψ from event times of penile and anal HPV16 infection clearance.

Log-likelihood optimization for the various models was performed with use of the `optim` function in `R` version 3.4.3 [2], with check (if possible) of ML estimates by the lifereg procedure in SAS version 9.4 (SAS Institute Inc., Cary, NC, USA), on a 64-bit platform. To compare the model-based survival functions to a non-parametric empirical alternative, we also constructed survival functions with 95% confidence limits from a generalization of Kaplan-Meier estimates to interval-censored data [3]. Results of the various model-fitting procedures are given in the following table.

Table B1. Maximum-likelihood parameters associated with site-specific progression and clearance

Model	ML estimates in base-case analyses	log Likelihood	AIC
Exponential			
Penile	γ = 0·87 yr^{-1}	−34·69	71·39
Anal	γ = 0·54 yr^{-1}	−95·60	193·20
Weibull			
Penile	γ = 0·82 yr^{-1}; η = 0·68 (se 0·18)	−33·64	71·29
Anal	γ = 0·45 yr^{-1}; η = 0·71 (se 0·13)	−93·77	191·55
Biphasic			
Penile	γ = 3·40 yr^{-1}; ζ = 5·48 yr^{-1}; ξ = 0·43 yr^{-1}	−32·61	71·23
Anal	γ = 1·30 yr^{-1}; ζ = 3·47 yr^{-1}; ξ = 0·33 yr^{-1}	−92·96	191·93
Latent mixture			
Penile	frac = 0·10: l = 0·67; ψ = 0·51 yr^{-1}	−32·90	69·80
	frac = 0·33: l = 0·64; ψ = 0·61 yr^{-1}	−34·13	72·27
Anal	frac = 0·10: l = 0·74; ψ = 0·31 yr^{-1}	−93·05	190·10
	frac = 0·33: l = 0·65; ψ = 0·36 yr^{-1}	−94·71	193·41
Model	ML estimates in sensitivity analyses	log Likelihood	AIC
Exponential			
Penile	γ = 1·43 yr^{-1}	−48·07	98·14
Anal	γ = 0·99 yr^{-1}	−120·48	244·96
Weibull			
Penile	γ = 1·65 yr^{-1}; η = 0·67 (se 0·13)	−45·71	95·41
Anal	γ = 1·03 yr^{-1}; η = 0·83 (se 0·11)	−121·48	244·95
Biphasic			
Penile	γ = 4·23 yr^{-1}; ζ = 3·06 yr^{-1}; ξ = 0·57 yr^{-1}	−43·61	93·23
Anal	γ = 1·79 yr^{-1}; ζ = 3·11 yr^{-1}; ξ = 0·75 yr^{-1}	−119·74	245·48
Latent mixture			
Penile	frac = 0·10: l = 0·46; ψ = 0·61 yr^{-1}	−43·68	91·36
	frac = 0·33: l = 0·48; ψ = 0·93 yr^{-1}	−45·98	95·96
Anal	frac = 0·10: l = 0·66; ψ = 0·74 yr^{-1}	−120·31	244·63
	frac = 0·33: l = 0·78; ψ = 0·76 yr^{-1}	−119·84	243·68

S3.2 Fit to HPV16 prevalence at baseline

Parameters related to transmissibility, waning of natural immunity and reactivation of latent infections (if applicable) were estimated by maximizing the conditional log-likelihood for HPV16 infection prevalence among HIV-negatives at H2M study entry:

$$\log p\left(x_0 \middle| \theta_S \backslash \left\{\hat{\Gamma}_{1*}, \hat{\Gamma}_{*1}\right\}\right) = C + \sum_{\mathrm{a}=0}^{a_\dagger} \sum_{i=0}^{1} \sum_{j=0}^{1} n_{ij}(\mathrm{a}) \log f_{ij}^{(S)}(\mathrm{a}) \qquad \text{(Eq.12)}$$

Here, $n_{00}(\mathrm{a})$ denotes the number of individuals aged a (integer) years testing negative for HPV16 at both sites, $n_{10}(\mathrm{a})$ the number of individuals testing positive at the penile site and negative at the anal site, and so on. The corresponding fractions predicted by model S at a years is denoted by $f_{00}^{(S)}(\mathrm{a})$, $f_{10}^{(S)}(\mathrm{a})$, et cetera. The likelihood for such data derives from a multinomial distribution, which scales with an unknown constant C that is immaterial for likelihood optimization. The distribution of HPV16-positivity among HIV-negatives as compared to all 775 H2M study participants with a valid test results for both anatomic samples is tabulated below:

Table B2. Baseline HPV16 status by anatomic site among all and HIV-negative H2M study participants

HPV16 status (all)	Penile neg	Penile pos	HPV16 status (HIV-negatives)	Penile neg	Penile pos
Anal neg	n=620	n=25	Anal neg	n=388	n=10
Anal pos	n=111	n=19	Anal pos	n=51	n=9

As stated, the multinomial likelihood was maximized with respect to $\{\beta_{10}, \beta_{01}, \kappa, \varrho\}$ conditional on ML estimates $\{\hat{\Gamma}_{1*}, \hat{\Gamma}_{*1}\}$ associated with site-specific progression and clearance. Model-predicted prevalence was computed from the transmission models at pre-vaccine equilibrium. The fraction of individuals testing HPV16-negative at both sites at a years was calculated as $\sum_k \sum_i \int_{\mathrm{a}}^{\mathrm{a}+1} [SS(a,t)]_i^k \mathrm{d}a \,/ \sum_k \sum_i \int_{\mathrm{a}}^{\mathrm{a}+1} [N(a,t)]_i^k \mathrm{d}a$ in the SIS model; as $\sum_k \sum_i \int_{\mathrm{a}}^{\mathrm{a}+1} \left([SS(a,t)]_i^k + [RR(a,t)]_i^k\right) \mathrm{d}a \,/ \sum_k \sum_i \int_{\mathrm{a}}^{\mathrm{a}+1} [N(a,t)]_i^k \mathrm{d}a$ in SIS(R)S models; and analogously in other models. This involved the numerical solution of the HPV16 transmission models in continuous time, which was done by backward finite differencing along the characteristic curves until a stable distribution over the model compartments was reached [4,5]. Log-likelihood optimization was done with probabilities β_{10}, β_{01} transformed to logit scale, and rates κ, ϱ (if applicable) to logarithmic scale. Optimization was performed with use of the `optim` function in R

version 3.4.3, with the various transmission models coded in C++ and compiled as 'dynamically loadable libraries' for use in R on a 64-bit platform [2].

Because models with natural immunity converge to those without in the limit of large κ, we set upper bounds on the waning of natural immunity in order to avoid duplicates in the set of candidate models to be used for prediction. Heuristically, we employed upper bounds for κ of 1·0, 0·33, and 0·10 per year in models where we assumed that clearance led to immunity in all instances, in 1:3 instances, or in 1:10 instances, respectively. These bounds were chosen heuristically to distinguish models, yet ensure satisfactory fit in the case of constrained ML optimization. Likewise, we employed an upper bound for ϱ of 1·0 per year in models that allowed for reactivation of latent infections, to obtain a meaningful definition of latency with regard to the 3-6 sampling interval in the H2M study. The results of the various model-fitting procedures are tabulated in supplementary tables C1 to C21 at end of this annex.

Site-specific transmission probabilities varied widely across the models (S7 Fig), but penile-to-anal transmissibility almost invariably exceeded anal-to-penile transmissibility. Models that assumed a higher degree of natural immunity generally required increased transmissibility to reproduce the observed prevalence of penile and anal HPV16 infections, especially in the SIR33L model where return to susceptibility was precluded. In addition, penile-to-anal transmissibility was generally higher when presuming a stronger degree of assortative mixing with respect to sexual activity.

SUPPLEMENTARY REFERENCES

1. Molijn A, Kleter B, Quint W, van Doorn LJ. Molecular diagnosis of human papillomavirus (HPV) infections. J Clin Virol. 2005;32(Suppl 1):S43-S51.

2. R Core Team. R: A Language and Environment for Statistical Computing. Vienna, Austria: R Foundation for Statistical Computing; 2017.

3. Fay MP, Shaw PA. Exact and asymptotic weighted logrank tests for interval censored data: the interval R package. J Stat Softw. 2010;36(2). pii:i02.

4. Iannelli M, Kim MY. Splitting methods for the numerical approximation of some models of age-structured population dynamics and epidemiology. Appl Math Comp. 1997;87(1):69-93.

5. Abia LM, Angulo O, López-Marcos JC. Age-structured population models and their numerical solution. Ecol Modelling. 2005;188(1):112-36.

LEGENDS TO SUPPLEMENTARY FIGURES

S6 Figure. Sensitivity analyses on HPV16 infection clearance. Survival functions were fitted with account of interval censoring, with clearance of A) penile and B) anal infections defined as at least one HPV16 DNA-negative test result preceded by one positive visit. Non-parametric survival functions with 95% confidence limits are from a generalization of Kaplan-Meier estimates to interval censored data (Fay & Shaw 2010).

S7 Figure. Site-specific HPV16 transmissibility estimates. Model-based estimates of A) penile-to-anal and B) anal-to-penile transmissibility (note the difference in scales), grouped according to HPV16 natural history assumptions, ordered as in Table 2 with colours reflecting immunity and latency assumptions. In C) and D) they are grouped according to sexual activity distribution; assortative mixing with respect to sexual activity; and assortative mixing with respect to preference for insertive/receptive anal sex. The SIR10L model is not included, as it was not used in prediction due to poor fit.

Table C1. ML estimates of SIS model parameters informed by baseline infection prevalence

Sexual activity ±	ϵ †	ϕ ‡	β_{01} §	β_{10} §	log Likelihood	AIC ¶
80%—20%	0	0·15	0·1482	0·0123	−249·34	767·27
		0·67	0·1488	0·0124	−249·33	767·24
	0·33	0·15	0·1580	0·0119	−249·49	767·58
		0·67	0·1589	0·0119	−249·46	767·50
	0·67	0·15	0·1710	0·0114	−249·80	768·19
		0·67	0·1723	0·0114	−249·74	768·06
90%—10%	0	0·15	0·1158	0·0161	−249·83	768·25
		0·67	0·1159	0·0161	−249·92	768·42
	0·33	0·15	0·1304	0·0156	−249·26	767·11
		0·67	0·1305	0·0156	−249·28	767·15
	0·67	0·15	0·1910	0·0155	−249·65	767·89
		0·67	0·1909	0·0155	−249·62	767·83
60%—10%	0	0.15	0·1221	0·0151	−249·53	767·64
		0.67	0·1222	0·0151	−249·59	767·77
	0·33	0.15	0·1365	0·0146	−249·26	767·10
		0.67	0·1366	0·0146	−249·27	767·13
	0·67	0.15	0·1883	0·0144	−249·57	767·72
		0.67	0·1884	0·0144	−249·53	767·66

± Percentage MSM with above- and below-average partner acquisition

† Assortativity with regard to sexual activity; ϵ = 0 denotes random mixing

‡ Assortativity with regard to preference for insertive/receptive anal sex; ϕ = 0·15 denotes random mixing

§ β_{01} denotes penile-to-anal transmissibility; β_{10} denotes anal-to-penile transmissibility

¶ Composite fit to H2M baseline and follow-up data

Table C2. ML estimates of SISPS model parameters informed by baseline infection prevalence

Sexual activity ±	ϵ †	ϕ ‡	β_{01} §	β_{10} §	log Likelihood	AIC ¶
80%—20%	0	0·15	0·1153	0·0080	−250·75	768·65
		0·67	0·1151	0·0080	−250·62	768·40
	0·33	0·15	0·1220	0·0076	−251·36	769·89
		0·67	0·1218	0·0076	−251·23	769·61
	0·67	0·15	0·1298	0·0072	−252·21	771·58
		0·67	0·1294	0·0072	−252·06	771·27
90%—10%	0	0·15	0·0894	0·0112	−248·92	765·00
		0·67	0·0894	0·0112	−248·92	764·99
	0·33	0·15	0·1021	0·0107	−249·39	765·93
		0·67	0·1019	0·0107	−249·35	765·86
	0·67	0·15	0·1547	0·0107	−250·93	769·01
		0·67	0·1545	0·0107	−250·89	768·93
60%—10%	0	0.15	0·0949	0·0103	−249·13	765·42
		0.67	0·0948	0·0103	−249·10	765·35
	0·33	0.15	0·1073	0·0099	−249·67	766·50
		0.67	0·1073	0·0099	−249·62	766·40
	0·67	0.15	0·1536	0·0097	−250·94	769·04
		0.67	0·1536	0·0097	−250·88	768·92

± Percentage MSM with above- and below-average partner acquisition

† Assortativity with regard to sexual activity; ϵ = 0 denotes random mixing

‡ Assortativity with regard to preference for insertive/receptive anal sex; ϕ = 0·15 denotes random mixing

§ β_{01} denotes penile-to-anal transmissibility; β_{10} denotes anal-to-penile transmissibility

¶ Composite fit to H2M baseline and follow-up data

Table C3. ML estimates of SIRS model parameters informed by baseline infection prevalence

Sexual activity ±	ϵ †	ϕ ‡	β_{01} §	β_{10} §	κ (yr^{-1})	log Likelihood	AIC ¶
80%—20%	0	0·15	0·4766	0·0368	1·0*	–250·09	770·76
		0·67	0·4756	0·0383	1·0*	–249·89	770·37
	0·33	0·15	0·6503	0·0401	1·0*	–250·70	771·98
		0·67	0·6455	0·0396	1·0*	–250·46	771·52
	0·67	0·15	0·9999	0·0411	1·0*	–251·84	774·27
		0·67	0·9999	0·0408	1·0*	–251·58	773·75
90%—10%	0	0·15	0·2251	0·0368	1·0*	–250·12	770·82
		0·67	0·2257	0·0364	1·0*	–250·41	771·41
	0·33	0·15	0·2715	0·0406	1·0*	–249·60	769·78
		0·67	0·2720	0·0399	1·0*	–249·76	770·11
	0·67	0·15	0·4053	0·0497	1·0*	–249·30	769·19
		0·67	0·4053	0·0488	1·0*	–249·34	769·27
60%—10%	0	0.15	0·2981	0·0413	0·852	–249·41	769·41
		0.67	0·4520	0·0617	0·530	–249·50	769·59
	0·33	0.15	0·3236	0·0393	1·0*	–249·34	769·27
		0.67	0·3262	0·0388	1·0*	–249·39	769·36
	0·67	0.15	0·4504	0·0430	1·0*	–249·45	769·48
		0.67	0·4529	0·0424	1·0*	–249·40	769·38

± Percentage MSM with above- and below-average partner acquisition

† Assortativity with regard to sexual activity; ϵ = 0 denotes random mixing

‡ Assortativity with regard to preference for insertive/receptive anal sex; ϕ = 0·15 denotes random mixing

§ β_{01} denotes penile-to-anal transmissibility; β_{10} denotes anal-to-penile transmissibility

¶ Composite fit to H2M baseline and follow-up data

* Upper bound of estimation interval

Table C4. ML estimates of SIS33RS model parameters informed by baseline infection prevalence

Sexual activity ±	ϵ †	ϕ ‡	β_{01} §	β_{10} §	κ (yr^{-1})	log Likelihood	AIC ¶
80%—20%	0	0·15	0·4255	0·0304	0·33*	–250·14	770·87
		0·67	0·4238	0·0303	0·33*	–249·98	770·55
	0·33	0·15	0·5826	0·0312	0·33*	–250·76	772·10
		0·67	0·5785	0·0310	0·33*	–250·55	771·69
	0·67	0·15	0·9999	0·0323	0·33*	–251·88	774·36
		0·67	0·9999	0·0320	0·33*	–251·63	773·84
90%—10%	0	0·15	0·2002	0·0316	0·33*	–250·23	771·05
		0·67	0·2005	0·0313	0·33*	–250·45	771·48
	0·33	0·15	0·2433	0·0348	0·33*	–249·68	769·94
		0·67	0·2434	0·0344	0·33*	–249·79	770·17
	0·67	0·15	0·3723	0·0428	0·33*	–249·39	769·37
		0·67	0·3719	0·0423	0·33*	–249·41	769·40
60%—10%	0	0.15	0·2361	0·0309	0·33*	–249.59	769·78
		0.67	0·2374	0·0307	0·33*	–249.71	770·00
	0·33	0.15	0·2848	0·0327	0·33*	–249.48	769·55
		0.67	0·2860	0·0325	0·33*	–249.53	769·64
	0·67	0.15	0·4025	0·0358	0·33*	–249.55	769·70
		0.67	0.4031	0·0355	0·33*	–249.52	769·63

± Percentage MSM with above- and below-average partner acquisition

† Assortativity with regard to sexual activity; ϵ = 0 denotes random mixing

‡ Assortativity with regard to preference for insertive/receptive anal sex; ϕ = 0·15 denotes random mixing

§ β_{01} denotes penile-to-anal transmissibility; β_{10} denotes anal-to-penile transmissibility

¶ Composite fit to H2M baseline and follow-up data

* Upper bound of estimation interval

Table C5. ML estimates of SIS10RS model parameters informed by baseline infection prevalence

Sexual activity ±	ϵ †	ϕ ‡	β_{01} §	β_{10} §	κ (yr^{-1})	log Likelihood	AIC ¶
80%—20%	0	0·15	0·3465	0·0231	0·10*	–250·61	771·81
		0·67	0·3455	0·0231	0·10*	–250·48	771·54
	0·33	0·15	0·4600	0·0230	0·10*	–251·25	773·09
		0·67	0·4567	0·0230	0·10*	–251·07	772·72
	0·67	0·15	0·8417	0·0231	0·10*	–252·47	775·53
		0·67	0·8286	0·0230	0·10*	–252·23	775·05
90%—10%	0	0·15	0·1759	0·0268	0·10*	–250·52	771·63
		0·67	0·1759	0·0267	0·10*	–250·70	771·99
	0·33	0·15	0·2131	0·0292	0·10*	–249·97	770·53
		0·67	0·2129	0·0290	0·10*	–250·06	770·71
	0·67	0·15	0·3301	0·0360	0·10*	–249·78	770·15
		0·67	0·3297	0·0356	0·10*	–249·78	770·14
60%—10%	0	0.15	0·2006	0·0256	0·10*	–250·04	770·67
		0.67	0·2013	0·0255	0·10*	–250·15	770·88
	0·33	0.15	0·2408	0·0268	0·10*	–249·92	770·42
		0.67	0·2413	0·0267	0·10*	–249·96	770·50
	0·67	0.15	0·3424	0·0294	0·10*	–250·02	770·62
		0.67	0·3430	0·0292	0·10*	–249·99	770·56

± Percentage MSM with above- and below-average partner acquisition

† Assortativity with regard to sexual activity; ϵ = 0 denotes random mixing

‡ Assortativity with regard to preference for insertive/receptive anal sex; ϕ = 0·15 denotes random mixing

§ β_{01} denotes penile-to-anal transmissibility; β_{10} denotes anal-to-penile transmissibility

¶ Composite fit to H2M baseline and follow-up data

* Upper bound of estimation interval

Table C6. ML estimates of SISPminRS model parameters informed by baseline infection prevalence

Sexual activity ±	ϵ †	ϕ ‡	β_{01} §	β_{10} §	κ (yr^{-1})	log Likelihood	AIC ¶
80%—20%	0	0·15	0·1797	0·0103	1·0*	–251·49	772·14
		0·67	0·1791	0·0103	1·0*	–251·36	771·87
	0·33	0·15	0·2126	0·0099	1·0*	–252·39	773·94
		0·67	0·2109	0·0098	1·0*	–252·25	773·65
	0·67	0·15	0·2856	0·0091	1·0*	–253·77	776·70
		0·67	0·2754	0·0091	1·0*	–253·63	776·42
90%—10%	0	0·15	0·1104	0·0146	1·0*	–248·96	767·08
		0·67	0·1105	0·0146	1·0*	–248·96	767·08
	0·33	0·15	0·1582	0·0191	0·516	–249·24	767·63
		0·67	0·1561	0·0187	0·539	–249·21	767·58
	0·67	0·15	0·3397	0·0345	0·253	–249·68	768·51
		0·67	0·3389	0·0342	0·255	–249·65	768·45
60%—10%	0	0.15	0·1222	0·0133	1·0*	–249·27	767·69
		0.67	0·1224	0·0133	1·0*	–249·22	767·60
	0·33	0.15	0·1438	0·0133	1·0*	–249·73	768·62
		0.67	0·1441	0·0133	1·0*	–249·67	768·49
	0·67	0.15	0·2532	0·0167	0·598	–250·58	770·32
		0.67	0·2573	0·0169	0·580	–250·50	770·15

± Percentage MSM with above- and below-average partner acquisition

† Assortativity with regard to sexual activity; ϵ = 0 denotes random mixing

‡ Assortativity with regard to preference for insertive/receptive anal sex; ϕ = 0·15 denotes random mixing

§ β_{01} denotes penile-to-anal transmissibility; β_{10} denotes anal-to-penile transmissibility

¶ Composite fit to H2M baseline and follow-up data

* Upper bound of estimation interval

Table C7. ML estimates of SISPmaxRS model parameters informed by baseline infection prevalence

Sexual activity ±	ϵ †	ϕ ‡	β_{01} §	β_{10} §	κ (yr^{-1})	log Likelihood	AIC ¶
80%—20%	0	0·15	0·1914	0·0124	1·0*	–250·79	770·73
		0·67	0·1915	0·0123	1·0*	–250·61	770·37
	0·33	0·15	0·2285	0·0119	1·0*	–251·58	772·32
		0·67	0·2271	0·0119	1·0*	–251·39	771·93
	0·67	0·15	0·3182	0·0112	1·0*	–252·87	774·89
		0·67	0·3074	0·0111	1·0*	–252·68	774·49
90%—10%	0	0·15	0·1175	0·0164	1·0*	–249·09	767·33
		0·67	0·1177	0·0164	1·0*	–249·13	767·41
	0·33	0·15	0·1384	0·0169	1·0*	–249·11	767·37
		0·67	0·1386	0·0169	1·0*	–249·09	767·33
	0·67	0·15	0·3211	0·0345	0·323	–249·41	767·98
		0·67	0·3164	0·0335	0·334	–249·38	767·92
60%—10%	0	0.15	0·1298	0·0152	1·0*	–249·12	767·39
		0.67	0·1303	0·0152	1·0*	–249·09	767·34
	0·33	0.15	0·1520	0·0154	1·0*	–249·39	767·94
		0.67	0·1523	0·0154	1·0*	–249·32	767·81
	0·67	0.15	0·2537	0·0190	0·660	–250·02	769·20
		0.67	0·2584	0·0192	0·638	–249·92	768·99

± Percentage MSM with above- and below-average partner acquisition

† Assortativity with regard to sexual activity; ϵ = 0 denotes random mixing

‡ Assortativity with regard to preference for insertive/receptive anal sex; ϕ = 0·15 denotes random mixing

§ β_{01} denotes penile-to-anal transmissibility; β_{10} denotes anal-to-penile transmissibility

¶ Composite fit to H2M baseline and follow-up data

* Upper bound of estimation interval

Table C8. ML estimates of SIR[local]S model parameters informed by baseline infection prevalence

Sexual activity ±	ϵ †	ϕ ‡	β_{01} §	β_{10} §	κ (yr^{-1})	log Likelihood	AIC ¶
80%—20%	0	0·15	0·3901	0·0216	1·0*	–249·55	769·69
		0·67	0·3893	0·0217	1·0*	–249·59	769·77
	0·33	0·15	0·5092	0·0215	1·0*	–249·53	769·64
		0·67	0·5073	0·0216	1·0*	–249·54	769·67
	0·67	0·15	0·9999	0·0235	0·884**	–249·58	769·75
		0·67	0·9490	0·0227	0·923**	–249·58	769·74
90%—10%	0	0·15	0·2013	0·0268	1·0*	–251·11	772·82
		0·67	0·2011	0·0267	1·0*	–251·37	773·32
	0·33	0·15	0·2453	0·0284	1·0*	–250·39	771·37
		0·67	0·2452	0·0283	1·0*	–250·57	771·72
	0·67	0·15	0·3799	0·0326	1·0*	–249·75	770·08
		0·67	0·3790	0·0324	1·0*	–249·84	770·27
60%—10%	0	0.15	0·2307	0·0249	1·0*	–250·37	771·33
		0.67	0·2318	0·0248	1·0*	–250·57	771·73
	0·33	0.15	0·2771	0·0256	1·0*	–250·03	770·66
		0.67	0·2780	0·0255	1·0*	–250·19	770·96
	0·67	0.15	0·3907	0·0270	1·0*	–249·73	770·04
		0.67	0·3911	0·0269	1·0*	–249·83	770·24

± Percentage MSM with above- and below-average partner acquisition

† Assortativity with regard to sexual activity; ϵ = 0 denotes random mixing

‡ Assortativity with regard to preference for insertive/receptive anal sex; ϕ = 0·15 denotes random mixing

§ β_{01} denotes penile-to-anal transmissibility; β_{10} denotes anal-to-penile transmissibility

¶ Composite fit to H2M baseline and follow-up data

* Upper bound of estimation interval

Table C9. ML estimates of SIS33R[local]S model parameters informed by baseline infection prevalence

Sexual activity ±	ϵ †	ϕ ‡	β_{01} §	β_{10} §	κ (yr^{-1})	log Likelihood	AIC ¶
80%—20%	0	0·15	0·3750	0·0208	0·33*	–249·70	769·99
		0·67	0·3746	0·0209	0·33*	–249·74	770·06
	0·33	0·15	0·4870	0·0206	0·33*	–249·70	769·98
		0·67	0·4858	0·0207	0·33*	–249·71	770·00
	0·67	0·15	0·8255	0·0203	0·33*	–249·80	770·18
		0·67	0·8194	0·0204	0·33*	–249·78	770·14
90%—10%	0	0·15	0·1954	0·0260	0·33*	–251·18	772·95
		0·67	0·1952	0·0259	0·33*	–251·42	773·43
	0·33	0·15	0·2374	0·0275	0·33*	–250·47	771·53
		0·67	0·2372	0·0274	0·33*	–250·63	771·85
	0·67	0·15	0·3676	0·0316	0·33*	–249·85	770·28
		0·67	0·3665	0·0314	0·33*	–249·93	770·45
60%—10%	0	0.15	0·2227	0·0241	0·33*	–250·47	771·53
		0.67	0·2238	0·0241	0·33*	–250·66	771·91
	0·33	0.15	0·2671	0·0248	0·33*	–250·14	770·87
		0.67	0·2680	0·0247	0·33*	–250·28	771·15
	0·67	0.15	0·3770	0·0262	0·33*	–249·85	770·28
		0.67	0·3779	0·0261	0·33*	–249·94	770·46

± Percentage MSM with above- and below-average partner acquisition

† Assortativity with regard to sexual activity; ϵ = 0 denotes random mixing

‡ Assortativity with regard to preference for insertive/receptive anal sex; ϕ = 0·15 denotes random mixing

§ β_{01} denotes penile-to-anal transmissibility; β_{10} denotes anal-to-penile transmissibility

¶ Composite fit to H2M baseline and follow-up data

* Upper bound of estimation interval

Table C10. ML estimates of SIS10R[local]S model parameters informed by baseline infection prevalence

Sexual activity ±	ϵ †	ϕ ‡	β_{01} §	β_{10} §	κ (yr^{-1})	log Likelihood	AIC ¶
80%—20%	0	0·15	0·3128	0·0181	0·10*	–250·05	770·68
		0·67	0·3134	0·0182	0·10*	–250·06	770·72
	0·33	0·15	0·3947	0·0177	0·10*	–250·12	770·83
		0·67	0·3944	0·0178	0·10*	–250·11	770·81
	0·67	0·15	0·6285	0·0171	0·10*	–250·36	771·30
		0·67	0·6268	0·0172	0·10*	–250·32	771·22
90%—10%	0	0·15	0·1752	0·0234	0·10*	–251·18	772·95
		0·67	0·1751	0·0234	0·10*	–251·38	773·36
	0·33	0·15	0·2111	0·0244	0·10*	–250·50	771·59
		0·67	0·2108	0·0243	0·10*	–250·63	771·84
	0·67	0·15	0·3274	0·0277	0·10*	–249·99	770·58
		0·67	0·3265	0·0275	0·10*	–250·04	770·68
60%—10%	0	0.15	0·1954	0·0217	0·10*	–250·59	771·78
		0.67	0·1962	0·0217	0·10*	–250·75	772·10
	0·33	0.15	0·2324	0·0221	0·10*	–250·29	771·16
		0.67	0·2330	0·0221	0·10*	–250·40	771·38
	0·67	0.15	0·3301	0·0233	0·10*	–250·06	770·70
		0.67	0·3302	0·0233	0·10*	–250·12	770·82

± Percentage MSM with above- and below-average partner acquisition

† Assortativity with regard to sexual activity; ϵ = 0 denotes random mixing

‡ Assortativity with regard to preference for insertive/receptive anal sex; ϕ = 0·15 denotes random mixing

§ β_{01} denotes penile-to-anal transmissibility; β_{10} denotes anal-to-penile transmissibility

¶ Composite fit to H2M baseline and follow-up data

* Upper bound of estimation interval

Table C11. ML estimates of SIR[penile]S model parameters informed by baseline infection prevalence

Sexual activity ±	ϵ †	ϕ ‡	β_{01} §	β_{10} §	κ (yr^{-1})	log Likelihood	AIC ¶
80%—20%	0	0·15	0·1563	0·0415	0·377	–249·20	768·99
		0·67	0·1553	0·0343	0·441	–249·21	769·00
	0·33	0·15	0·1718	0·0847	0·273	–249·21	769·01
		0·67	0·1700	0·0688	0·303	–249·21	769·01
	0·67	0·15	0·1954	0·2280	0·220	–249·23	769·05
		0·67	0·1942	0·2098	0·228	–249·24	769·06
90%—10%	0	0·15	0·1297	0·0220	1·0*	–250·15	770·88
		0·67	0·1300	0·0219	1·0*	–250·31	771·21
	0·33	0·15	0·1453	0·0234	1·0*	–249·44	769·46
		0·67	0·1455	0·0233	1·0*	–249·51	769·61
	0·67	0·15	0·2087	0·0359	0·695	–249·24	769·07
		0·67	0·2063	0·0334	0·771	–249·24	769·06
60%—10%	0	0.15	0·1332	0·0212	1·0*	–249·67	769·93
		0.67	0·1335	0·0211	1·0*	–249·80	770·19
	0·33	0.15	0·1477	0·0220	1·0*	–249·33	769·24
		0.67	0·1481	0·0219	1·0*	–249·39	769·37
	0·67	0.15	0·1935	0·0306	0·679	–249·24	769·07
		0.67	0·1923	0·0279	0·787	–249·24	769·08

± Percentage MSM with above- and below-average partner acquisition

† Assortativity with regard to sexual activity; ϵ = 0 denotes random mixing

‡ Assortativity with regard to preference for insertive/receptive anal sex; ϕ = 0·15 denotes random mixing

§ β_{01} denotes penile-to-anal transmissibility; β_{10} denotes anal-to-penile transmissibility

¶ Composite fit to H2M baseline and follow-up data

* Upper bound of estimation interval

Table C12. ML estimates of SIS33R[penile]S model parameters informed by baseline infection prevalence

Sexual activity ±	ϵ †	ϕ ‡	β_{01} §	β_{10} §	κ (yr^{-1})	log Likelihood	AIC ¶
80%—20%	0	0·15	0·1499	0·0182	0·33*	–249·31	769·20
		0·67	0·1507	0·0183	0·33*	–249·29	769·16
	0·33	0·15	0·1589	0·0180	0·33*	–249·45	769·48
		0·67	0·1599	0·0183	0·321	–249·39	769·37
	0·67	0·15	0·1708	0·0175	0·33*	–249·76	770·10
		0·67	0·1722	0·0187	0·290	–249·65	769·90
90%—10%	0	0·15	0·1289	0·0217	0·33*	–250·17	770·93
		0·67	0·1291	0·0216	0·33*	–250·33	771·24
	0·33	0·15	0·1442	0·0230	0·33*	–249·47	769·52
		0·67	0·1445	0·0229	0·33*	–249·54	769·66
	0·67	0·15	0·2030	0·0316	0·268	–249·32	769·22
		0·67	0·2015	0·0299	0·293	–249·30	769·18
60%—10%	0	0.15	0·1325	0·0208	0·33*	–249·70	769·99
		0.67	0·1329	0·0208	0·33*	–249·82	770·24
	0·33	0.15	0·1469	0·0216	0·33*	–249·36	769·31
		0.67	0·1474	0·0215	0·33*	–249·42	769·43
	0·67	0.15	0·1907	0·0260	0·284	–249·32	769·22
		0.67	0·1902	0·0245	0·320	–249·30	769·18

± Percentage MSM with above- and below-average partner acquisition

† Assortativity with regard to sexual activity; ϵ = 0 denotes random mixing

‡ Assortativity with regard to preference for insertive/receptive anal sex; ϕ = 0·15 denotes random mixing

§ β_{01} denotes penile-to-anal transmissibility; β_{10} denotes anal-to-penile transmissibility

¶ Composite fit to H2M baseline and follow-up data

* Upper bound of estimation interval

Table C13. ML estimates of SIS10R[penile]S model parameters informed by baseline infection prevalence

Sexual activity ±	ϵ †	ϕ ‡	β_{01} §	β_{10} §	κ (yr^{-1})	log Likelihood	AIC ¶
80%—20%	0	0·15	0·1514	0·0165	0·10*	–249·44	769·46
		0·67	0·1520	0·0165	0·10*	–249·42	769·42
	0·33	0·15	0·1610	0·0161	0·10*	–249·58	769·75
		0·67	0·1619	0·0162	0·10*	–249·53	769·65
	0·67	0·15	0·1736	0·0156	0·10*	–249·90	770·38
		0·67	0·1752	0·0157	0·10*	–249·80	770·19
90%—10%	0	0·15	0·1267	0·0203	0·10*	–250·20	770·99
		0·67	0·1269	0·0202	0·10*	–250·35	771·28
	0·33	0·15	0·1418	0·0211	0·10*	–249·54	769·66
		0·67	0·1421	0·0211	0·10*	–249·60	769·78
	0·67	0·15	0·1985	0·0252	0·10*	–249·51	769·61
		0·67	0·1984	0·0251	0·10*	–249·48	769·54
60%—10%	0	0.15	0·1312	0·0193	0·10*	–249·77	770·12
		0.67	0·1314	0·0193	0·10*	–249·88	770·35
	0·33	0.15	0·1457	0·0197	0·10*	–249·45	769·49
		0.67	0·1460	0·0197	0·10*	–249·50	769·59
	0·67	0.15	0·1907	0·0218	0·10*	–249·48	769·56
		0.67	0·1908	0·0217	0·10*	–249·46	769·50

± Percentage MSM with above- and below-average partner acquisition

† Assortativity with regard to sexual activity; ϵ = 0 denotes random mixing

‡ Assortativity with regard to preference for insertive/receptive anal sex; ϕ = 0·15 denotes random mixing

§ β_{01} denotes penile-to-anal transmissibility; β_{10} denotes anal-to-penile transmissibility

¶ Composite fit to H2M baseline and follow-up data

* Upper bound of estimation interval

Table C14. ML estimates of SIR[anal]S model parameters informed by baseline infection prevalence

Sexual activity ±	ϵ †	ϕ ‡	β_{01} §	β_{10} §	κ (yr^{-1})	log Likelihood	AIC ¶
80%—20%	0	0·15	0·3858	0·0144	1·0*	–249·60	769·78
		0·67	0·3877	0·0144	1·0*	–249·62	769·82
	0·33	0·15	0·5086	0·0139	1·0*	–249·59	769·77
		0·67	0·5104	0·0140	1·0*	–249·59	769·78
	0·67	0·15	0·9944	0·0137	0·899*	–249·66	769·92
		0·67	0·9641	0·0136	0·926*	–249·66	769·91
90%—10%	0	0·15	0·1801	0·0198	1·0*	–250·79	772·17
		0·67	0·1802	0·0198	1·0*	–250·96	772·50
	0·33	0·15	0·2210	0·0195	1·0*	–250·07	770·72
		0·67	0·2213	0·0196	1·0*	–250·16	770·90
	0·67	0·15	0·3586	0·0120	1·0*	–249·52	769·62
		0·67	0·3596	0·0120	1·0*	–249·55	769·68
60%—10%	0	0.15	0·2119	0·0179	1·0*	–250·23	771·06
		0.67	0·2135	0·0179	1·0*	–250·36	771·31
	0·33	0.15	0·2581	0·0176	1·0*	–249·88	770·35
		0.67	0·2598	0·0177	1·0*	–249·96	770·51
	0·67	0.15	0·3874	0·0176	1·0*	–249·60	769·78
		0.67	0·3898	0·0176	1·0*	–249·63	769·86

± Percentage MSM with above- and below-average partner acquisition

† Assortativity with regard to sexual activity; ϵ = 0 denotes random mixing

‡ Assortativity with regard to preference for insertive/receptive anal sex; ϕ = 0·15 denotes random mixing

§ β_{01} denotes penile-to-anal transmissibility; β_{10} denotes anal-to-penile transmissibility

¶ Composite fit to H2M baseline and follow-up data

* Upper bound of estimation interval

Table C15. ML estimates of SIS33R[anal]S model parameters informed by baseline infection prevalence

Sexual activity ±	ϵ †	ϕ ‡	β_{01} §	β_{10} §	κ (yr^{-1})	log Likelihood	AIC ¶
80%—20%	0	0·15	0·3728	0·0142	0·33*	–249·72	770·02
		0·67	0·3746	0·0142	0·33*	–249·74	770·06
	0·33	0·15	0·4887	0·0137	0·33*	–249·73	770·04
		0·67	0·4904	0·0138	0·33*	–249·73	770·05
	0·67	0·15	0·8364	0·0131	0·33*	–249·84	770·26
		0·67	0·8369	0·0131	0·33*	–249·83	770·24
90%—10%	0	0·15	0·1764	0·0195	0·33*	–250·84	772·27
		0·67	0·1764	0·0196	0·33*	–251·00	772·59
	0·33	0·15	0·2161	0·0193	0·33*	–250·13	770·84
		0·67	0·2164	0·0194	0·33*	–250·22	771·02
	0·67	0·15	0·3509	0·0197	0·33*	–249·60	769·79
		0·67	0·3517	0·0197	0·33*	–249·63	769·85
60%—10%	0	0.15	0·2065	0·0177	0·33*	–250·31	771·21
		0.67	0·2077	0·0177	0·33*	–250·44	771·46
	0·33	0.15	0·2507	0·0175	0·33*	–249·97	770·52
		0.67	0·2523	0·0175	0·33*	–250·05	770·68
	0·67	0.15	0·3758	0·0174	0·33*	–249·70	769·98
		0.67	0·3780	0·0174	0·33*	–249·73	770·05

± Percentage MSM with above- and below-average partner acquisition

† Assortativity with regard to sexual activity; ϵ = 0 denotes random mixing

‡ Assortativity with regard to preference for insertive/receptive anal sex; ϕ = 0·15 denotes random mixing

§ β_{01} denotes penile-to-anal transmissibility; β_{10} denotes anal-to-penile transmissibility

¶ Composite fit to H2M baseline and follow-up data

* Upper bound of estimation interval

Table C16. ML estimates of SIS10R[anal]S model parameters informed by baseline infection prevalence

Sexual activity ±	ϵ †	ϕ ‡	β_{01} §	β_{10} §	κ (yr^{-1})	log Likelihood	AIC ¶
80%—20%	0	0·15	0·3118	0·0137	0·10*	–249·96	770·50
		0·67	0·3134	0·0137	0·10*	–249·97	770·52
	0·33	0·15	0·3962	0·0132	0·10*	–250·04	770·67
		0·67	0·3979	0·0132	0·10*	–250·03	770·65
	0·67	0·15	0·6354	0·0125	0·10*	–250·28	771·15
		0·67	0·6367	0·0125	0·10*	–250·26	771·10
90%—10%	0	0·15	0·1620	0·0188	0·10*	–250·82	772·24
		0·67	0·1619	0·0189	0·10*	–250·97	772·53
	0·33	0·15	0·1968	0·0185	0·10*	–250·14	770·87
		0·67	0·1968	0·0186	0·10*	–250·22	771·02
	0·67	0·15	0·3175	0·0189	0·10*	–249·72	770·04
		0·67	0·3179	0·0189	0·10*	–249·74	770·07
60%—10%	0	0.15	0·1843	0·0172	0·10*	–250·37	771·33
		0.67	0·1851	0·0172	0·10*	–250·49	771·57
	0·33	0.15	0·2215	0·0169	0·10*	–250·05	770·69
		0.67	0·2224	0·0169	0·10*	–250·12	770·83
	0·67	0.15	0·3309	0·0169	0·10*	–250·85	770·29
		0.67	0·3319	0·0169	0·10*	–250·87	770·34

± Percentage MSM with above- and below-average partner acquisition

† Assortativity with regard to sexual activity; ϵ = 0 denotes random mixing

‡ Assortativity with regard to preference for insertive/receptive anal sex; ϕ = 0·15 denotes random mixing

§ β_{01} denotes penile-to-anal transmissibility; β_{10} denotes anal-to-penile transmissibility

¶ Composite fit to H2M baseline and follow-up data

* Upper bound of estimation interval

Table C17. ML estimates of SIL model parameters informed by baseline infection prevalence

Sexual activity ±	ϵ †	ϕ ‡	β_{01} §	β_{10} §	ϱ (yr^{-1})	log Likelihood	AIC ¶
80%—20%	0	0·15	0·0325	0·0018	1·0*	–248·57	767·72
		0·67	0·0326	0·0018	1·0*	–248·56	767·71
	0·33	0·15	0·0402	0·0017	1·0*	–248·68	767·95
		0·67	0·0402	0·0017	1·0*	–248·67	767·93
	0·67	0·15	0·0700	0·0016	1·0*	–249·03	768·65
		0·67	0·0696	0·0016	1·0*	–249·02	768·63
90%—10%	0	0·15	0·0203	0·0023	1·0*	–250·60	771·80
		0·67	0·0203	0·0023	1·0*	–250·65	771·89
	0·33	0·15	0·0243	0·0023	1·0*	–249·88	770·35
		0·67	0·0243	0·0023	1·0*	–249·90	770·38
	0·67	0·15	0·0395	0·0025	1·0*	–249·55	769·69
		0·67	0·0395	0·0025	1·0*	–249·55	769·69
60%—10%	0	0.15	0·0221	0·0022	1·0*	–249·58	769·74
		0.67	0·0221	0·0022	1·0*	–249·62	769·82
	0·33	0.15	0·0263	0·0022	1·0*	–249·23	769·04
		0.67	0·0263	0·0022	1·0*	–249·25	769·08
	0·67	0.15	0·0394	0·0023	1·0*	–249·05	768·68
		0.67	0·0394	0·0023	1·0*	–249·05	768·69

± Percentage MSM with above- and below-average partner acquisition

† Assortativity with regard to sexual activity; ϵ = 0 denotes random mixing

‡ Assortativity with regard to preference for insertive/receptive anal sex; ϕ = 0·15 denotes random mixing

§ β_{01} denotes penile-to-anal transmissibility; β_{10} denotes anal-to-penile transmissibility

¶ Composite fit to H2M baseline and follow-up data

* Upper bound of estimation interval

Table C18. ML estimates of SIS33L model parameters informed by baseline infection prevalence

Sexual activity ±	ϵ †	ϕ ‡	β_{01} §	β_{10} §	ϱ (yr^{-1})	log Likelihood	AIC ¶
80%—20%	0	0·15	0·1189	0·0032	0·514	–248·95	769·59
		0·67	0·1200	0·0032	0·510	–248·97	769·61
	0·33	0·15	0·1880	0·0033	0·470	–249·12	769·92
		0·67	0·1897	0·0033	0·467	–249·12	769·93
	0·67	0·15	0·4447	0·0034	0·416	–249·27	770·22
		0·67	0·4458	0·0034	0·415	–249·27	770·22
90%—10%	0	0·15	0·0448	0·0043	0·789	–249·33	770·33
		0·67	0·0451	0·0043	0·777	–249·33	770·34
	0·33	0·15	0·0604	0·0045	0·682	–249·26	770·20
		0·67	0·0607	0·0045	0·675	–249·26	770·21
	0·67	0·15	0·1148	0·0053	0·578	–249·37	770·43
		0·67	0·1150	0·0053	0·576	–249·38	770·43
60%—10%	0	0.15	0·0633	0·0049	0·531	–248·86	769·40
		0.67	0·0636	0·0049	0·526	–248·86	769·41
	0·33	0.15	0·0826	0·0050	0·501	–248·86	769·40
		0.67	0·0825	0·0050	0·501	–248·86	769·40
	0·67	0.15	0·1408	0·0056	0·453	–248·89	769·46
		0.67	0·1409	0·0056	0·451	–248·89	769·46

± Percentage MSM with above- and below-average partner acquisition

† Assortativity with regard to sexual activity; ϵ = 0 denotes random mixing

‡ Assortativity with regard to preference for insertive/receptive anal sex; ϕ = 0·15 denotes random mixing

§ β_{01} denotes penile-to-anal transmissibility; β_{10} denotes anal-to-penile transmissibility

¶ Composite fit to H2M baseline and follow-up data

* Upper bound of estimation interval

Table C19. ML estimates of SIS10L model parameters informed by baseline infection prevalence

Sexual activity ±	ϵ †	ϕ ‡	β_{01} §	β_{10} §	ϱ (yr^{-1})	log Likelihood	AIC ¶
80%—20%	0	0·15	0·5346	0·0054	0·335	–249·23	764·36
		0·67	0·5429	0·0054	0·332	–249·23	764·37
	0·33	0·15	0·8114	0·0051	0·323	–249·33	764·55
		0·67	0·8171	0·0051	0·322	–249·32	764·55
	0·67	0·15	0·9999	0·0038	0·405	–249·69	765·28
		0·67	0·9999	0·0038	0·405	–249·69	765·28
90%—10%	0	0·15	0·1937	0·0141	0·359	–249·11	764·12
		0·67	0·1963	0·0140	0·353	–249·11	764·12
	0·33	0·15	0·2528	0·0147	0·354	–249·14	764·17
		0·67	0·2558	0·0147	0·349	–249·13	764·17
	0·67	0·15	0·4446	0·0180	0·341	–249·23	764·36
		0·67	0·4469	0·0180	0·338	–249·22	764·34
60%—10%	0	0.15	0·2539	0·0109	0·327	–248·82	763·54
		0.67	0·2580	0·0107	0·322	–248·83	763·56
	0·33	0.15	0·3276	0·0109	0·322	–248·83	763·56
		0.67	0·3314	0·0107	0·319	–248·83	763·57
	0·67	0.15	0·5245	0·0112	0·314	–248·84	763·57
		0.67	0·5278	0·0109	0·312	–248·84	763·57

± Percentage MSM with above- and below-average partner acquisition

† Assortativity with regard to sexual activity; ϵ = 0 denotes random mixing

‡ Assortativity with regard to preference for insertive/receptive anal sex; ϕ = 0·15 denotes random mixing

§ β_{01} denotes penile-to-anal transmissibility; β_{10} denotes anal-to-penile transmissibility

¶ Composite fit to H2M baseline and follow-up data

* Upper bound of estimation interval

Table C20. ML estimates of SIR33L model parameters informed by baseline infection prevalence

Sexual activity ±	ϵ †	ϕ ‡	β_{01} §	β_{10} §	ϱ (yr^{-1})	log Likelihood	AIC ¶
80%—20%	0	0·15	0·9999	0·0757	0·717	–248·62	767·84
		0·67	0·9999	0·0762	0·715	–248·62	767·83
	0·33	0·15	0·9999	0·0632	0·892	–248·81	768·20
		0·67	0·9999	0·0635	0·890	–248·81	768·21
	0·67	0·15	0·9999	0·1004	1·0*	–250·34	771·27
		0·67	0·9999	0·1003	1·0*	–250·34	771·27
90%—10%	0	0·15	0·2621	0·0273	0·812	–248·45	767·48
		0·67	0·2631	0·0274	0·810	–248·45	767·48
	0·33	0·15	0·3395	0·0299	0·799	–248·45	767·49
		0·67	0·3403	0·0299	0·798	–248·46	767·50
	0·67	0·15	0·4904	0·0331	0·789	–248·46	767·50
		0·67	0·4894	0·0329	0·793	–248·47	767·52
60%—10%	0	0.15	0·6187	0·0289	0·803	–248·60	767·79
		0.67	0·6214	0·0289	0·801	–248·60	767·79
	0·33	0.15	0·9170	0·0311	0·800	–248·64	767·86
		0.67	0·9204	0·0311	0·799	–248·64	767·87
	0·67	0.15	0·9999	0·0254	1·0*	–248·94	768·46
		0.67	0·9999	0·0253	1·0*	–248·94	768·46

± Percentage MSM with above- and below-average partner acquisition

† Assortativity with regard to sexual activity; ϵ = 0 denotes random mixing

‡ Assortativity with regard to preference for insertive/receptive anal sex; ϕ = 0·15 denotes random mixing

§ β_{01} denotes penile-to-anal transmissibility; β_{10} denotes anal-to-penile transmissibility

¶ Composite fit to H2M baseline and follow-up data

* Upper bound of estimation interval

Table C21. ML estimates of SIR10L model parameters informed by baseline infection prevalence

Sexual activity ±	ϵ †	ϕ ‡	β_{01} §	β_{10} §	ϱ (yr^{-1})	log Likelihood	AIC ¶
80%—20%	0	0·15	0·9999	0·8813	1·0*	–293·59	857·76
		0·67	0·9999	0·8604	1·0*	–293·71	858·01
	0·33	0·15	0·9999	0·9999	1·0*	–298·21	867·01
		0·67	0·9999	0·9999	1·0*	–298·35	867·28
	0·67	0·15	0·9999	0·9999	1·0*	–307·99	886·57
		0·67	0·9999	0·9999	1·0*	–308·11	886·80
90%—10%	0	0·15	0·9999	0·2279	1·0*	–273·19	816·97
		0·67	0·9999	0·2264	1·0*	–273·22	817·02
	0·33	0·15	0·9999	0·2837	1·0*	–274·40	819·39
		0·67	0·9999	0·2819	1·0*	–274·43	819·44
	0·67	0·15	0·9999	0·3880	1·0*	–275·87	822·33
		0·67	0·9999	0·3859	1·0*	–275·89	822·36
60%—10%	0	0.15	0·9999	0·6600	1·0*	–284·22	839·02
		0.67	0·9999	0·6376	1·0*	–284·30	839·19
	0·33	0.15	0·9999	0·9999	1·0*	–286·52	843·62
		0.67	0·9999	0·9999	1·0*	–286·63	843·86
	0·67	0.15	0·9999	0·9999	1·0*	–290·85	852·29
		0.67	0·9999	0·9999	1·0*	–290·95	852·48

± Percentage MSM with above- and below-average partner acquisition

† Assortativity with regard to sexual activity; ϵ = 0 denotes random mixing

‡ Assortativity with regard to preference for insertive/receptive anal sex; ϕ = 0·15 denotes random mixing

§ β_{01} denotes penile-to-anal transmissibility; β_{10} denotes anal-to-penile transmissibility

¶ Composite fit to H2M baseline and follow-up data

* Upper bound of estimation interval
