# Supplementary material for: Potential effectiveness of prophylactic HPV immunization for men who have sex with men in the Netherlands: A multi-model approach
Source: PLoS Med. 2019 Mar 4;16(3):e1002756. doi: 10.1371/journal.pmed.1002756 (PMC6398832; doi:10.1371/journal.pmed.1002756)
Supplement: S4 Text — (PDF) [file pmed.1002756.s021.pdf]

S4 TEXT: MODEL-AVERAGED PREDICTIONS

PART IV OF IV OF THE SUPPLEMENTARY ANNEX TO

POTENTIAL EFFECTIVENESS OF PROPHYLACTIC HPV IMMUNIZATION FOR MEN WHO HAVE SEX WITH MEN IN THE NETHERLANDS: A MULTI-MODEL APPROACH.

Johannes A Bogaards*, Sofie H Mooij, Maria Xiridou, Maarten F Schim van der Loeff

* Author to whom correspondence should be addressed: hans.bogaards@rivm.nl

TABLE OF CONTENTS

Supplement IV: Model-averaged predictions *page 2*

S4.1 Vaccination scenarios *page 3*

Supplementary References *page 5*

Legends to Supplementary Figures *page 6*

Supplement IV: Model-averaged predictions

To form a weighted average of the predicted effectiveness of targeted vaccination from the different models, each model was given a weight w_S according to:

$$w_S = \frac{\exp\left(-\frac{1}{2}AIC_S\right)}{\sum_{S' \in \mathcal{M}} \exp\left(-\frac{1}{2}AIC_S\right)} \quad \text{(Eq.13)}$$

where AIC is the Akaike information criterion, a measure of the relative quality of each model with respect to H2M study data x:

$$AIC_S = 2\dim S - 2\log \mathcal{L}\left(\hat{\theta}_S | x\right) \quad \text{(Eq.14)}$$

Here, $\dim S$ denotes the number of free parameters in the ML procedure, and its inclusion acts as a penalty against overfitting. In general, given a set of candidate models $\mathcal{M}$, the preferred model is the one with the lowest AIC value. As a rule of thumb, models with an AIC difference of ≥10 relative to this model hardly have empirical support and can be omitted from further consideration, as these receive negligible weight in prediction [1,2].

AIC was computed for each model as a composite fit to H2M baseline and follow-up data, according to independence assumptions delineated before. Parameters on the boundary of the parameter space counted as free parameters, meaning that models with suboptimal fit due to bound constrained optimization were penalized both for having suboptimal fit and for having possibly redundant parameters. Resulting AIC values are given in supplementary tables tables C1 to C21 at end of S3 Text. The relative quality of the models with respect to H2M study data was more dependent on the assumed natural history of HPV16 infection than on the sexual contact structure of

the model (S8 Fig). Note that the SIS10L models provided comparatively good fit to H2M study data and accordingly received strongest weights in model-averaged prediction. In contrast, the SIR10L models gave invariably poor fit and were discarded from the set of models used for prediction.

S4.1 Vaccination scenarios

We assumed HPV vaccine uptake rates similar to hepatitis B (HepB) vaccine among MSM in the Netherlands, prior to the inclusion of HepB vacccine into the national childhood immunization program as of 2011. Data from the first seven years (2002–2009) of the national HepB vaccination campaign for MSM in the Netherlands were used to estimate vaccine uptake rates. Annual vaccine uptake was calculated as the number of first vaccinations reported at the vaccination program by five-year age groups divided by the estimated number of eligible MSM in the Netherlands in the corresponding age group. The number of vaccinations was adjusted from the number of MSM receiving the first vaccine dose to account for the total number of expected doses during the observation period. Further details can be found in the appendix to the original publication [3].

For application to our models, we fitted a Gamma function to the estimated HepB vaccine uptake rates among 15- to 70-year-old MSM up to 2010 in the Netherlands (S9 Fig). The original publication also investigated how selective HepB vaccination for MSM could be improved. One scenario, dubbed “double vaccination”, assumed that twice as many vaccinations were administered as had in fact been reported at the vaccination program. We adopted this scenario to assess the impact of an increased HPV vaccine acceptance among MSM as compared to the estimated uptake of HepB vaccine, by simply doubling the fitted age-specific vaccine uptake rates.

To model a combined strategy of preadolescent and targeted vaccination, we assumed that a certain percentage of individuals enter the at-risk population already vaccinated, i.e. prior to sexual debut. The non-vaccinated individuals may still opt to get vaccinated at a later age, according to the age-specific vaccine uptake rates described above. Note that we neglected the possibility that individuals who were unsuccessfully vaccinated in preadolescence, might apply for a booster vaccination at a later age. This possibility has little bearing on the effectiveness of a combined strategy, as we assume high prophylactic efficacy of vaccination in preadolescence.

SUPPLEMENTARY REFERENCES

1. Burnham KP, Anderson DR. Model Selection and Multimodel Inference: A Practical Information-Theoretic Approach (2nd ed). New York, NY: Springer-Verlag; 2002.

2. Claeskens G, Hjort NL. Model Selection and Model Averaging (1st ed). Cambridge, UK: Cambridge University Press; 2008.

3. Xiridou M, van Houdt R, Hahné S, Coutinho R, van Steenbergen J, Kretzschmar M. Hepatitis B vaccination of men who have sex with men in the Netherlands: should we vaccinate more men, younger men, or high-risk men? Sex Transm Infect. 2013; [Data supplement 1 - Online appendix]. doi: 10.1136/sextrans-2012-050900.

LEGENDS TO SUPPLEMENTARY FIGURES

S8 Figure. Relative quality of models used in prediction. The quality of models (acronyms as in Table 2) with respect to H2M study data was assessed by the Akaike information criterion (AIC). In computation, only relative differences with respect to the model with the lowest AIC are relevant and these are plotted in A) according to presumed natural history (models ordered as in Table 2), and in B) according to sexual contact structure (ordered as in figure S6C).

S9 Figure. Age-specific vaccine uptake among MSM. HPV vaccination rates (red lines) in base-case model predictions and in sensitivity analysis (thin line). Base-case uptake was based on historic uptake for hepatitis B (HepB) vaccine among 15- to 70-year-old MSM in the Netherlands. Note that the slightly increased HepB vaccine uptake rate among >70-year-old MSM was not incorporated in model predictions.
